# Chemically Engineered Immune Cell‐Derived Microrobots and Biomimetic Nanoparticles: Emerging Biodiagnostic and Therapeutic Tools

**DOI:** 10.1002/advs.202002499

**Published:** 2021-03-01

**Authors:** Leila Pourtalebi Jahromi, Mohammad‐Ali Shahbazi, Aziz Maleki, Amir Azadi, Hélder A. Santos

**Affiliations:** ^1^ Drug Research Program Division of Pharmaceutical Chemistry and Technology Faculty of Pharmacy University of Helsinki Helsinki FI‐00014 Finland; ^2^ Pharmaceutical Sciences Research Center Shiraz University of Medical Sciences Shiraz 71468‐64685 Iran; ^3^ Zanjan Pharmaceutical Nanotechnology Research Center (ZPNRC) Zanjan University of Medical Sciences Zanjan 45139‐56184 Iran; ^4^ Department of Pharmaceutics School of Pharmacy Shiraz University of Medical Sciences Shiraz 71468‐64685 Iran; ^5^ Helsinki Institute of Life Science (HiLIFE) University of Helsinki Helsinki FI‐00014 Finland; ^6^ Present address: Helmholtz Institute for Pharmaceutical Research Saarland Helmholtz Centre for Infection Research Biogenic Nanotherapeutics Group Campus E8.1 Saarbrücken 66123 Germany

**Keywords:** artificial dendritic cell and extracellular vesicle, biomimetic drug delivery, engineered immune cell, immune cell membrane, nanomedicine

## Abstract

Over the past decades, considerable attention has been dedicated to the exploitation of diverse immune cells as therapeutic and/or diagnostic cell‐based microrobots for hard‐to‐treat disorders. To date, a plethora of therapeutics based on alive immune cells, surface‐engineered immune cells, immunocytes’ cell membranes, leukocyte‐derived extracellular vesicles or exosomes, and artificial immune cells have been investigated and a few have been introduced into the market. These systems take advantage of the unique characteristics and functions of immune cells, including their presence in circulating blood and various tissues, complex crosstalk properties, high affinity to different self and foreign markers, unique potential of their on‐demand navigation and activity, production of a variety of chemokines/cytokines, as well as being cytotoxic in particular conditions. Here, the latest progress in the development of engineered therapeutics and diagnostics inspired by immune cells to ameliorate cancer, inflammatory conditions, autoimmune diseases, neurodegenerative disorders, cardiovascular complications, and infectious diseases is reviewed, and finally, the perspective for their clinical application is delineated.

## Graphical abstract

Synthetic biology is a rapidly growing technology that has an enormous potential for the development of novel therapeutics and imaging tools. This review focuses on the latest progress in the design of immune cell‐derived microrobots and biomimetic nanoparticles as engineered biocarriers to ameliorate hard‐to‐treat diseases.

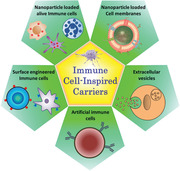

## 1 Introduction

Developments in medical sciences have revealed that the etiology of many diseases, especially those hard‐to‐treat varieties like cancers and central nervous system (CNS) disorders emanates from the subcellular level, where conventional biology meets modern ingenuity at the nanoscale. The advent and rapid uptake of nanomedicine in pharmaceutical research has enabled medical scientists to manipulate biological functions for better diagnosis and treatment of various diseases.^[^ ^1^ ^]^ Nanotherapeutics have various tunable physicochemical properties offering superior efficiency; however, there are still challenges for their straightforward clinical translation. One of these challenges is their recognition by the immune system as invading foreign bodies. A creative solution to this problem is to exploit the body's components, in particular various elements of the immune system to make stealth medicines. These novel therapeutics will provide a promisingly longer circulation time, as well as other functional properties depending on the planned therapeutic purposes.^[^ ^2^ ^]^ Such biomimetic formulations are also definable under the emerging paradigm of personalized medicine in which it is conceivable to take advantage of the patients’ own cells. Moreover, mimicking the biological functions of the immune system in novel drug delivery formulations will improve pharmacodynamic and pharmacokinetic properties of cargo.^[^ ^3^ ^]^ For example, the homing capacity of immune cells in inflamed or malignant tissues enables them to interact specifically with damaged cells, making them good candidates for carrying imaging and therapeutic agents.^[^ ^4^ ^]^ Employing immune cells also empowers drug delivery systems (DDSs) through circumventing physiological barriers like the blood–brain barrier (BBB) for CNS drug delivery^[^ ^5^ ^]^ or overcoming the drug‐resistance of cancer tissues. Another privilege of immune cells is the crosstalk among themselves for the purpose of activation or suppression. This process can be manipulated for more effective vaccine production, as well as immunostimulant and immunosuppressant therapeutics.^[^ ^4^, ^6^ ^]^ One more important feature of immune cells is that they have active defensive compartments against pathogens and tumor cells and this can be engineered to produce highly vigorous responses in the fight to mitigate pathological conditions.^[^ ^7^ ^]^ The last but not least significant characteristic of immune cells to be mentioned is their phagocytosis ability, which enables them to take up and carry a considerable amount of various payloads, such as macromolecules, hydrophobic and hydrophilic agents, nano‐ and microparticles, and even cytotoxic agents.^[^ ^8^ ^]^ In addition to these distinctive qualities, the development of immune cell‐inspired DDSs share other advantages like the capacity to be surface‐engineered and designed for controlled drug release as with other nanotechnology‐based DDSs.

Despite the aforementioned advantages, the challenges to produce and use immune cell‐based DDSs, if bench to bedside transition is the final goal, should be considered. These include scale‐up manufacturing, reproducibility, difficult and expensive procedures for cell separation and purification, wise control over cells’ phenotypes, technological limitations of payload encapsulation, and the possibility of unfavorably damping or hyperactivating the patients’ immune system.^[^ ^3^, ^4^ ^]^

Herein, we review the latest attempts for harnessing the exceptional characteristics of immune cells to design eminent immune cell‐based synthetic diagnostics, therapeutics, and novel DDSs. These include therapeutics based on alive immune cells,^[^ ^9^ ^]^ surface‐engineered immune cell‐based systems,^[^ ^10^ ^]^ drug delivery platforms taking advantage of immune cell membranes,^[^ ^11^ ^]^ therapeutic formulations based on extracellular vesicles (ECVs) or exosomes (Exos) of immune cells,^[^ ^12^ ^]^ and finally, artificial immune cells.^[^ ^13^ ^]^ We provide a deep look into in vitro characteristics and in vivo behavior of these systems and outline the current state‐of‐the‐art and the future perspective.

## 2 Ex Vivo Manipulated Alive Immune Cells

A variety of immune cells with different properties have the potential to uptake (through straightforward internalization, phagocytosis, or transfection), carry, and deliver therapeutics and diagnostics. Recently, dendritic cells (DCs), macrophages (M*ϕ*s), monocytes (MCs), neutrophils (NEs), and lymphocytes, especially T cells (TCs) have attracted the most scientific attention for this goal.^[^ ^14^ ^]^ In this regard, the following sections offer a review of the literature focused on alive immune cells applied as therapeutic carriers to treat various diseases.

### 2.1 Nanoengineered and Drug‐Loaded Alive Dendritic Cells

DCs must be engineered ex vivo if the aim is to transfer therapeutic agents into the cells. As a starting step, DCs should be extracted directly from peripheral blood or bone marrow, but due to their scarce population in the blood circulation, the trending alternative strategy to obtain a sufficient number of DCs is to harvest the MCs or cluster of differentiation (CD) 34^+^ hematopoietic progenitor cells and differentiate them into DCs.^[^ ^15^ ^]^ Priming MCs to mature DCs can be monitored by quantifying particular cytokines (e.g., interleukin (IL)‐12 and interferon‐gamma (IFN‐*γ*)) as well as checking the surface presentation of CD80, CD83, and CD86 co‐stimulatory signals, C‐C chemokine receptor (CCR) 7, and major histocompatibility complex (MHC) II ligand.^[^ ^16^ ^]^

Substantial challenges to address when employing alive DC‐based DDSs include the optimization of particulate systems used in the cell engineering to increase their uptake efficiency into DCs, proper design of particles with minimal toxicity to DCs, and achieving a proper pharmacokinetic profile for the DCs after manipulation. Moreover, challenges of translating the results of ex vivo and in vivo animal experiments to clinical settings should be considered.^[^ ^17^ ^]^

Poly(lactic‐*co*‐glycolic acid) (PLGA) particles have been widely utilized for antigen or therapeutic loading into DCs.^[^ ^18^ ^]^ PLGA is a nontoxic degradable polymer used for controlled drug release due to its low swelling in a biological environment.^[^ ^19^ ^]^ It has been reported that antigensʼ loading into the PLGA particles and then transferring them to DCs would protect them from degradation and nonspecific dispersion in body fluids, leading to efficient antigen transfer to TCs. The negatively charged surface of PLGA nanoparticles (NPs) and a size smaller than 500 nm were reported as optimum conditions for being internalized into DCs.^[^ ^19^, ^20^ ^]^ An interesting study demonstrated the coencapsulation of ovalbumin (OVA) or E7 peptide tumor antigen and a Toll‐like receptor 3 (TLR 3) agonist as an adjuvant within spherical PLGA NPs with a size of 200 ± 3.69 nm and a zeta potential of about −30 mV. The loading efficiency of NPs for antigen and adjuvant were 90% and 50%, respectively, and sustained antigen release was observed over 30 days. The cellular uptake of the NPs by DCs was more than 80% and their toxic effect on DCs was negligible. Compared to the control groups, the DCs treated with the antigen‐adjuvant encapsulated PLGA NPs expressed significantly higher stimulatory markers ex vivo. Besides, following intravenous (i.v.) injection, they stimulated cytotoxic TCs more efficiently, leading to tumor shrinkage and increased survival of tumor‐bearing mice.^[^ ^21^ ^]^ Li et al. have similarly investigated the effect of DCs exposed to tumor antigens loaded in spherical PLGA NPs on TC activation.^[^ ^22^ ^]^ The DCs internalized these NPs within 15 min and reached a saturation level for uptake in 45 min post‐treatment. Compared to the free antigen‐pulsed DCs, enzyme‐linked immunospot (ELISpot) assay revealed that sustained antigen presentation to DCs, provided by PLGA NPs, induces stronger TC activation and a better cancer cell killing effect. In another study, Iranpour et al. encapsulated breast cancer cell lysate as antigen within the spherical PLGA NPs and loaded them into DCs.^[^ ^23^ ^]^ It was observed that NP‐loaded DCs can improve DC maturation and differentiation. The coculturing of the activated DCs with TCs resulted in increased TC proliferation and the release of inflammatory cytokines when the DC:TC ratio was 1:10. Encapsulating tumor cell membrane lysate within PLGA NPs and decorating the particles’ surface with a TLR9 agonist also led to enhanced DC maturation and subsequent TC activation in another investigation.^[^ ^24^ ^]^

**Figure 1:**
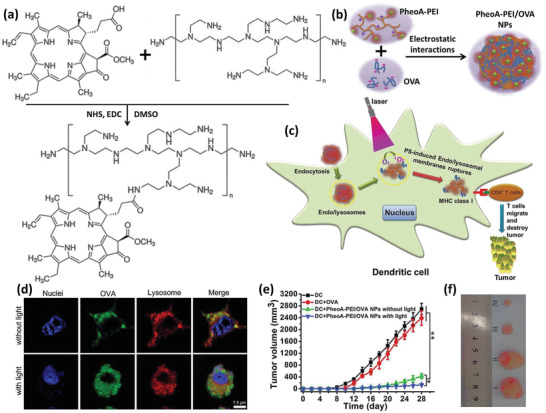
Light responsive NP‐loaded DC‐based cancer vaccine. a) The photosensitizer is grafted to the polymer via a conventional carbodiimide reaction. b) Positive chains of PheoA‐PEI interact with negatively charged cancer antigen, OVA, and spherical antigen containing NPs are formed. c) NPs were endocytosed and located within the endolysosomal compartments until being irradiated. After that, because of PheoA‐induced ROS production, antigens were liberated in the cytoplasm and presented to T cells. d) Fluorescent signals of antigen and lysosome were colocalized before being irradiated using a 670 nm laser source, proceeded by OVA being dispersed within the cytoplasm. e,f) Tumor volume in mice receiving each of the test or control formulations. In the photographs, I, II, III, and IV indicate the tumors of animals that received nontreated DCs, OVA‐treated DCs, DCs incubated with PheoA‐PEI–OVA without irradiation, and DCs incubated with PheoA‐PEI–OVA with irradiation, respectively. Reproduced with permission.^[^ ^26^ ^]^ Copyright 2017, American Chemical Society.

Another polymer used to transfer tumor antigens into DCs for further use as an antitumor vaccination is polyethyleneimine (PEI). With primary, secondary, and tertiary amines, PEI is a cationic water‐soluble polymer extensively studied for carrying, protecting, and delivering negatively charged molecules, especially nucleic acids. Based on the branching degree and its molar mass, PEI shows severe cytotoxicity that must be considered in formulation design.^[^ ^25^ ^]^ Zhang et al. have developed a photoresponsive DDS based on PEI NPs and DCs. They grafted hydrophobic pheophorbide A (PheoA) photosensitizer to hydrophilic PEI in equal molar ratios (**Figure** **1**a).^[^ ^26^ ^]^ PheoA can generate reactive oxygen species (ROS), which aids photochemical internalization of the NPs. The combination of these two materials resulted in the self‐assembly of NPs with a size of about 7 nm. Next, OVA as a cancer antigen was added (carrier to antigen mass ratio 1:2) and the spherical nanostructures with a size between 100 and 200 nm were formed based on the electrostatic interactions (Figure 1b). While most of the NPs were localized in the lysosome before light irradiation of 670 nm with the intensity of 0.5 J cm^−2^, they were mainly localized in the cytoplasm after laser irradiation for 100 s. Figure 1c schemes the light‐induced antigen release within the cytoplasm and the pattern was observable in the fluorescence images of NP‐loaded DCs before and after irradiation (Figure 1d). Although the pH‐buffering effect of PEI leads to an osmotic disruption of endolysosomes, little endosomal escape was detected for NPs without irradiation. To evaluate in vivo antitumor efficiency of the developed vaccine, mice were classified into four groups based on receiving 1) nontreated DCs, 2) OVA‐treated DCs, 3) DCs incubated with PheoA‐PEI–OVA without irradiation, and 4) DCs incubated with PheoA‐PEI–OVA with irradiation. Significant increase in cytotoxic TC activity and proliferation as well as a significant reduction in tumor growth, tumor volume, and tumor mass were observed in the latter two groups as compared to the other groups. Moreover, antitumor responses were significantly higher for laser irradiated DCs exposed to PheoA‐PEI–OVA NPs as compared to nonirradiated DCs, mainly interpreted by the light‐induced endosomal escape of the antigen. Figure 1e,f both reflect the distinctive in vivo results between NP‐loaded versus pristine DCs and the irradiated DCs versus those receiving no laser irradiation.

Chitosan, known for its low immunogenicity, high biodegradability, and desirable biocompatibility, is another favorable cationic polymer for immunity modulation.^[^ ^27^ ^]^ Daneshmandi et al. have engineered DCs by chitosan NPs containing the total tumor messenger ribonucleic acid (mRNA) and either of CD40 mRNA or inducible co‐stimulatory ligand (ICOSL) mRNA for antitumor vaccine development.^[^ ^28^ ^]^ It was revealed that more than 95% of mRNA could be incorporated into NPs with a final diameter of ≈35 nm. The efficiency of the chitosan NPs for mRNA transfection to DCs was compared to that of lipofectamine. Although lipofectamine was superior in the in vitro tests, DCs being pulsed with chitosan‐mRNA NPs induced stronger tumor suppression and more delay in the tumor growth, leading to increased animal survival in the in vivo experiments on mammary carcinoma mouse model. DCs containing chitosan‐mRNA NPs led to a higher activation of cytotoxic TCs in vivo in comparison to lipofectamine‐transfected DCs, indicating a more vigorous immune response for cancer therapy.

**Figure 2:**
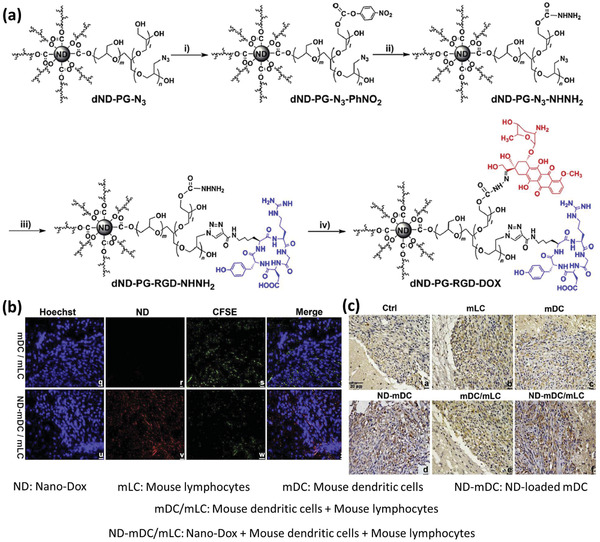
Dendritic cell‐mediated delivery of ND‐PG‐RGD‐DOX to glioblastoma. a) Synthesis procedures of the ND‐PG‐RGD‐DOX nanocomposite from dND‐PG‐N_3_. The procedures of dND‐PG‐N_3_ synthesis include 1) glycidol, 140 °C, 20 h; 2) *p*‐TsCl, NaOH, 0 °C and room temperature overnight; 3) NaN_3_, 90 °C, overnight. Procedures for the modification of dND‐PG‐N_3_ with RGD and DOX include i) bis(4‐nitrophenyl) carbonate, triethylamine, room temperature, 24 h; ii) hydrazine monohydrate, 90 °C, overnight; iii) RGD propargyl amide, copper (II) sulfate pentahydrate, sodium ascorbate, room temperature, 48 h; iv) DOX hydrochloride, pH 7, 50 °C, 24 h. Adapted with permission.^[^ ^29^ ^]^ Copyright 2014, Elsevier. b) Fluorescent imaging of mouse brain tissue sections shows the presence of cells and mDC‐delivered NanoDOX in orthotopic glioblastoma cell xenografts. Nucleus was stained with blue Hoechst 33342 fluorescent agent. Red fluorescence came from DOX or NanoDOX and green fluorescence from the CFSE‐labeled mDC. c) Immunohistological staining of caspase‐3 activity, as a marker of apoptosis, in mouse orthotopic glioblastoma cell xenografts treated differently. Caspase‐3 activity was dramatically intensified in xenografts that received NanoDOX‐mDC or NanoDOX‐mDC/mLC, but was mildly increased in xenografts that were treated with mLC or mDC/mLC. Adapted with permission.^[^ ^30^ ^]^ Copyright 2018, Elsevier.

Another research group has taken advantage of a previously developed nanoplatform, called NanoDox, for loading doxorubicin (DOX) into DCs.^[^ ^29^ ^]^ NanoDox was composed of a 4–5 nm nanodiamond (ND) core with a surface coating of hyperbranched polyglycerol (PG), in which DOX as an antitumor agent and arginine–glycine–aspartic acid (RGD) as a targeting moiety were covalently attached to its surface (**Figure** **2**a). In the first step, PG was grafted on to a ND via ring‐opening polymerization of glycidol using hydroxyl groups on the surface. As for RGD conjugation, partial hydroxyl groups on ND‐PG were first converted to tosyl groups, which were then reacted with sodium azide to generate azido groups (ND‐PG‐N_3_). Next, the ND‐PG‐N_3_ was reacted with RGD peptide bearing an alkynyl terminal through copper‐catalyzed click reaction to yield ND‐PG‐RGD. As for DOX conjugation, part of hydroxyl groups on ND‐PG were converted to hydrazine groups (ND‐PG‐NH‐NH_2_) and the drug was conjugated through a hydrazone bond, which could be cleaved under a weak acidic condition. The hydrodynamic diameter of the final NPs was estimated to be 83.9 ± 32.3 nm and a dose of NanoDox equal to 2 µg mL^−1^ DOX was selected as optimum to be loaded into DCs. It was observed that the DCs’ ability to migrate was not damaged after NanoDox loading and the cargo was released in the form of NanoDox and not the free DOX molecules. In a coculture of glioblastoma cells with either the NanoDox or DOX alone, it was confirmed that the NanoDox had more potency to induce the expression of danger‐associated molecular patterns (DAMPs) in the tumor cells, which consequently attract the immune cells to the tumor site. In the next step, lymphocytes were added to the culture media containing DCs and tumor cells with or without NanoDox, and it was revealed that in the presence of NanoDox, lymphocytes were activated more, reflected in the higher expression of stimulatory surface markers on the DCs and increased release of inflammatory cytokines. In vivo studies on glioblastoma‐bearing mice showed that i.v. injection of NanoDox‐loaded DCs, followed by the injection of lymphocytes after 48 h results in strong colocalization of the DCs and NanoDox within the tumor (Figure 2b), leading to a significant decrease in tumor‐induced immunosuppression and superior antitumor efficiency, confirmed by tumor cell apoptosis (Figure 2c).^[^ ^30^ ^]^

In addition to nanoengineering, DCs have shown the ability to carry therapeutic molecules that are not embedded in a NP. Recently, Bonomi et al. have reported using DCs to deliver paclitaxel (PTX).^[^ ^31^ ^]^ They incubated 1 µg mL^−1^ of PTX with DCs for 24 h and further assessed drug release from DCs over 7 days. Drug release was time‐dependent and almost 90% of the total drug was released from DCs within 72 h. It also revealed that PTX completely inhibited DC proliferation, while it had minor effects on their viability. Moreover, the coculturing of PTX‐loaded DCs with a human glioblastoma cell line significantly blocked the proliferation of tumor cells.^[^ ^31^ ^]^

### 2.2 Nanoengineered and Drug‐Loaded Alive M*ϕ*s

M*ϕ* are the most common immune cells investigated for tuning aberrant physiological functions or as a carrier for antigens, genes, and other therapeutics. Like DCs, M*ϕ*s can be activated ex vivo by immunostimulants, but some challenges impede using M*ϕ*s as a cargo carrier, especially in in vivo studies. An important issue is the difficulty to control the environment where M*ϕ*s are recruited which directly influences their phenotype and activity, and hence it might make the comparison of results from different studies inaccurate.^[^ ^32^ ^]^ In this regard, there are concerns that M*ϕ*s being educated to induce antitumor effects turn into protumoral M*ϕ*s, influenced by the tumor microenvironment conditions and contribute to tumor survival.^[^ ^33^ ^]^ Another concern is the in vivo biodistribution of M*ϕ*s and their homing capabilities. It is reported that intravenously injected cargo‐loaded M*ϕ*s were primarily recruited in the lung, liver, and spleen, while they were expected to accumulate in the tumor sites.^[^ ^34^ ^]^ Moreover, drug release from M*ϕ*s should be carefully studied, since passive drug diffusion via the cell membrane, release through cell death, and release through Exos that have been reported in the literature, provide different profiles of cargo release and alter its bioavailability.^[^ ^35^ ^]^ To overcome the above‐mentioned obstacles, several studies have engineered M*ϕ*s to develop more controllable carriers.^[^ ^32^, ^36^ ^]^ A variety of M*ϕ*‐based therapeutic systems have taken advantage of micro‐ and NPs. Loading the therapeutic agent within a particulate system not only protects the host cell but also prevents cargo damage by components of the lysosomal compartment, as well as providing a controlled release for the cargo.^[^ ^4^, ^37^ ^]^ It is shown that the particle's physicochemical properties (e.g., hydrophobicity, surface charge, shape, and size) could dictate the uptake efficiency by M*ϕ*s, as well as their route of uptake.^[^ ^38^ ^]^ The following paragraphs will have a closer look into the published literature, reporting the application of alive genetically or nongenetically engineered M*ϕ*s as vaccines, diagnostic tools, and gene or drug carriers, whether in their free form or incorporated within the micro‐ and NPs.

**Figure 3:**
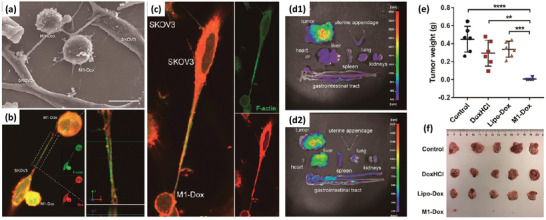
a) Tunneling nanotubes between M1‐Dox and SKOV3 ovarian carcinoma cells. b,c) Red fluorescence of DOX presenting inside green tunneling nanotube connected cells were observed by confocal laser scanning microscopy (CLSM). Phalloidin (green) was used to stain F‐actin as the vital component of tunneling nanotubes, and DOX emitted intrinsic red fluorescence. d) The fluorescence images of the tumor and excised organs 24 h after the i.p. injection of d1) M1‐DiD and d2) Lipo‐DiD. Color bars show the fluorescent intensity. High accumulation of the Lipo‐DiD in the liver is observable. e,f) Final tumor weights and photographs of metastatic nodules collected on several peritoneal organs after different treatments for 28 days. Adapted with permission.^[^ ^39^ ^]^ Copyright 2018, American Chemical Society.

Several reports are available incorporating drug molecules or genes directly within the M*ϕ*s.^[^ ^35^, ^39^ ^]^ A groundbreaking study conducted by Guo et al. has recently unraveled another method of drug transport from M*ϕ*s to tumor cells, using the free form of the drug molecule.^[^ ^39^ ^]^ They developed DOX‐loaded not stimulated murine M*ϕ* cells (M0‐Dox) as well as DOX‐loaded INF*γ* and lipopolysaccharides (LPSs) stimulated murine M*ϕ* cells (M1‐Dox). M0 cells exhibited about 30% less drug loading and significant drug leakage (60% vs 20%) within 12 h compared to M1 cells. Moreover, their surface zeta potential, surface roughness, cell activity, and cell viability decreased significantly compared to the unloaded M0, while M1 M*ϕ*s remained intact concerning these properties. Hence, M1‐Dox was selected for further studies. The antitumor effect of M1‐Dox, and commercial products of DOX hydrochloride (DoxHCL) and liposomal DOX (Lipo‐Dox) were then compared in vitro. While the two commercially available formulations induced tumor invasion, M1‐Dox cells had significant anti‐invasion activity that might be related to their attachment to tumor cells through the cytoskeletal filaments of M*ϕ*s. M1‐Dox cells also strongly inhibited tumor cell growth in a 3D model compared to M0‐Dox and the commercial formulations. Additionally, during the in vitro tests, it was found that the speed of drug transport from M*ϕ*‐based formulations (M1 > M0) to the tumor cells is much faster than the commercial formulations. Using electron microscopy and live‐cell imaging techniques, tunneling nanotubes were discovered between M1‐Dox and the neighboring tumor cells and these tunnels speed up the transfer of DOX to the tumor cells (**Figure** **3**a–c). Mice exhibiting metastatic ovarian carcinoma received intraperitoneal (i.p.) injection of DiD‐labeled M1 macrophages (M1‐DiD) or DiD‐labeled liposomes (Lipo‐DiD) and fluorescence was detected in the tumor and main organs (Figure 3d1,d2). M1‐DiD‐treated mice showed stronger fluorescence intensity in peritoneal tumor tissue as compared to the Lipo‐DiD. Furthermore, other organs of M1‐DiD‐treated mice exhibited significantly lower fluorescence intensity than that of M1‐DiD‐treated mice.^[^ ^40^ ^]^ In addition, M1‐Dox M*ϕ*s migrated to disseminated peritoneal tumor nodes and penetrated much deeper than Lipo‐Dox, resulting in a significant reduction of tumor weight and volume (Figure 3e,f) and a significant increase in animal survival compared to DoxHCL and Lipo‐Dox.

Another report by Fu et al. has investigated the amount of DOX that M*ϕ*s could carry.^[^ ^41^ ^]^ They stated that about 100 µg of free DOX can be loaded within 1 million M*ϕ*s, which is enough to treat cancer in mice, while preloading of DOX in NPs like liposomes would not provide equivalent loading efficiency. DOX was found to have a minimal toxic effect on carrier M*ϕ*s and the cell viability was about 88% after 24 h in 3‐(4,5‐dimethylthiazol‐2‐yl)‐2,5‐diphenyltetrazolium bromide (MTT) test. Furthermore, M*ϕ*s were recruited within the tumor‐bearing lung (metastatic breast cancer model) during the first 0.5 h postadministration. Parallel studies indicated that the DOX‐loaded M*ϕ*s inhibited tumor growth and metastasis, leading to a significant improvement in animal survival compared to the free DOX solution.

A group of scientists reported the use of genetically engineered M*ϕ*s for in situ overexpression of vasoendothelial growth factor 165 (VEGF_165_).^[^ ^42^ ^]^ M*ϕ*s were loaded with a recombinant lentiviral vector that contained human VEGF_165_ and a green fluorescent protein to ameliorate arterial damage in an atherosclerosis‐prone mouse model. Tracking the fluorescence proved that these VEGF overexpressing M*ϕ*s could home in the damaged locale, transform into endothelial cells, and incorporate in the newly formed vessels. These M*ϕ*s produced significantly higher nitric oxide in vitro and in vivo, which has a protective role in arterial injury and averts hyperplasic neointima formation. Another creative idea to take advantage of M*ϕ*s’ mobility to migrate toward and home in the injured site is to use them to carry stem cells for regenerative purposes. A substantial step before loading another living cell into M*ϕ*s would be stopping phagosome maturation after it is formed to avoid any damage to the living cell cargo.^[^ ^43^ ^]^

A considerable number of publications have also reported the loading of NPs and microparticles within M*ϕ*s to harness the biomimicry they provide. A study by Li et al. proved that incorporating the lipophilic molecules of PTX into the M*ϕ*s was not as efficient as when it was first loaded within *N*‐succinyl‐*N*′‐octyl chitosan‐based NPs (final size of 150 nm).^[^ ^35a^ ^]^ The toxicity of the drug on the carrier cells diminished significantly after its loading into the NPs, and slower drug release from the cells was observed. In vivo investigations further demonstrated that the M*ϕ*s had a greater tumor tendency in immunocompetent mice compared to immunocompromised mice because of the difference in their tumor sites’ chemokine secretion pattern. The highest antitumor efficiency belonged to the M*ϕ*s loaded with chitosan‐PTX NPs, followed by PTX‐loaded M*ϕ*s and the free drug solution.

**Figure 4:**
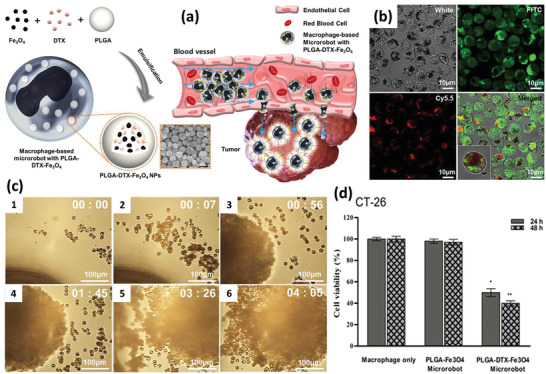
a) Schematic illustration of developing macrophage‐based microrobot with PLGA–DTX–Fe_3_O_4_ (left) and depiction of possible tumor targeting in vivo (right). b) M*ϕ*s (green) that have internalized NPs (red) are distinguished by fluorescence imaging. c) Active targeting of tumor spheroid with magnetically guided M*ϕ*s. d) Toxicity test on the murine colorectal CT‐26 cancer cell line. Reproduced with permission.^[^ ^45d^ ^]^ Copyright 2016, Springer Nature.

Polylactic acid NPs containing PLXSpringer 4032 drug and surface decorated with muramyl tripeptide (MTP) has been reported to increase the uptake efficiency of NPs by M*ϕ*s.^[^ ^44^ ^]^ These NPs were engineered to release the drug in a controllable manner. Within 2 h of being cultured with the M*ϕ*s, the NPs were surface‐bound or internalized by the cells. The MTP decoration increased the NP uptake efficiency by M*ϕ*s from 61% in nondecorated particles to 96% for decorated ones. It was revealed that in a coculture, NP‐loaded M*ϕ*s effectively transferred their content to melanoma cells, leading to significant death of tumor cells.^[^ ^44^ ^]^ PLGA particles have also been used in several studies for engineering M*ϕ*s as therapeutic carriers.^[^ ^45^ ^]^ For example, DOX‐loaded PLGA NPs with a diameter of about 141 nm, polydispersity index (PdI) of 0.086, and zeta potential of −31.7 mV were prepared and incubated with the M*ϕ*s for 2 h. As a result of tumor‐targeting efficiency, DOX accumulation and penetration into the tumor microenvironment of nude mice bearing intracranial U87 glioma was higher when the drug incorporated NPs were loaded into M*ϕ*s.^[^ ^45b^ ^]^ A similar study used a biomimetic DDS composed of DOX‐loaded PLGA NPs, incorporated within M1 M*ϕ*s for glioma treatment (DOX‐PLGA‐M1).^[^ ^45c^ ^]^ PLGA NPs, with an average size of 156.9 ± 7.1 nm and a loading efficiency of 4.35 ± 0.56% for DOX, were incubated with the M1 M*ϕ*s that had a higher NP‐loading capacity as compared to the M0 M*ϕ*s. The final concentration of DOX in the M*ϕ*s was 34 ± 2.3 µg per 5 × 10 ^6^ M*ϕ*s and PLGA NPs were localized in the lysosomal compartment in the M1 cells. The cargo release was expected to occur via Exo secretion. Using M1 instead of M0 M*ϕ*s could significantly improve DOX localization in the tumor environment, resulting in an obvious increase in tumor cell death and animal survival. However, the rate of migration into the tumor site was still a challenge. This was addressed by Han et al. utilizing magnetic NPs accompanied by an external magnetic field.^[^ ^45d^ ^]^ To speed up the migration of drug‐PLGA NP‐M*ϕ*s to the tumor site, they reported incorporating DTX and spherical 10 nm size Fe_3_O_4_ NPs within spherical PLGA NPs with a mean final diameter of 300 nm, followed by loading them into M*ϕ*s with incubation (**Figure** **4**a). Fluorescent imaging revealed uptake of NPs by the M*ϕ*s (Figure 4b). In the absence of an external magnetic field, the M*ϕ*s migrated toward tumors based on their natural tendency for tumor recruitment, while imposing a 10 mT m^−1^ magnetic field accelerated the migration of the M*ϕ*s to cancer cells. Figure 4c visualizes the magnetically facilitated migration of M*ϕ*s toward a tumor spheroid over the time course. The developed cell‐based microrobots also showed desirable antitumor activity on murine colorectal CT‐26 cancer cell line (Figure 4d). In another study by Zheng et al., tungsten oxide nanorods were synthesized and before being loaded into M*ϕ*s, the nanorods and a fluorescent dye were incorporated into PLGA NPs to protect the living M*ϕ*s from the rods’ spindle‐like bundles showing some surface spines.^[^ ^45e^ ^]^ The PLGA NPs were transferred to the M*ϕ*s with incubation and demonstrated negligible toxicity on the M*ϕ*s after uptake. Phagocytic ability and production of cytokines were also preserved. The presence of a fluorescent agent and light to heat conversion potential of the tungsten oxide facilitated image‐guided photothermal therapy of melanoma tumors in vivo under 808 nm laser irradiation.

One of the obstacles in cancer therapy is the hypoxia associated with the tumor environment. To circumvent this problem, in situ delivery of nitric oxide (NO) has been suggested as a promising strategy.^[^ ^46^ ^]^ To this end, a near‐infrared (NIR)‐sensitive manganese‐nitrosyl complex (photoNORM) as well as neodymium upconverting NP (Nd‐UCNP) for imaging purposes were loaded within PLGA NPs that were coated with immunoglobulin G (IgG) for better uptake by M*ϕ*s. PhotoNORM loading efficiency within PLGA was about 4.36 ± 0.66% and about 236 µg NPs were loaded within 10^6^ M*ϕ*. No significant toxicity associated with photoNORM‐UCNP‐PLGA particles was observed on the M*ϕ*s in the experimental condition. It was revealed that increasing NIR irradiation intensity would cause an obvious reduction in the tumor spheroid cells’ survival as a result of augmented NO release in the M*ϕ*s.^[^ ^45f^ ^]^

Silica‐based NPs are another class of carriers that have been extensively investigated for drug delivery purposes, mainly because of their large and tunable surface area as well as their high stability.^[^ ^47^ ^]^ These NPs have been utilized alone^[^ ^48^ ^]^ or in combination with other nanoparticulate systems for additional functionality.^[^ ^49^ ^]^ Zhang et al. reported a silica core–silica shell platform for postponing drug release from the M*ϕ*s, so that M*ϕ*s will have enough time to home into the cancer tissue, avoiding off‐target toxicities.^[^ ^48^ ^]^ For this purpose, silica shells with different thicknesses (12, 22, and 52 nm) were designed so that they could resist enzymes and oxidative conditions in the phagolysosomes of M*ϕ*s, where the NPs localize for a long period. DOX was encapsulated into the silica core with a diameter of 28.4 ± 3.4 nm. During a 2 h incubation period, the M*ϕ*s took up the majority of silica NPs via phagocytosis in a concentration‐dependent manner. Incubating M*ϕ*s with 20 µg mL^−1^ of NPs resulted in a 15.9 pg per cell DOX loading, cell viability of ≈70% after 12 h, and cell migration ability was maintained. Interestingly, analyzing the released cytokines from the M*ϕ*s showed phenotype change from M0 to M1 after NP uptake. Drug release studies from M*ϕ*s also revealed that not only was free DOX released by the cells, but also DOX‐containing Exos with a size of 50–150 nm were produced. The developed system revealed no significant pharmacokinetic divergence from that of pristine M*ϕ*s, implying no alteration in the M*ϕ*s’ tumor tropic properties after NP uptake. As a result, the tumor size was reduced and the animal survival was impressively increased, while no DOX‐associated cardiotoxicity and hematological abnormalities were observed. In another study, an albumin–PTX conjugate was incorporated into mesoporous silica NPs, followed by loading into M*ϕ*s to overcome poor drug accumulation within the tumor. It was revealed that incorporating albumin–PTX conjugate within mesoporous silica NPs could increase its uptake efficiency by M*ϕ*s. Transwell migration assay confirmed an enhanced migration ability of loaded M*ϕ*s toward breast cancer cells.^[^ ^49b^ ^]^

Among studies on NP‐loaded M*ϕ*s, many have deployed silica–gold hybrid NPs to combine silica's high drug loading efficiency and gold's potential for photothermal therapy and imaging. Both of these nanoparticulate systems can offer controlled release properties for M*ϕ*‐based systems.^[^ ^49c^ ^]^ Madsen et al. have reported that M*ϕ*s have a greater uptake efficiency for nonmodified silica core–gold nanoshells in comparison to the PEGylated counterpart during incubation in similar conditions. However, due to its merits, they continued the experiments with the PEGylated form, which consisted of a 120 nm diameter silica core and a 15 nm thick gold nanoshell (AuNS).^[^ ^49d^ ^]^ In the coculture of M*ϕ*s with the spheroid model of glioma, no tumor growth was observed following NIR irradiation. In a mouse glioma model, loaded M*ϕ*s were injected intracranially and treated with a laser beam. Compared to that of the control group, the tumor remnants in treated mice were significantly smaller and no post radiation tumor growth was observed during the study. However, the treatment protocol seemed to be associated with partial damage to neighboring tissue. In the next step, Madsen et al. tested the effect of PEGylated silica–gold nanoshell (S–GNS‐PEG)‐loaded M*ϕ*s on the efficacy of three cytotoxic agents (DOX, bleomycin, and cisplatin) against squamous cell carcinoma in vitro in the presence of laser irradiation for inducing hyperthermia.^[^ ^49f^ ^]^ M*ϕ*s had a 4% uptake efficiency for S–GNS‐PEG and transmission electron microscope (TEM) imaging revealed the extensive presence of NPs in cytoplasmic vacuoles of the M*ϕ*s. M*ϕ*s were cocultured with the tumor cells and each of the individual chemotherapeutic agents was added to the culture media before receiving irradiation. It was observed that combining photothermal therapy (PTT) provided by NP‐loaded M*ϕ*s with chemotherapy had synergistic antitumor effects. These NP‐loaded M*ϕ*s were also used by the same group for combined PTT and photodynamic therapy (PDT) of head and neck squamous cell carcinoma. They co‐incubated loaded M*ϕ*s with the tumor cell line, followed by adding a potent photosensitizer, Al(III) phthalocyanine chloride disulfonic acid (AlPcS2a) to the culture media.^[^ ^49e^ ^]^ Laser beams with the wavelengths of 810 and 670 nm were then irradiated for PTT and PDT purposes, respectively. Compared to the samples receiving single treatment, those receiving dual treatments revealed a synergistic antitumor effect of combined PTT and PDT. It also showed that the injection site can have a significant effect on the anticancer effect of the NP‐loaded M*ϕ*s. For example, after loading the silica core–gold shell NPs into the M*ϕ*s, they were injected peritumoral (in four tumor surrounding locations) or intratumoral (in the mid‐portion of tumor mass). NIR laser of 960 nm with irradiance of 1 W cm^−2^ and spot diameter of 1.2 cm was irradiated to tumor sites 48 h postinjection for 2 min. For the groups that received intratumoral injection of the M*ϕ*s, although fibrotic and dead cells were present mainly in the vicinity of the injection site, alive tumor cells were still present in other portions of tumor mass. In contrast, in those animals that received peritumoral injection of M*ϕ*s, the tumor was evenly eradicated and only fibrotic tissue was observed.^[^ ^49i^ ^]^

**Figure 5:**
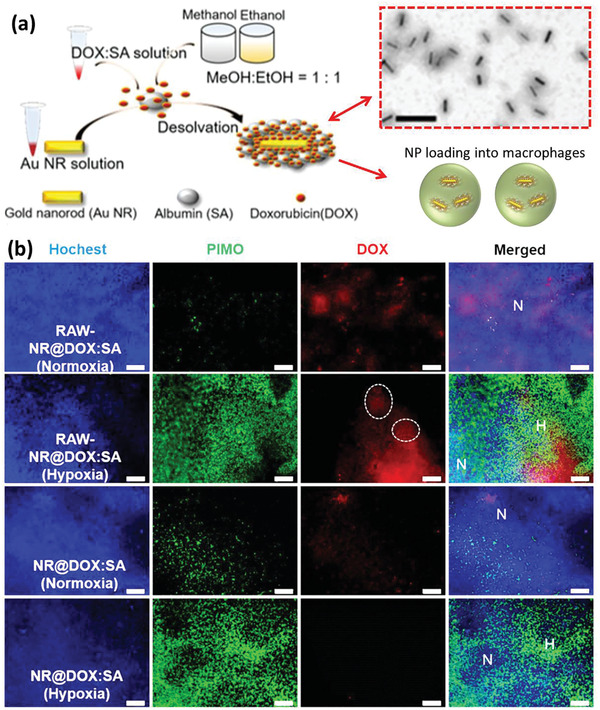
a) Preparation of core–shell NR@DOX:SA nanoplatform programmed for cell‐mediated drug delivery. Efficient controlled drug release was achieved through NIR light irradiation. The TEM image of NR@SAs shows monodisperse particles. Scale bar is 200 nm. b) Immunofluorescence staining of tumor sections after treatment by the therapeutic macrophage and pristine nanoparticles in different conditions. Hoechst was administered i.v. and stained nuclei in blue color and hypoxia areas were visualized by PIMO (green) marker. Normoxia region (N) referred to the areas in which blood flow and Hoechst staining (blue) was observed without any hypoxia signal (green), and vice versa for defining hypoxia region (H). The white circle indicates the accumulation of therapeutic macrophages in the hypoxic regions. Scale bar is 100 µm. Reproduced with permission.^[^ ^50^ ^]^ Copyright 2017, Ivyspring International Publisher.

Based on the merits of gold NPs, other platforms rather than silica‐based DDSs have also been combined with the gold NPs. Chiu et al. have reported using M*ϕ*s to carry gold nanorods (AuNRs) coated with DOX‐loaded serum albumin into the tumor (**Figure** **5**a).^[^ ^50^ ^]^ AuNRs with a length of 56.4 ± 3.5 and width of 11.9 ± 1.8 nm were covered with an even, dense layer of albumin to inhibit drug release before M*ϕ*s could home into the tumor site. NIR laser irradiation could trigger drug release from M*ϕ*s in vitro. Following intratumoral injection, NP‐loaded M*ϕ*s revealed long preservation in the tumor tissue in comparison with free DOX solution or pristine NPs. To evaluate the tumor migration ability of NP‐loaded M*ϕ*s, they were also administered intravenously and revealed a substantial accumulation in hypoxic regions (Figure 5b).

A relatively smaller albumin‐coated AuNR (core size: ≈30 nm length × 7 nm width) with a final zeta potential of −22 mV has also been used for PTT.^[^ ^51^ ^]^ The results of cellular uptake efficiency and cell toxicity demonstrated the superiority of these smaller nanorods as compared to bigger counterparts (≈56 nm length × 14 nm width), while their photothermal conversion efficiency was just the same. In a mouse model of liver cancer, intratumoral injection of small nanorod‐laden M*ϕ*s in the tumor center led to a better distribution in tumor tissue compared to pristine nanorods, which resulted in an even heat distribution through the tumor tissue. During irradiation, a higher temperature was recorded in the tissues that were treated with the NP‐loaded M*ϕ*s in comparison to the control groups. Summing up altogether, NP‐loaded M*ϕ*s culminated in almost 95% of tumor growth inhibition with no recurrence after 2 weeks, while in mice that received pristine nanorods, the tumor recurrence was recorded after 6 days. These albumin‐coated small AuNRs have recently been used for developing more complex M*ϕ*‐based DDSs. Nanorods with an average length of 29 nm, a width of 7 nm, and a zeta potential of −9.48 mV were conjugated with albumin. These particles were coloaded into M*ϕ*s with DOX‐encapsulated thermosensitive liposomes with an average diameter of 145 nm. Then the M*ϕ*s were evaluated for chemotaxis behavior toward a 3D breast cancer spheroid model in vitro. Its success demonstrated the potential of the developed system for the in vivo solid tumor targeting.^[^ ^52^ ^]^

Another sophisticated M*ϕ*‐based biomimetic system with the potential of being guided through a magnetic field, as well as facilitating PTT and controlled drug release in response to NIR laser has been developed by Nguyen et al.^[^ ^53^ ^]^ Superparamagnetic iron oxide NPs (SPIONs) of 11 nm diameter were loaded into PTX containing liposomes with a final average diameter of 148 nm. Using fluorescent labels, it was revealed that NPs were localized in the cytoplasm of the M*ϕ*s. Under the electromagnetic actuating system, NP‐loaded M*ϕ*s could move with an average velocity of 11 µm s^−1^, while the measure for the control pristine M*ϕ*s was only 0.4 µm s^−1^. A transwell migration assay also confirmed that the innate extravasation and tumor‐targeting properties of M*ϕ*s remained intact after being loaded with NPs. It was demonstrated that a controlled (accelerated) drug release pattern following a 10 min exposure to NIR light (808 nm) was achieved.

**Figure 6:**
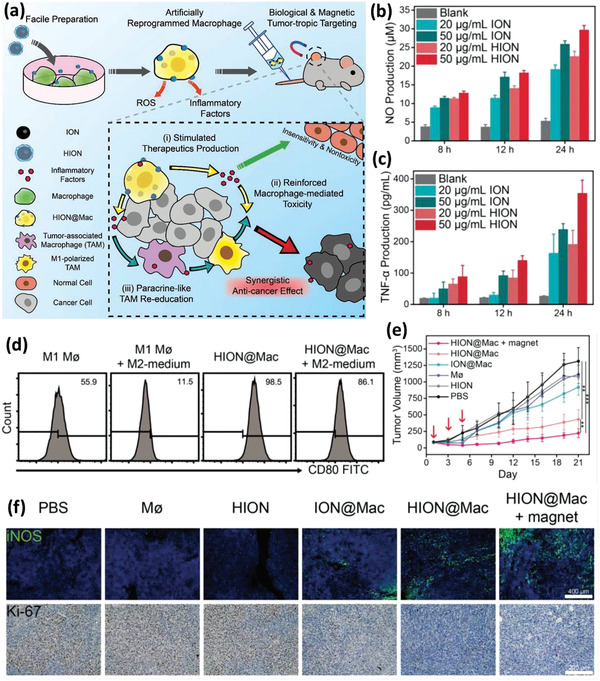
a–c) Schematic depiction of the artificially reprogrammed HION‐loaded M*ϕ*s (HION@Macs) and targeting of the tumor tissue through active chemotaxis and magnet guidance. Engineered HION@Macs induce more production of inflammatory cytokines (such as NO and TNF‐*α*), resulting in the in situ re‐education of M2 macrophages into proinflammatory M1 phenotype for cancer therapy. d) Flow cytometry analysis of CD80 expression in pristine M1 M*ϕ*s, M1 M*ϕ*s cultured in M2 medium for 24 h, HION@Macs, and HION@Macs after treatment with M2 medium for 24 h. M2 medium has a negligible effect on the phenotype transition from M1 to M2 in the HION@Macs. e) Tumor volume profile of animals for 21 days. Three injections of different formulations on the 1st day, 3rd day, and 5th day (designated by a red arrow) were conducted in tumor‐bearing BALB/c mice once tumor volume reached ≈80 mm^3^. The asterisks indicate the difference between the HION@Macs + magnet group, the HION@Macs group, and the PBS group. ^**^ *p* < 0.01; ^***^ *p* < 0.001. f) Representative immunofluorescence staining images for iNOS (green) and immunohistochemistry staining images for Ki‐67^+^ areas (gray dots) of tumor sections from different groups on the 7th day post‐treatment. Reproduced with permission.^[^ ^54^ ^]^ Copyright 2019, Wiley‐VCH.

Li et al. recently proposed another application in addition to the potential of inducing PTT, triggered drug release, and being magnetically guidable by iron oxide NPs (IONs).^[^ ^54^ ^]^ Based on the fact that intracellular presence of iron ions can induce a tumor necrosis factor alpha (TNF‐*α*) associated proinflammatory immune response in the M*ϕ*s, they attempted to educate tumor‐associated M*ϕ*s (TAMs) with ex vivo engineered M*ϕ*s to activate a strong antitumor response (**Figure** **6**a). For this purpose, IONs were coated with hyaluronic acid (HIONs) for facilitating their uptake by M*ϕ*s via hyaluronate–CD44 interactions, and further the HIONs were incubated with M*ϕ*s for 4 h. It was revealed that hyaluronic coating could increase the uptake efficiency by up to twofold compared to nude IONs, while neither significantly affected the viability of the host M*ϕ*s after 24 h of incubation. The HION‐loaded cells produced relatively more ROS, NO, and TNF‐*α* because of the HIONs’ higher uptake efficiency and the subsequent higher iron NP content within the host cells (Figure 6b,c). Further, in vitro cytotoxicity tests demonstrated that M*ϕ*s could recognize and discriminate between normal and tumor cells. While coculture of the NP‐loaded M*ϕ*s with normal cells showed no alterations in the viability and activity of the normal cells, their coculture with tumor cells led to a strong tumoricidal and cytostatic–proapoptotic response in M*ϕ*s, and as expected, the superior results belonged to HION‐loaded M*ϕ*s when compared to ION‐containing cells. It was observed that M1 M*ϕ*s loaded with HIONs resisted the immunomodulatory effect of the tumor microenvironment, hardly changing their phenotype to protumoral M2 M*ϕ*s compared to pristine M1 M*ϕ*s (Figure 6d). The number of activated (CD80^+^) M1 M*ϕ*s loaded with HIONs during the experiment decreased slightly from 98.5% to 86.1%, whereas that of pristine M*ϕ*s abated sharply from 55.9% to 11.5%. In vivo studies in a mouse breast cancer model revealed that following the i.v. injection of NP‐loaded M*ϕ*s, they efficiently migrated to the tumor site. Nevertheless, under a magnetic field, the migration was accelerated and more efficient, leading to a lower M*ϕ* distribution in other organs. It was observed that strong antitumor and antimetastatic efficiency were achieved in 4T1 breast tumor‐bearing mice, as reflected in the tumor volume (Figure 6e). CD206 (a marker of M2 macrophage) immunofluorescence staining of the tumor tissues at the 7th day post different treatments showed that the HION‐loaded M*ϕ*s could effectively reverse M2 M*ϕ*s within tumors to M1 phenotype. In addition, inducible NO synthase (iNOS, a marker of M1 phenotype) immunofluorescence staining and immunohistochemical staining of Ki‐67 of the tumor tissue on the 7th day after treatments visualized; 1) the highest expression of the M1 phenotype in the tumor tissue of the mice treated with HION‐loaded M*ϕ*s and HION‐loaded M*ϕ*s+magnet, and 2) significant suppression of Ki‐67^+^ (cancer cell proliferation marker; brown areas) for HION‐loaded M*ϕ*s and HION‐loaded M*ϕ*s+magnet (Figure 6f).

In addition to the aforementioned commonly used NPs, other types of NPs have also been used for loading into M*ϕ*s.^[^ ^55^ ^]^ For example, a formulation of SN‐38, an irinotecan active metabolite with 1000 times higher potency, was developed to be loaded into M*ϕ*s for in vivo application.^[^ ^56^ ^]^ Short rod‐shape SN‐38 NPs (using no carriers) with an average size of 119.13 ± 4.57 nm and a zeta potential of 32.2 ± 1.73 mV were synthesized, fluorescently labeled, and incubated with M*ϕ*s for 2 h. The uptake efficiency into M*ϕ*s had a positive relationship with the NP concentration, and in the test conditions 14.16 ± 3.45 × 10^−9^ g per cell of NPs were loaded into M*ϕ*s. The host cellsʼ viability and migration potential were not extensively affected over this short period. In vivo studies revealed that 8 h following i.v. injection of NP‐loaded M*ϕ*s, most of the M*ϕ*s were recruited in the tumor site with minor distribution in other healthy organs. Furthermore, it was observed that compared to the control groups, tumor growth was significantly inhibited by NP‐loaded M*ϕ*s and the tumor weight was reduced.

### 2.3 Alive Monocytes for Drug Loading and Nanoengineering

**Figure 7:**
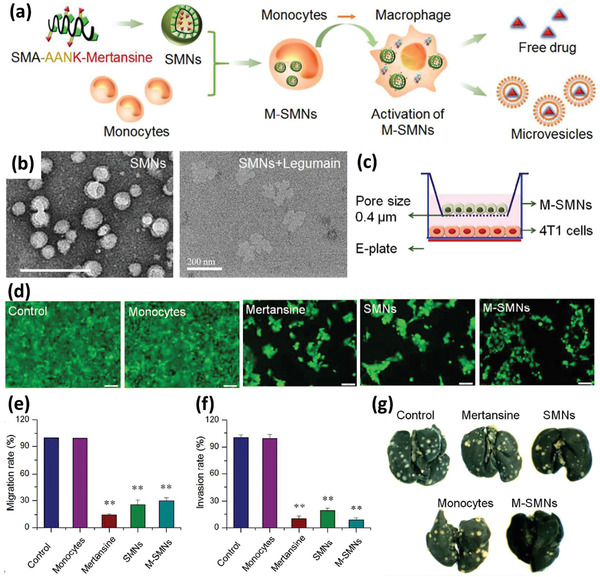
a) Schematic illustration of M‐SMN preparation and free drug or vesicle release after MC to M*ϕ* switching for antimetastatic purpose. Mertansine was first attached to the legumain sensitive peptide linker. Next, the conjugation of drug‐linker to the SMA polymer resulted in the formation of SMA‐AANK‐mertansine. b) TEM image of the developed SMNs in the absence (left) and the presence (right) of legumain. c) The schematic depiction of studying the inhibitory effect of M‐SMNs on the proliferation of 4T1 cancer cells. d) The inhibitory effect was visualized by Live/Dead assays at 48 h postincubation of 4T1 cells with MCs, mertansine (0.5 µg mL^−1^), SMNs (0.5 µg mL^−1^ of mertansine), and M‐SMNs (1 × 10^5^ cells per well). e,f) The migration and invasion activities of metastatic 4T1 cells were measured by transwell‐mediated assays. g) Formation of metastatic nodules (pale spots) in the lungs of mice treated with saline (control), free mertansine molecules, SMNs, MCs, and M‐SMNs. Adapted with permission.^[^ ^60^ ^]^ Copyright 2017, American Chemical Society.

MCs are another type of leukocyte with properties and functions very similar to those of DCs and M*ϕ*s. They are large phagocytic cells that can migrate from blood to the tissues and differentiate into DCs and M*ϕ*s. These qualities have drawn significant academic attention for developing cell‐based DDSs.^[^ ^57^ ^]^ It has been revealed that MCs prefer to uptake NPs with a size of 80–250 nm, a spherical shape and with a nonzero surface charge (preferably positive).^[^ ^58^ ^]^ However, optimum concentration of NPs should also be taken into account.^[^ ^38^, ^59^ ^]^ In an interesting study, a MC‐based system targeting lung metastasis of breast cancer was reported by taking advantage of bioresponsive legumain (lysosomal protease highly expressed in M*ϕ*s) activated NPs. To this end, the cytotoxic agent, mertansine, was conjugated to a block copolymer, poly(styrene‐*co*‐maleic anhydride) (SMA), via a legumain sensitive linker peptide, while being self‐assembled to legumain‐activated spherical NPs (SMNs). SMNs were then loaded into Ly6c^+^ inflammatory monocytes to form SMN‐laden monocytes (M‐SMNs) for the targeting of lung metastases of breast cancer. After being loaded to MCs, because of legumain absence and inhibited drug release, MCs remained viable until differentiation to M*ϕ*s, when the cytotoxic agent kills the host cells and is then released into the surrounding environment in the form of free mertansine molecules or nanovesicles containing the drug as a result of legumain activity (**Figure** **7**a). The TEM images of the M‐SMNs and M‐SMNs+legumain is shown in Figure 7b. It was also observed that drug release in the physiologic pH and in the absence of legumain in pH5.5 is negligible, while it significantly increases with the raise in legumain concentration. In a transwell coculture study, the inhibitory effects of M‐SMNs on the proliferation of 4T1 breast cancer cells was studied and showed the proliferation of tumor cells was reduced significantly (Figure 7c,d). A transwell migration and invasion test also revealed that these two characteristics could be extensively hampered by both the pristine NPs and NP‐loaded MCs (Figure 7e,f). For cell migration assay, 1 × 10^5^ of 4T1 cells were dispersed in 100 µL of fetal bovine serum (FBS)‐free RPMI 1640 media and added to the upper chambers of inserts (24‐well insert, pore size, 8.0 µm). For the invasion assays, 20 µL of Matrigel matrix (BD, USA) was used to coat the transwell‐membrane of inserts before adding 2.5 × 10^5^ of 4T1 cells dispersed in 100 µL of FBS‐free media to the top chambers of inserts. Then, 600 µL of complete RPMI 1640 media with 1 × 10^5^ of M‐SMNs, 1 × 10^5^ of MCs, and equivalent dose of mertansine and SMNs were added to the lower chambers of 24‐well plate. After 24 h of incubation, the migrated or invaded cells across the transwells were stained with violet crystal and counted to calculate the inhibitory rate. 4T1 cells without any treatment were considered as negative control. In addition to desirable inhibition of migration and invasion, the M‐SMNs revealed significant accumulation, penetration, and recruitment in tumor lesions and obviously inhibited lung metastasis compared to the other groups in vivo as can be seen in Figure 7g, paying attention to the number of metastatic nodules in mice lungs after staining using India ink.^[^ ^60^ ^]^

A pH‐responsive NP carrying PTX and incorporated within MCs is also reported to target breast cancer and its associated lung metastasis.^[^ ^61^ ^]^ Polyethylene glycol‐*block*‐poly [(1,4‐butanediol)‐diacrylate‐*β*‐*N*,*N*‐diisopropylethyl‐enediamine], as a hydrophilic pH‐responsive polymer, was exploited for fabricating micelles with a size of 26.74 ± 6.35 nm. NPs were then loaded to MCs and drug loading was estimated to be 1.6 µg per 10^4^ cells. Drug release was very low in acidic conditions, implying that MCs could postpone drug release until MCs are recruited at the tumor site. The results from the coculturing of tumor cells and NP‐loaded MCs revealed a 10.1‐fold decrease in the half‐maximal inhibitory concentration (IC50) of the NP‐loaded MCs compared to the free PTX molecules. Furthermore, the biodistribution and pharmacokinetic studies demonstrated that accumulation of the drug in the tumor is more efficiently achieved by NP‐loaded MCs in comparison to the free drug molecules or pristine NPs and a prolonged presence in circulation was also observed. In vivo evaluations confirmed a tumor inhibition rate of 96.8% and 50.4% for the NP‐loaded MCs and pristine NPs, respectively. Lung metastasis was significantly inhibited by the NP‐loaded MCs, possibly attributed to their distribution in the lung.

In another study, MCs have been harnessed to deliver micrometer‐size poly(acrylic acid‐*co*‐distearin acrylate) bubbles, containing citric acid‐coated SPIONs, oxygen, and chlorine e6 photosensitizer, to prostate adenocarcinoma site for combined PTT–PDT.^[^ ^62^ ^]^ In the first step, it was revealed that microvesicles (MVs) were efficiently taken up by MCs (the chlorine e6 and SPION content were 7.92 ± 0.11 and 17.3 ± 4.6 µg, respectively, in 2 × 10^6^ cells), revealing neither significant intracellular oxygen leakage in 48 h nor toxicity toward the MCs in the absence of a magnetic field. However, the MC's migration ability diminished by about 25%. In vivo studies demonstrated that whereas the MVs were placed more rapidly within the tumor environment, the MV containing MCs had a higher accumulation over a longer period within the tumor site. The same research group has also developed echogenic nanobubbles based on poly(acrylic acid‐*co*‐distearin acrylate) (different in distearin content as compared to the previous study).^[^ ^63^ ^]^ They used perfluoropentane and DOX for loading into MCs, to target prostate adenocarcinoma. Since the DOX was retained within the nanovesicles, it had a minimal toxic effect on the host cell compared to the free DOX. In a transwell migration test, it was observed that nanovesicle‐carrying MCs lost their migration capability by about 50%, while pretreatment of MCs with tumor conditioned medium that received *γ* irradiation improved this impaired migration ability. In vivo studies revealed that although accumulation of NP‐containing MCs in tumor site happened more slowly, its efficiency was far greater than that of the unloaded nanovesicles, because the majority of vesicles accumulated in the liver. It was demonstrated that the nanovesicle‐carrying MCs could be recruited in both hypoxic and normal regions, while unloaded NPs had relatively limited penetration in hypoxic sites.

As mentioned, the compromised migrating ability of MCs following NP uptake may result in undesirable tumor homing and antitumor results. To solve this problem, the effect of radiation on the extent of MC infiltration into the prostate adenocarcinoma was investigated.^[^ ^64^ ^]^ Vesicles based on poly(acrylic acid‐*co*‐distearin acrylate) were first fluorescently labeled before i.v. injection in to mice bearing a prostate tumor. These mice also received a single 25 Gy dose of X‐ray radiation 1, 4, or 7 days before the injection. One day after vesicle injection, fluorescence was detected in the edge of the tumor, showing no significant difference between X‐ray radiated and nonirradiated mice. Nevertheless, 1 or 2 weeks after vesicle injection, there was a distinguishable difference between the number of fluorescent cells in irradiated tumors compared to nonirradiated ones. These results presented the impact of X‐ray radiation on the long‐term homing of the particles within the tumor tissue. Further in vitro migration tests revealed that the migrating ability of MCs increases in the tumor conditioned medium that had been irradiated due to the factors present in the tumor microenvironment.

### 2.4 Alive Neutrophils for Drug Loading and Nanoengineering

NEs are the most abundant leukocytes in the circulation and have an integral role in the innate immune system. Moreover, they are usually the first immune cells that arrive at the inflammation site, before M*ϕ*s, MCs, and lymphocytes. These reasons give NEs an advantage over the other types of immune cells for drug carrier use.^[^ ^65^ ^]^ In the case of inflammation, endothelial cells capture activated NEs, followed by NE rolling, adhesion, crawling, and finally transmigration from the blood vessels to the inflamed tissue.^[^ ^66^ ^]^ Specifically, chemokines and cytokines produced by tumor cells act as chemotactic signals for NEs. After recruitment in the tumor site, NEs can express neutrophils with N1 phenotype (antitumor activity) (N1) or neutrophils with N2 phenotype (protumor activity) (N2) phenotype, with the antitumor and protumor functions, respectively.^[^ ^67^ ^]^ Due to the extensive crosstalk between NEs and tumor cells, they have been subjected to expansive investigations for drug delivery purposes, but since NEs are very sensitive to cargo chemotherapeutic agents, NPs have been harnessed for drug loading to these cells. It is reported that NEs take up smaller NPs (30–50 nm) through receptor‐mediated endocytosis very efficiently, while a shorter uptake time was reported for NPs with 50 nm diameter compared to those with smaller size.^[^ ^68^ ^]^ There are also conflicting shreds of evidence on the preferred NP shape to be uptaken by NEs,^[^ ^69^ ^]^ requiring more investigations that compare NPs with different morphology but same composition, surface chemistry, and charge. It has been revealed that NEs’ surfaces hold negative charge and hence positively charged NPs have a greater uptake efficiency; however, the tolerable charge is limited and highly positive NPs would damage the cell membrane.^[^ ^70^ ^]^ A variety of surface modifications have been reported to improve the uptake of NPs by NEs, including surface decoration with *cl* PGP‐PEG‐DGL/CAT‐Aco (due to the high bonding affinity to CXCR2 on NEs),^[^ ^71^ ^]^ denatured albumin,^[^ ^72^ ^]^ and TA99 antibodies (Abs).^[^ ^73^ ^]^ For developing efficient delivery systems, improving the migration ability, the tumor homing, and drug release properties of NE‐based DDSs, as well as engineering the tumor microenvironment through external stimuli like irradiation are also of a great importance.^[^ ^74^ ^]^ A study showed that a proper radiation dose (5 Gy) to the tumor site induced the upregulation of inflammatory cytokines in the tumor tissue and subsequent attraction of NEs to accumulate within the inflammation site.^[^ ^75^ ^]^ Although the increased dose of radiation resulted in higher expression of these cytokines and further migration of NEs, the potential side effects limited the radiation dose. Therefore, direct administration of NEs was introduced as an alternative. In this context, the possible toxicity of NEs as drug carrier was first investigated to check their safety when they are overloaded in the blood. Human NEs were intravenously injected to mice in five succeeding days at different doses. As a result, a dose‐dependent blood toxicity (abated cell count) was observed, while the organs were not damaged. Concludingly, human NEs distinguished to be safe only when the administered dose was below 3 × 10^6^ per mouse. Next, Abraxane (albumin‐conjugated PTX) was incubated with NEs and was taken up with a concentration of 18 µg per 10^6^ cells in the optimized condition. PTX amount in the cells remained stable for up to 4 h in the phosphate buffered saline (PBS) and 6 h in plasma. These results suggested that NP‐loaded NEs must be administered rapidly after preparation, so that the NEs would have enough time to reach the tumor site before they release their cargo. The NP‐loaded NEs demonstrated physiological functions including phagocytosis, transendothelial migration, and chemotactic ability, as well as superoxide generation and extracellular trap formation with a similar effeciency to the pristine NEs. The in vitro cytotoxicity of NP‐loaded NEs on gastric tumor cells was also significant and comparable to the Abraxane itself.

**Figure 8:**
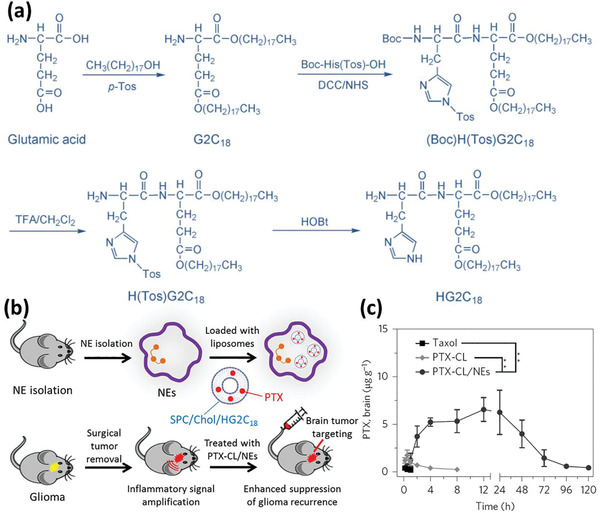
Neutrophils carrying PTX‐loaded CL for suppressing postoperative malignant glioma recurrence. a) Synthesis of the cationic lipid HG2C_18_ for fabricating CLs. b) Schematic depiction of PTX‐CL/NEs formation and its effect on the suppression of postoperative glioma recurrence in mice. Surgical resection of a glioblastoma induces amplified inflammatory signals in the brain, which allows PTX‐CL/NEs to target brain tumors and release PTX to suppress the recurrence of glioma. SPC, Chol, and HG2C_18_ refer to soy phosphatidylcholine, cholesterol, and 1,5‐dioctadecyl‐*N*‐histidyl‐l‐glutamate, respectively. c) Quantification of the PTX distribution in the brain of the surgically treated G422‐bearing mice after i.v. administration of different PTX formulations at a PTX dosage of 5 mg kg^−1^ over time. Data are shown as mean ± s.d. of three independent experiments. ^**^ *p* < 0.01 and ^***^ *p* < 0.001 (two‐tailed Student's *t*‐test). ** represents *p* < 0.01. Reproduced with permission.^[^ ^76^ ^]^ Copyright 2017, Springer Nature.

Xue et al. have also attempted to suppress postoperative glioma recurrence utilizing NP‐loaded NEs.^[^ ^76^ ^]^ First, they synthesized the cationic lipid 1,5‐dioctadecyl‐*N*‐histidyl‐l‐glutamate (HG2C_18_) (**Figure** **8**a), which along with soy phosphatidylcholine (SPC) and cholesterol (Chol) formed the cationic liposomes (CLs). The PTX‐loaded liposomes (PTX‐CLs) had an average size of 100 nm and were able to provide sustained release of the encapsulated drug.

After incubation with the NEs, PTX‐CL was efficiently taken up by the cells to form PTX‐CL/NEs without any significant damage to the physiological functions and morphology of NEs compared to the pristine NEs. The final concentration of PTX in the formulation was measured to be 18 µg per 10^6^ cells and the final formulation was used for the treatment of glioblastoma (Figure 8b). In the physiological condition and while receiving chemotaxis signals, the NEs revealed no significant drug leakage; however, in simulated inflammatory condition, a burst drug release was observed. This observation was desirable for the targeted restriction of glioblastoma recurrence after surgery, which results in an increased level of inflammatory cytokines in the tumor removal site. It was shown that 88% of the PTX was released from the NEs in the form of liposome‐loaded rather than free molecules. In a BBB in vitro model and under a simulated inflammatory condition, NEs showed significant transendothelial passage via opening the tight‐junctions, while they could not penetrate the endothelium in the absence of chemotactic signals. As soon as the NPs were released from the NEs in response to the inflammation, they were taken up by tumor cells, followed by releasing the PTX therein and leading to a significant antitumor effect. Finally, a mouse model of glioma surgical resection was developed. Tumor recurrence was observed within 2 days postoperation in the control group, resulting in animal death in less than 22 days. However, i.v. injection of NP‐loaded NEs following the operation revealed promising results. Due to the increased production of inflammatory chemokines and cytokines in the surgical site, PTX‐CL/NEs were efficiently recruited and the amount of PTX was substantially increased in the brain of animals when compared to the commercial Taxol or PTX‐CL (Figure 8c). The PTX‐CL/NEs even colocalized with the tumor cells infiltrating the normal brain parenchyma, resulting in significantly improved animal survival. Nevertheless, several tumor islands were still detectable during 4 months of follow‐up, suggesting that the treatment protocol did not completely cure the complication.

In another endeavor for targeted therapy of glioma after surgical resection, mesoporous silica NPs with an average diameter of 75.3 nm were used to encapsulate Fe_3_O_4_ NPs and DOX, followed by 1 h incubation with NEs.^[^ ^77^ ^]^ NPs were efficiently taken up by NEs, revealing a capacity of 4.97 × 10^−5^ and 1.62 × 10^−5^ µg per cell for DOX and Fe_3_O_4_ NPs, respectively. As the phagosomes’ pH is slightly acidic, the release of iron oxide NPs and DOX at pH 5 was studied and no significant Fe_3_O_4_ NPs release was detected; however, DOX liberation was confirmed by changing the pH from the physiological to acidic condition. In vitro drug release in the presence of glioma tumor cells also demonstrated that DOX containing NPs were mostly released via NEs’ external traps and were further taken up by tumor cells, while NEs’ Exo carried only a few DOX molecules. In vivo studies on a mouse model of glioma resection revealed an elevated level of inflammatory cytokines and chemokines postsurgery, increasing the NEs’ migration and homing in the surgical site, which could be easily tracked through magnetic resonance imaging (MRI). Biodistribution pattern also illustrated the superior accumulation of the drug and diagnostic agents carried by NEs in the brain and less off‐target presence compared to the cell‐free formulation of NPs. Histological investigations revealed that NP‐loaded NEs not only directly affected the tumor cells, but also induce apoptosis in tumor‐associated M*ϕ*s supporting tumor development and progression. Altogether, the NP‐loaded NEs remarkably suppressed and delayed postsurgical glioma relapse and significantly increased animal survival.

Another study reported developing an intriguing strategy to improve the uptake efficiency of mesoporous silica NPs by NEs. First, the researchers extracted *Escherichia coli* membrane and fabricated vesicles using a miniextruder with 50 nm pore size, and then cloaked the DOX coated mesoporous silica NPs with the fabricated membrane vesicles. The final particles had an average diameter of 88.23 nm and a mean zeta potential of −15.3 mV. It was observed that the uptake efficiency of NPs augmented from 32.58% for noncamouflaged particles to 97.36% for the *E. coli* membrane camouflaged ones. Although the NPs showed slight cytotoxicity toward the host cells and NEs’ viability was diminished, their migration and chemotactic ability remained significantly comparable to those of pristine NEs.^[^ ^78^ ^]^

In an interesting study, posaconazole was loaded into the HL‐60 leukemia cells. They were differentiated to a NE phenotype to evaluate the efficacy of this system in the NE‐based drug delivery system to treat invasive pulmonary aspergillosis. The cells took up posaconazole, while the host cells’ viability and chemotaxis ability did not alter significantly. In a coculture of drug‐loaded NEs and fungal cells, the NEs could effectively transfer their payload to the *Aspergillus fumigatus* hyphae in a time‐dependent manner and an intensified antifungal activity was observed. In vivo studies on a neutropenic mouse model of invasive pulmonary aspergillosis confirmed that in about one‐third of the infected models no fungal lesions were detected after treatment with the drug‐loaded NEs, while all animals in the nontreated control group revealed extensive lesions.^[^ ^79^ ^]^

### 2.5 Other Alive Immune Cells for Drug Loading and Nanoengineering

TCs, although being less investigated for drug delivery purposes, still have advantages that make them worthwhile for this purpose.^[^ ^80^ ^]^ These merits include their specificity and induction of long‐term immune responses, while they are one of the most important effector cells against tumors, and engineering them could intensify their innate antitumor efficiency.^[^ ^81^ ^]^ To harness these qualities, D'Elios et al. have recently developed a theranostic platform based on TCs for brain drug delivery to treat multiple sclerosis (MS).^[^ ^82^ ^]^ NPs based on poly(glycolide‐*co*‐lactide)‐poly(ethylene glycol)‐COOH carrying small magnetite (30 nm in diameter), with a final hydrodynamic diameter of 44.1 ± 1.6 nm, a PdI of 0.17, and a zeta potential of −41 mV, were loaded into the Myelin Oligodendrocyte Glycoprotein 35–55 (MOG 35–55)‐specific TCs via a 2 h incubation period. NPs did not affect the TCs morphology, proliferating ability, and viability; however, compared to the pristine TCs, the NP‐loaded cells revealed a decreased level of IL‐17 and IFN‐*γ* production. They also expressed CD25 and CD62L surface markers equal to those of activated TCs. In vitro release studies showed that TCs released about half of their iron oxide NP content in the first 24 h, while about 60% of the PGLA NPs remain in the cell within 72 h. In the next step, 48 h following the i.v. injection of the NP‐loaded TCs to naïve mice with intact BBB, migration of TCs to specific brain regions was observed. In vivo experiments on a mouse model of MS (experimental autoimmune encephalomyelitis) showed that NP‐loaded TCs migrated to demyelinated lesions in the spinal cord after successful BBB passage. Another study conducted by Mühlberger et al. further evaluated different coatings on SPIONs to optimize their uptake efficiency by the TCs as well as their toxicity on the carrier cells, to develop magnetically guidable cells for cancer therapy.^[^ ^83^ ^]^ First, SPIONs were coated with lauric acid, although because of their severe toxicity, they were furthered cloaked with a layer of bovine serum albumin that led to a diminished uptake efficiency by TCs. To replenish the uptake efficiency, the albumin coating was followed by amination. In vitro tests confirmed that magnetized cells could migrate toward a magnet and the number of migrating cells and their velocity depended on their SPION content. Nevertheless, the homogenous distribution of SPIONs between TCs was not achievable due to the highly proliferative nature of the cells.

Due to the difficulties in the purification of a single type of cells for drug delivery purposes, a mixed population of immune cells has also been utilized. Schiariti et al. reported extracting fibronectin‐inherent mononuclear cells, i.e., monocytic‐dendritic cell classes, from the blood of healthy subjects and patients with glioblastoma. They further loaded the cells with PTX during a 24 h incubation.^[^ ^84^ ^]^ The PTX was localized in isolated intracellular compartments and induced a morphological transformation in cells from elongated to a spherical shape, while the hosts’ viability did not change dramatically. Nevertheless, the cells completely lost their ability to proliferate, expressed diminished levels of CD146 and VEGF receptor 2, and revealed less migrating ability toward tumor environment compared to the pristine cells. As expected, the antitumor activity of the drug‐loaded cells was dependent on the drug concentration the cells were primed with. Cells from patient subjects were found to have a superior antitumor activity compared to the cells from healthy subjects. Moreover, the drug‐loaded cells showed intensified antitumor response in comparison with pristine cells in 2D and 3D in vitro tumor models.

In general, the idea of a bionic superhuman is no longer science fiction. Scientists and engineers are collaborating to improve every aspect of the human body's functions for maintaining and idealizing its healthy state or treating different diseases. In this regard, engineering alive immune cells to develop highly effective and safe DDSs has been one of the hot trends in biomedical engineering.^[^ ^4b^ ^]^ Harnessing the natural capabilities of these cells, including their mobility and ability to patrol around the whole body, migration ability in response to specific signals, competence to pass through biological barriers, homing in the inflammation sites, recognizing and destroying foreign invaders and tumors, as well as their biocompatibility, manipulability, and high cargo intake capacity, have made them promising platforms for curing hard to treat diseases. Inserting antigens, drugs, diagnostics, and any material that provides more control over the cell functions (e.g., those that allow guiding the cells to a specific target like magnetic NPs and on‐command controlled drug release like core–shell silica NPs) within the diverse immune cells has been widely practiced.^[^ ^85^ ^]^ However, the immune cell‐based therapies are still in their infancy and many challenges remain to be addressed before their coming to the patients’ bedsides. Some of these challenges concern the inconsistent drug uptake by the cells, the effect of cell source on its effect, safety issues regarding their adverse inflammatory responses, their manufacturing scale‐up, developing authentic protocols for the quality control of the products, and the storage, distribution, and retail of the final product. The last but not least important issue to notice about the alive cell‐based therapies is that, despite the astronomical costs of developing such DDSs, it is still worthwhile to invest in these products looking at the shocking economical burden of currently uncontrollable diseases like cancers and CNS disorders.^[^ ^85^, ^86^ ^]^

## 3 Surface Engineered Immune Cells

The emerging context of cell therapy has focused on the application of cells as therapeutics, therapeutic carriers, or even both. In this regard, the cell surface, dictating every single interaction of the cell with its environment, is of great importance. The engineering surface of the immune cells provides more control over their microenvironment sensing, adhesion, migration, navigation, targeting, recruitment, communication, uptake, and release characteristics.^[^ ^87^ ^]^ To this end, various genetic and nongenetic engineering approaches have been developed to alter the physicochemical properties of the cell surface. Genetic engineering has been applied to induce expression of specific receptors, including those for cell surface tumor antigens, such as chimeric antigen receptor (CAR), synthetic notch (SynNotch) receptors, and cytokine receptors.^[^ ^88^ ^]^ Nongenetic approaches include inserting specific molecules or particles on the cell surface through noncovalent binding (e.g., electrostatic interaction, lipids with or without hydrophilic heads being anchored on the cell membrane, receptor–ligand interactions, and antibody–antigen interactions), covalent bindings (e.g., biotin–avidin interaction, *N*‐hydroxy succinimide (NHS) esters–maleimides coupling, and click chemistry reactions like azide–alkyne conjugation), or even membranous fusion with engineered liposomes.^[^ ^14^, ^37^, ^87^, ^88^, ^89^ ^]^ The following sections will focus on surface‐engineered immune cells and their advantages in preclinical and clinical subsets.

### 3.1 Drug Delivery by Surface Engineered Macrophages

Based on their extensive crosstalk with cancer cells, M*ϕ*s are very good candidates for delivering therapeutics and diagnostics to the tumor site. Although, their sensitivity to immunosuppressive signals in the tumor microenvironment and the possibility of transforming to the tumor‐associated phenotype has cast doubts on their application for this purpose.^[^ ^88^ ^]^ To address this problem, a method for surface engineering of M*ϕ*s has been proposed via the blockage of one of the suppressive signals.^[^ ^90^ ^]^ Previously, it was reported that tumor cells circumvent being phagocytosed by M*ϕ*s via various mechanisms, including presenting CD47, which interacts with signal regulatory protein a (SIRP*α*) receptors on M*ϕ*s and is recognized as the body's own and normal cells.^[^ ^91^ ^]^ Taking this into account, highly phagocytic bone‐marrow‐derived M*ϕ*s were targeted to lung carcinoma cells through specific Abs, while their SIRP*α* receptors were blocked. The engineered cells were injected intravenously to mice bearing large solid tumors in their lungs and then sacrificed after 3 days. Histological investigations revealed that M*ϕ*s were recruited in the tumor site and significantly phagocytosed tumor cells compared to nonengineered M*ϕ*s and tumor‐associated M*ϕ*s. Additionally, ex vivo studies on 3D tumor models confirmed an obvious tumor shrinkage following the application of engineered M*ϕ*s. However, it was observed that after 2–3 days of homing into the tumor microenvironment, the engineered M*ϕ*s differentiated into the tumor‐associated phenotype expressing high levels of SIRP*α* receptors and tumor regression stopped.^[^ ^90^ ^]^

To improve the targeting properties of M*ϕ*s for the tumor cells, cell surface decoration with nucleic acid aptamers was proposed. Thiol groups of Sgc8 aptamer (with affinity to protein tyrosine kinase 7 (PTK7), a membrane protein overexpressed on the cancer cell surface), reacted with methacryloyl‐modified *N*‐acetyl mannosamine analogs that were conjugated to the sialic acids on the M*ϕ*s surface. The surface‐engineered M*ϕ*s were incubated with human lymphoblasts as a cancer cell model. It was observed that after 30 min of gentle shaking, engineered M*ϕ*s were attached to the lymphoblasts, while nonengineered M*ϕ*s did not. It was observed that engineered M*ϕ*s’ attachment to target cells increased by extending the incubation time.^[^ ^92^ ^]^ The engineered M*ϕ*s were then co‐incubated with lymphoblasts, being pretreated with DOX for apoptosis induction, and it was revealed that they could capture both dead and alive lymphoblasts. Analyzing the inflammatory response by engineered M*ϕ*s, it was observed that surface decoration with the aptamers did not activate the M*ϕ*s and no alteration in the level of cytokine secretion was demonstrated. Nevertheless, in the presence of dead lymphoblast, expression of MHC I and II, as well as the release of cytokines was significantly higher compared to nonengineered M*ϕ*s. No change in phenotype transformation pattern of engineered cells was observed and both M1 and M2 cells were present in the coculture media with the lymphoblasts. The results suggested that the application of anticancer chemotherapeutics along with the developed surface‐engineered M*ϕ*s can improve the cancer treatment outcomes.^[^ ^93^ ^]^

**Figure 9:**
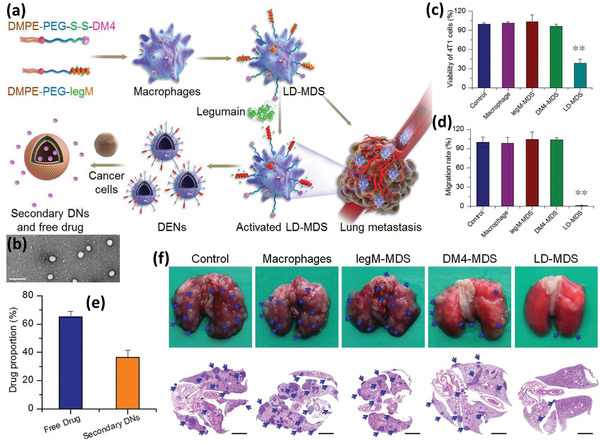
Surface‐engineered M*ϕ*s for targeting of lung metastasis. a) Fabrication of M*ϕ*s and their transformation to drug‐loaded vesicles in the presence of legumain in the tumor site. b) Field emission‐TEM image of the secondary drug‐containing vesicles from tumor cells treated with the drug‐loaded Exo‐like nanovesicles. c,d) The superior effect of the engineered M*ϕ*s in reducing tumor cells’ viability and migrating ability in vitro. e) Percent of the drug released in the form of free molecules or entrapped in the secondary vesicles in the supernatant of the tumor cells treated with the drug‐loaded Exo‐like nanovesicles. f) Typical imaging and histological examination of lung tissues of the animal subjects that received each of the test or control groups (metastatic nodules are pointed out). Adapted with permission.^[^ ^95b^ ^]^ Copyright 2018, American Chemical Society.

Responsive surface engineered M*ϕ*‐based platforms for efficiently delivering an anticancer agent to the tumor cells or working by itself as triggered immune cells have been employed as a promising strategy for the eradication of tumor cells.^[^ ^94^ ^]^ It is known that legumain is highly expressed in the tumor tissue. Hence, legumain‐specific propeptide melittin (legM) was conjugated to 1,2‐dimyristoyl‐*sn*‐glycero‐3‐phosphoethanolamine‐*N*‐methoxy (polyethylene glycol) (DMPE‐PEG) and then anchored on the M*ϕ*’s cell membrane (M*ϕ*CM). The same linker was also used to attach the redox‐sensitive prodrug of soravtansine (DM4) on the cell surface and produce a legM‐ and DM4‐loaded macrophage‐based delivery system (LD‐MDS) as shown in **Figure** **9**a. In vitro studies revealed that in response to the high doses of legumain present in the tumor site, the activated melittin would damage the host M*ϕ*s, leading to the formation of DM4‐loaded Exo‐like nanovesicles (DENs), while in a low concentration of legumain (simulating circulation condition), the M*ϕ*s remained viable. Legumain protease can specifically recognize a substrate peptide (NH_2_‐alanine–alanine–asparagine‐COOH, AAN) and cleave the bond at the COOH terminus of asparagine. This effect allows the legM peptide in LD‐MDS to transform into active melittin, which facilitated the transformation of LD‐MDS into DENs for antimetastasis therapy. The DENs would be able to efficiently penetrate into metastatic 4T1 cancer cells and induce remarkable cell death. Next, the damaged 4T1 cells could release secondary DENs (Figure 9b) and free drug molecules to kill neighboring cancer cells by transporting DM4 to other lung metastasis sites. It was observed that drug‐containing nanovesicles generated as a result of M*ϕ* damage in response to a high dose of legumain were efficiently taken up by cancer cells, and not only induced significant tumor cell killing (Figure 9c), but also inhibited proliferation and migration (Figure 9d) of the cancer cells considerably. Following the tumor cell damage, the DM4 was released in the tumor milieu in the form of free molecules or within the secondary nanovesicles (Figure 9e) that could be taken up by the neighboring tumor cells. In vivo studies were further conducted on a mouse lung metastatic model of breast cancer. The engineered M*ϕ*s were injected via the tail vein and efficient tumor permeation of the M*ϕ*s, as well as formation of DM4‐containing nanovesicles in the tumor site was confirmed. Formation of metastatic nodules in the lung was also significantly suppressed, compared to the mice receiving nonengineered M*ϕ*s or M*ϕ*s only carrying DM4 (Figure 9f). The results ultimately suggested that the developed approach for engineering M*ϕ*s hold great potential to improve the delivery of antitumor agents to the tumor site and inhibit the metastasis.^[^ ^95^ ^]^ In addition to the treatment of metastasis, surface engineered alive M*ϕ*s has also been proposed for brain drug delivery^[^ ^96^ ^]^ and tumor cell tracking via attaching fluorescent probes to the M*ϕ*s’ surface.^[^ ^97^ ^]^ Outcomes of these studies have shed light on the potential of surface modifications on the M*ϕ*s to treat various diseases.

### 3.2 Surface Engineering of T Cells

Based on their tumor tropism, specific targeting ability, and cytotoxicity, TCs have been in the center of attention, especially for cancer cell‐therapy, and therefore, many efforts have been made to improve the function of these cells for therapeutic purposes.^[^ ^98^ ^]^ They have undergone various surface modifications, from being attached to specific therapeutics or even NP containing payloads, to being genetically engineered to express specific molecules, as well as being subjected to ligand modification for targeting specific types of cells.^[^ ^99^ ^]^ For example, to improve cancer therapy in Epstein–Barr virus (EBV)‐associated tumors, Prussian blue NPs (PBNPs) were anchored on the surface of cytotoxic TCs for PTT.^[^ ^100^ ^]^ The surface of an EBV‐specific TC cell was biotinylated and conjugated with the avidin‐coated PBNPs to form NP‐conjugated TCs (TC–NP) with the diameter and zeta potential of about 80–90 nm and −40 mV, respectively. Further, it was confirmed that TC–NP conjugation changed neither the TCs’ phenotype, proliferation and cytokine production ability, nor the substantial potential of the PBNPs in NIR absorption and heat production. The TC–NP system was cocultured with model cells carrying EBV‐specific peptides for 4–8 h and then irradiated with an 808 nm NIR laser, which resulted in a significant decrease in the target cells’ viability.

In another advanced study, Wayteck et al. developed genetically engineered TCs expressing the OVA receptor, with the liposomes attached to their surface via a redox‐responsive bond, for delivering small interfering ribonucleic acid (siRNA) to the intracellular compartment of the tumor cells.^[^ ^101^ ^]^ Optimized liposome formulation was incubated with the naïve and activated TCs, and it was observed that the activated cells placed more liposomes on their surface compared to the naïve ones. It was also confirmed that in the presence of reducing agents like glutathione, the TCs liberated the liposomes efficiently, while the diameter of the released liposomes showed a slight increase compared to their diameter before coupling to the cell surface. It was also noticeable that coupling to the liposomes would not impair the antitumor efficiency of the genetically engineered cytotoxic TCs against OVA expressing lymphoma cells in vitro. Further, the ability of the developed liposome‐coupled TCs for carrying siRNA was tested. Because of the siRNA molecules’ negative charge, they were primarily loaded into a cationic dextran‐based nanogel (NG), then enveloped into the liposomes, and finally attached to the TC's surface. This platform promised tumor‐targeted delivery, bioresponsive release, and nucleic acid protection until being released in the proper site.

**Figure 10:**
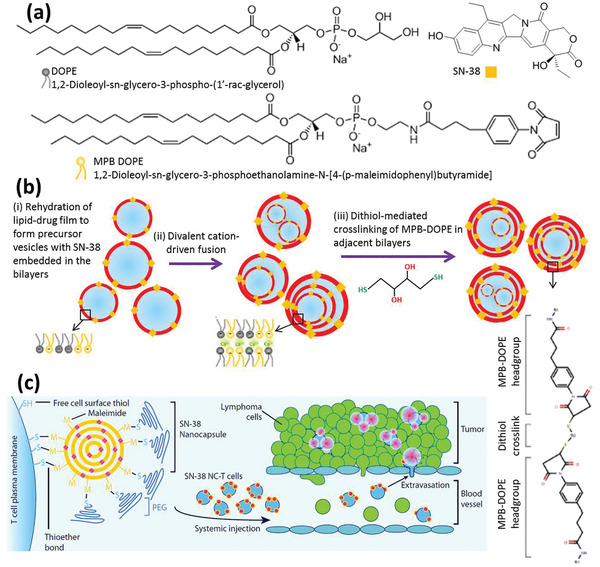
SN‐38‐loaded multilamellar liposomes attached to the TC's surface for targeting disseminated lymphoma tumors. a) Components of the nanoliposomes. b) The procedure for fabricating the NPs. c) Schematic of cell‐mediated delivery of SN‐38 NCs into tumors via TC functionalization. Reproduced with permission.^[^ ^102^ ^]^ Copyright 2015, American Association for the Advancement of Science.

Multilamellar lipid vesicles have been widely used for backpacking the TCs. Huang et al. have reported exploiting TCs to carry the SN‐38‐loaded multilamellar liposomes to the disseminated lymphoma tumors.^[^ ^102^ ^]^ They covalently binded the thiol‐reactive maleimide groups on the NPs with the free thiol groups on the TCs’ surface.^[^ ^98b^ ^]^ In brief, precursor liposomes were fabricated in a mixture of phosphatidylglycerol lipid, maleimide‐headgroup lipid, and SN‐38, fused together, and covalently crosslinked together to form multilamellar NPs (**Figure** **10**a,b) with an average diameter of 340 ± 12 nm, containing 14.3 µg SN‐38 per 1 mg of lipid. Then, NPs were incubated with TCs for surface conjugation, and not‐reacted maleimide groups were further quenched by PEGylation (Figure 10c). In a coculture with E*μ*‐myc lymphoma cells, it was observed that engineered TCs could significantly reduce the viability of the tumor cells even in a 1:20 ratio of TCs to tumor cells. In vivo studies suggested that the engineered TCs, while maintaining their cargo, could home in the lymphoid organs with a kinetic similar to that of nonengineered ones, confirming that TCs viability and physiological functions would not change across the engineering process. Furthermore, NP‐TCs led to 63 times higher tumor accumulation of SN‐38, about 12 times stronger reduction in the tumor burden, and significant animal survival compared to the NPs alone.^[^ ^102^ ^]^

**Figure 11:**
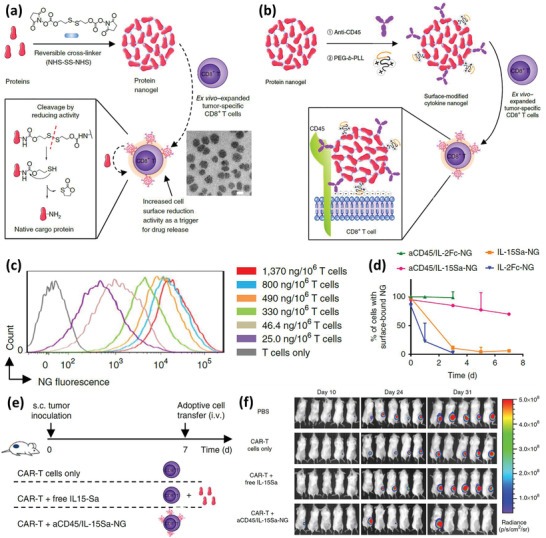
a) Scheme of NG synthesis and protein release in response to TC reducing activity in the local microenvironment. TEM image of NGs prepared from IL‐15Sa is shown. Scale bar is 50 nm. b) Schematic representation of surface modification of cytokine‐NGs with anti‐CD45 Ab and PEG‐PLL to facilitate stable attachment to T cell surfaces. c) Flow cytometric analysis of primed pmel‐1 CD8+ TCs that were coupled with fluorescently labeled aCD45/IL‐15Sa NGs at the indicated cytokine levels. The NG fluorescence showed that the maximum amount of IL‐15Sa loading on 10^6^ TCs is 800 ng. d) Flow cytometric analysis of primed pmel‐1 CD8+ T cells that were conjugated with aCD45/cytokine‐containing or cytokine‐only‐containing NGs. The results demonstrated the role of aCD45 on the prolonged surface remaining of the NGs. e) Experimental scheme of animal studies. Luciferase‐expressing U‐87 MG human glioblastoma cells (1.0 × 10^6^) were injected into NSG mice (*n* = 5 mice per group) via s.c. route. Mice received adoptive transfer of human T cells (2.6 × 10^6^ total cells, 38% transduced with EGFR‐targeting CAR (1.0 × 10^6^ CAR‐TCs)) intravenously on day 7. Treatments included sham saline, CAR‐TCs alone, CAR‐TCs + 13.8 µg of free IL‐15Sa, or CAR‐TC coupled with aCD45/IL‐15Sa‐NGs (13.8 µg). f) In vivo bioluminescence imaging of luciferase‐expressing U‐87 MG tumor cells over time. Reproduced with permission.^[^ ^98c^ ^]^ Copyright 2018, Springer Nature.

The development of TCs with CARs has been a promising strategy in cell therapy, offering a flexible platform to target any antigen of choice.^[^ ^103^ ^]^ Tang et al. have developed a strategy to “backpack” large quantities of IL‐15 superagonist complex (IL15‐sa) in the form of carrier‐free protein NGs on CAR‐TCs specified to target epidermal growth factor receptor (EGFR) in a human glioblastoma mouse model.^[^ ^98c^ ^]^ The NGs would be able to selectively release the protein in response to TC receptor activation. As can be observed in **Figure** **11**a, TC surface‐conjugated NGs can respond to an increase in T cell surface reduction potential after antigen recognition, resulting in drug release at the site of antigen encounter in the tumor microenvironment. A disulfide‐containing bis‐*N*‐hydroxy succinimide crosslinker (NHS‐SS‐NHS) was used for the solution‐phase reaction with cargo proteins to prepare NGs composed of many copies of the protein molecules that were crosslinked to themselves. Different cytokines and Abs were tested for the formation of NGs and all showed a high incorporation efficiency (>90%) with a high mass fraction of protein cargo (≈92% of dry weight). The NGs had a mean hydrodynamic diameters of ≈80–130 nm and they possessed slightly negative zeta potentials. The disulfide crosslinker in the backbone of the NGs was cleaved in response to reducing conditions at the TC surface, allowing the release of unadducted protein molecules via a self‐immolative reaction. The main studied protein cargos were ALT‐803, a human IL‐15Sa that is currently in clinical trials against hematological malignancies and solid tumors, and IL‐2Fc, which is a fusion protein between IL‐2 and an Ab fragment crystallizable (Fc) fragment. The release studies showed glutathione (GSH), a reducing agent, could accelerate the release of IL‐15Sa from NGs due to the cleavage of disulfide bonds. To have sustained effect, NG backpacks must remain on the surface of the cells instead of being internalized by the carrier cell. To this end, various surface receptors were tested and among all, anti‐CD45 Ab showed prolonged cell surface retention. Therefore, this Ab was used for the surface modification of the TCs (Figure 11b) without inhibiting TC proliferation in response to anti‐CD3/CD28 beads, concluding that CD45 binding would not restrict T cell receptor (TCR) signaling. A small quantity of poly(ethylene glycol)‐*b*‐poly(l‐lysine) (PEG‐PLL) was also adsorbed on the NGs immediately following the synthesis reaction via covalent coupling of a portion of PEG‐PLL to residual crosslinker NHS groups. This reaction provided a uniform surface positive zeta potential and promoted electrostatic interaction between the NGs and cell plasma membrane (Figure 11b), increasing the total amount of NG loading per cell. With this approach, up to 800 ng of IL‐15Sa could be loaded on 10^6^ TCs (Figure 11c) and the cytokine‐NGs containing anti‐CD45 (aCD45) could retain on the surfaces of unstimulated TCs for at least 7 days (Figure 11d). T cells that were backpacked with anti‐CD45‐ and IL‐15Sa‐containing NGs (aCD45/IL‐15Sa‐NGs) and stimulated with anti‐CD3/CD28 beads could be expanded substantially higher than those of T cells that were pulsed with the same total amount of free IL‐15Sa. In addition, the toxicity of the aCD45/IL‐15Sa‐NGs was significantly less than the free IL‐15Sa. Finally, it was demonstrated that the aCD45/IL‐15Sa‐NGs backpacked CAR‐TCs vigorously expanded and efficiently eradicated tumors as compared to the CAR‐TCs alone or TCs supplemented with a systemic dose of free IL‐15sa (Figure 11e,f).

Despite the successful results of CAR‐TC therapy in blood cancers this approach has had limited application for treatment of solid tumors, mainly due to the immunosuppressive tumor microenvironment, the aberrant perfusion that impedes TC access to the tumor cells, and also cellular heterogeneity of the tumor mass and formation of escape variants in the tumors that can evade from being recognized by CAR‐TCs. Nevertheless, various studies have been conducted to engineer the CAR‐TCs or the tumor microenvironment to overcome these hurdles.^[^ ^104^ ^]^

To ameliorate the suppressive microenvironment of the tumors, Siriwon et al. incorporated a small molecule agonist of A2a adenosine receptor, SCH58261, within the NPs that were attached to the CAR‐TCs’ surface to be released in the tumor microenvironment and inhibit the immunosuppressive effect of the adenosine released by the tumor cells on the TCs.^[^ ^105^ ^]^ They took advantage of crosslinked multilamellar liposomes conjugated to the thiol groups of CAR‐TCs’ surface through their maleimide containing lipids, while the nonreacted maleimide groups were further PEGylated. It was observed that up to 287 ± 49 NPs could be attached to a single cell surface, being distributed in several clusters on the surface. No significant changes regarding the CAR‐TCs ability to migrate, recognize the target cells, secrete IFN‐*γ*, and their antitumor efficacy were detected following NP attachment to their surface. Being attached to the CAR‐TCs, NPs had higher tumor recruitment and deeper tumor infiltration in vivo. It was also observed that the released SCH58261 could augment the activity of tumor‐residing TCs. This approach resulted in significantly delayed tumor growth and prolonged animal survival.

**Figure 12:**
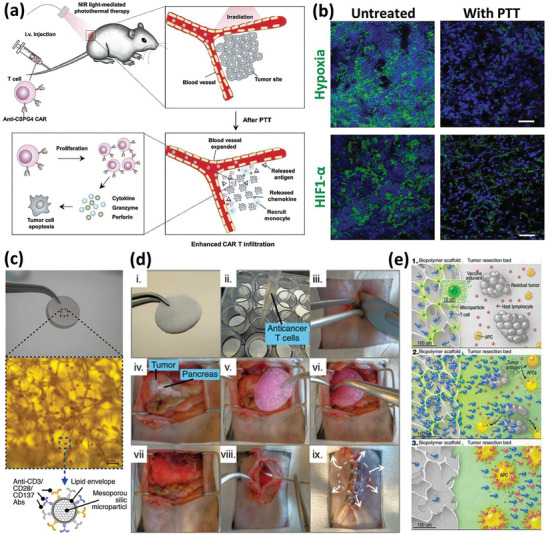
a) Schematic depiction demonstrating the effects of the mild NIR mediated heating of the tumor on the enhanced infiltration and activation of adoptive anti‐CSPG4 CAR‐TCs. b) Representative images of hypoxia and HIF1‐*α* immunofluorescence staining of the tumors before and after PTT. The blue areas are stained with DAPI for the recognition of tumor cells and green areas demonstrate hypoxia and HIF1‐*α* active regions (scale bar is 50 µm). Reproduced with permission.^[^ ^106^ ^]^ Copyright 2019, Wiley‐VCH. c,d) Polymeric biomatrices placed directly on pancreatic tumors can effectively deliver programmed CAR‐TCs. c) Photograph of the scaffold, bright‐field microscopy of stimulatory microspheres incorporated into it, and a schematic depiction of the composition of the microspheres. Scale bar is 70 µm. d) This series of images shows: i) biopolymeric scaffold; ii) seeding of tumor‐reactive TCs into the scaffold; iii) incision; iv) orthotopic KPC pancreatic tumor; v) implantation of the TC‐loaded scaffold; vi) wound closure; vii–ix) sustained release of tumor‐reactive T cells. e) Schematic illustration of mechanism, by which the adjuvant, microparticle, and CAR‐TC coloaded scaffold can eradicate the tumor cells. The released molecules can prime host immune cells to recognize and lyse tumor cells. Reproduced with permission.^[^ ^107^ ^]^ Copyright 2017, American Society for Clinical Investigation.

To address insufficient tumor recruitment of the TCs, Chen et al. modified the tumor environment with laser irradiation.^[^ ^106^ ^]^ They reported that by inducing mild hyperthermia in the tumor region with PTT, the chondroitin sulfate proteoglycan‐4 (CSPG4)‐specific CAR‐TCs were recruited in the tumor tissue and their proliferation and cytokine production was increased (**Figure** **12**a). The enhanced recruitment of cells was the result of partial disruption of the extracellular matrix (ECM) and reduced interstitial fluid pressure in the tumor tissue, which allowed enhancement of the blood perfusion. This assumption was proved by fluorescence imaging of the reduced signals of the hypoxia probe pimonidazole and the hypoxia‐inducible factor (HIF)‐1*α* as a result of enhanced oxygenation in the tumor tissue after NIR laser irradiation (Figure 12b). Moreover, combined PTT and CAR‐TC therapy could enhance the intratumoral population of MCs and DCs, as well as the concentration of cytokines, significantly suppressing the tumor growth and in some cases led to tumor eradication.

Smith et al. have also harnessed an implant containing CAR‐TCs and immune adjuvants to address problems associated with the solid tumors’ CAR‐TC therapy, i.e., the tumor access, the tumors’ immunosuppressive environment, and the tumors’ cell heterogeneity.^[^ ^107^ ^]^ First, a pancreatic cancer‐specific TC was developed through TC engineering with a retrovirus encoding a chimeric NK receptor. Then, a polymeric implant was applied to transfer the CAR‐TCs directly on the surface of the solid tumors through surgery (Figure 12c,d). This biocompatible and biodegradable implant was composed of a mesoporous alginate‐based scaffold modified with a synthetic collagen‐mimetic peptide (GFOGER) as migration‐promoting macromolecules. Moreover, a biomimetic silica‐based microparticulate system that was enveloped in a phospholipid bilayer and surface modified with stimulatory cues, i.e., anti‐CD3, anti‐CD28, and anti‐CD137 Abs, was encapsulated within the scaffold (Figure 12c). The microparticles were able to strongly induce TC proliferation as soon as they become released. The microparticles could also carry a cyclic dimeric guanosine monophosphate (di‐GMP), which is a stimulator of IFN genes (STING) pathway agonist, here serving as a vaccine adjuvant along with the tumor's components as the antigen, to improve the procedure of antigen‐presenting in the tumor microenvironment. After the CAR‐TCs occupied the empty spaces of the scaffold, the device was tested in an inoperable pancreatic cancer and a partially resected melanoma mouse model. It was revealed that highly activated TCs with amplified proliferation rate successfully left the scaffold and killed the tumor cells, developing an in situ vaccine production site by releasing the adjuvant in the presence of the whole‐cell antigen, inflammatory cytokines and the native immune cells (antigen‐presenting cells and T cells) attracted to the site (Figure 12e). Summing up, the developed device induced significant antitumor effect by directly delivering CAR‐TCs to the tumor site, overcoming the immunosuppressive signals of the solid tumor, as well as minimizing escape variants’ survival through vaccination with whole‐cell antigen.^[^ ^107^, ^108^ ^]^

In general, although modifying CAR‐TCs with nanomaterials has been a successful strategy in a research setting, there is still a long distance to the clinic. Nevertheless, CAR‐TCs alone have already received attention for their antitumor effects in patients who do not respond to common treatments. Kymriah and Yescarta are two autologous CAR‐TC‐based therapies being approved by FDA for B cell precursor acute lymphoblastic leukemia and large B cell lymphoma, respectively. However, because of their potential risks and shortcomings that have not been fully explored, their broad application is still doubted.^[^ ^99^, ^109^ ^]^

### 3.3 Other Surface Engineered Immune Cells

In addition to M*ϕ*s and TCs, different biomaterial‐based nongenetic engineering approaches for surface modification of other immune cells have been harnessed to improve their therapeutic capabilities. For example, natural killer cells (NKCs) have been widely studied for cancer cell therapy.^[^ ^110^ ^]^ NKCs do not recruit in the tumor milieu based on antigen recognition; rather their homing in the tumor site is usually based on the chemokines’ gradient.^[^ ^111^ ^]^ Hence, the immunosuppressive signals in the solid tumors’ microenvironment significantly hamper the NKCs migration and infiltration to the tumor. To address this issue, Burga et al. leveraged a magnetic force for guiding the NKCs to the tumor site.^[^ ^112^ ^]^ First, NKCs were obtained from umbilical cord blood and their membranes were biotinylated to react with streptavidin‐coated iron oxide NPs (IONPs. It was observed that the conjugation with IONPs and being exposed to a magnetic field for 10 min would affect neither the NKCs’ viability nor their phenotype. It was also demonstrated that in the presence of the magnetic field these IONP‐conjugated NKCs revealed rapid and significant infiltration into a 3D model of neuroblastoma, leading to increased cytotoxic response compared to pristine NKCs.

**Figure 13:**
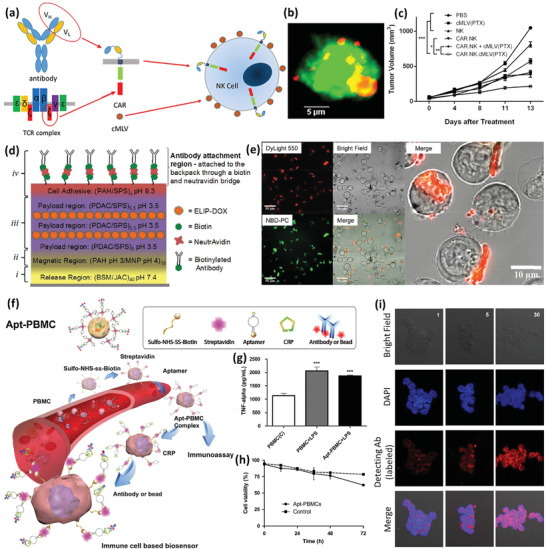
a) Schematic depiction of CAR‐NKCs conjugated to PTX‐loaded cMLVs. CARs were derived from the single‐chain variable fragment (scFv) of an Ab and the TC receptor signaling complex. CARs were transduced into NK92 cells, and cMLVs were conjugated to the cell surface by interaction with free thiol groups. b) Fluorescence image of CAR‐NKCs conjugated to DiD‐loaded cMLVs. CAR‐NKCs were labeled with CFSE before conjugation to DiD‐labeled cMLVs. Scale bar is 5 µm. c) Tumor growth inhibition in different groups treated with different formulations. Reproduced with permission.^[^ ^113^ ^]^ Copyright 2017, American Society of Gene and Cell Therapy. d) Scheme of the final backpack composition and layers with the release region composed of PDAC/SPS for attachment to MCs. e) Representative fluorescence images of ELIP backpacks anchored to MCs. The green fluorescence originated from phospholipids that were fluorescently labeled and embedded into the liposomes, while the red fluorescence was a result of fluorescently labeled NeutrAvidin in the attachment region (DyLight 550). Reproduced with permission.^[^ ^114^ ^]^ Copyright 2015, Wiley‐VCH. f) Schematic of CRP detection by aptamer‐conjugated PBMCs. Sulfo‐NHS‐SS‐biotin was crosslinked to PBMCs, followed by the introduction of streptavidin to attach to the biotin. Next, aptamer was conjugated to the PBMCs (Apt–PBMCs). In the blood circulation, the complex can recognize CRP and form a CRP–aptamer–PBMC complex, and then, a detectable fluorescence signal was emitted by attaching Ab coated‐beads or anti‐CRP Abs to the conjugated complex. g) TNF‐*α* profiling assay after treating both PBMCs and Apt–PBMCs (1 × 10^5^ cells per well) with LPS (0.2 µg mL^−1^) for 24 h (^***^ *p*‐value < 0.0001). h) Trypan blue assay for the counting of viable PBMCs and Apt–PBMCs (seeding density = 1.5 × 10^5^ per well) after 72 h. i) Fluorescence images of CRP capturing by Apt–PBMCs using a labeled fluorescence Ab at three different concentrations of CRP (1, 5, and 30 mg L^−1^). Reproduced with permission.^[^ ^116^ ^]^ Copyright 2016, Springer Nature.

Another potent hybrid system based on NKCs has been introduced by harnessing the CAR technology as well as a surface modification with therapeutic‐loaded multilamellar liposomes (**Figure** **13**a).^[^ ^113^ ^]^ First, the NKCs were irradiated to lose their ability to proliferate, but not their ability to kill the tumor cells. Second, they were engineered to express anti‐CD19 or anti‐HER2 Ab through distinct retroviral and lentiviral transduction, respectively. Then, PTX‐carrying crosslinked multilamellar vesicles (cMLVs) based on maleimide‐containing lipids were attached to the free thiol groups on the NKCs’ surface and not‐reacted maleimide groups were further quenched by PEGylation. It was determined that the optimum vesicles to cells ratio was 1000 to 1 and about 150 cMLVs could be attached to each cell. It also revealed that the cMLVs’ attachment did not lead to their significant internalization (Figure 13b) or altered migration ability and IFN‐*γ* production of the cells. Further analyses on tumor cells suggested that sustained drug release provided by the cMLVs would intensify the antitumor effect of the NKCs, and combined with the CAR‐mediated response in the presence of HER2 or CD19 positive tumor cells, strong tumor toxicity could be expected. In vivo biodistribution studies involving a xenograft model of CD19^+^ ovarian cancer in mice demonstrated that after about 72 h, most of the free cMLVs were cleared by the liver, while those attached to NKCs had a significant presence in the circulation, lymph node, spleen, and the tumor site. It was also observed that mice receiving separate CAR‐NKCs or PTX‐loaded cMLVs therapies had less antitumor efficacy compared to CAR‐NKCs carrying PTX‐loaded cMLVs (Figure 13c). Moreover, because of the dose reduction as a result of targeted delivery of PTX, no cardiotoxicity was detected in the animal subjects.

For designing a more complex cell backpack platform for targeted and controlled release drug delivery, Polak et al. proposed incorporating drug‐loaded echogenic liposomes (ELIPs) into micrometer‐size polymeric patches attached to the MCs’ surface.^[^ ^114^ ^]^ Liposomes were fabricated based on a protocol introduced by Huang and MacDonald^[^ ^115^ ^]^ and were loaded with DOX as a model drug. The drug‐loaded liposomes had a negative zeta‐potential and a broad size distribution from 100 to 1000 nm. Scanning electron cryomicroscopy (cryo‐SEM) images proved the presence of air pockets, making the liposomes ultrasound‐responsive. The ELIPs were embedded into polymeric backpacks through electrostatic interactions and layer‐by‐layer (LbL) assembly between poly(allylamine hydrochloride)/poly(acrylic acid) (PAH/PAA)*_n_* and poly(diallyldimethylammonium chloride)/poly(styrene sulfonate) (PDAC/SPS)*_n_*. The cell backpacks were first attached to the surface of a glass slide with layers as follows (Figure 13d): release region (bovine submaxillary mucin and jacalin lectin; being decomposed to detach the backpacks from the slide), the magnetic region (PAH+magnetic NPs; for purification of the backpacks), the payload region (as described above), and the cell adhesive region (IgG; for interaction with the MCs’ surface). It was observed that the primary loading of DOX in the liposomes improve the total backpacks’ drug loading by ninefold for the PAH/PAA compared to the direct loading of DOX within the polymeric layer. As shown, the ELIPs were adsorbed on the polymeric films to form the double sandwich‐like structures of the patches’ payload region via LBL assembly. After backpack formation on the glass slide, they were separated from the glass slides and incubated with mouse MCs for attachment to their surface without any significant phagocytosis and internalization (Figure 13e).^[^ ^114^ ^]^

An important application for surface‐engineered alive immune cells, rather than the delivery of therapeutics and diagnostics, could be the biosensing of disease markers with high specificity and accuracy. To this end, Hwang et al. have introduced a platform for detecting C‐reactive protein (CRP), as an inflammatory disorder marker, based on the aptamer‐conjugated peripheral blood mononuclear cells (Apt–PBMCs) (Figure 13f).^[^ ^116^ ^]^ Aptamers were attached to the surface of PBMCs via streptavidin–biotin reaction. When stimulated by LPS, the aptamer‐conjugated PBMCs released about ≈90% TNF‐*α* as the pristine PBMCs did (Figure 13g), and it was confirmed that the conjugation has not impaired the cell's function. It was revealed that about 65% of the cells maintained their viability 3 days postconjugation (Figure 13h). Then, the developed platforms were tested to detect CRP in the concentrations expected in the physiological range, i.e., 0–30 mg L^−1^, resulting in a limit of detection of 0.05 mg L^−1^. In addition, as proved by confocal imaging (Figure 13i), increasing the concentrations of CRP elevate the fluorescence intensity due to enhanced complexation between the CRP and the Apt–PBMC. Interestingly, it was also observed that in a microfluidic channel, the aptamer–PBMCs migrated to the sites with higher CRP concentration, which proposed the potential application of these cells for CRP detection in vivo.

## 4 Whole Immune Cell Membrane or Its Proteins for Biomimetic Drug Delivery and NP Coating

Nowadays, there is no doubt in the potential of nanotechnology to introduce more effective and safer therapeutics into the clinic. Physicochemical characteristics of nanotherapeutics, including their size, zeta potential, and significantly, their surface chemistry, directly dictate their biofate.^[^ ^117^ ^]^ In the past decades, a variety of techniques, such as surface conjugation with different ligands like aptamers, Abs, or peptide have been used for tracking, recognizing, and targeting of the NPs.^[^ ^118^ ^]^ In the past decade, a new approach of surface modification of NPs is exploited using the immune cell membrane, not only providing a versatile platform for further modifications but also to make the NPs stealth to evade being cleared by the reticuloendothelial system (RES).^[^ ^119^ ^]^ Notably, important merit of coating NPs with the cell membrane rather than incorporating the NPs within the live cells is that the particles maintain their nanoscale size that allows prominent tissue penetration, while alive cells usually have a micrometer‐size scale. Moreover, there will be no concern about the toxicity of the payload toward the viability and functionality of its cell vehicle, which improves the yield and reduces the costs of fabricating these biomimetic therapeutics compared to their alive cell‐based counterparts.^[^ ^120^ ^]^ The cell membrane itself can also load free drugs or ultrasmall NPs within its phospholipid bilayer, while its surface can also undergo modification from peptide or Abs. In addition, hybrid wraps can be fabricated by mixing immune cell components with that of other cell types like other types of immune cells, red blood cells (RBCs), platelets, or even tumor cells.^[^ ^119^, ^121^ ^]^ For instance, a group of researchers has reported exploiting the whole human blood leukocytes’ membrane amalgamated with platelets’ membrane to fabricate immunomagnetic beads for highly efficient recognition and isolation of circulating cancer cells.^[^ ^121a^ ^]^ To this end, white blood cells (WBCs) and platelets were extracted from human blood samples and their membranes were isolated to form a hybrid vesicle. Next, the vesicles were coated on magnetic beads of about 100 nm and further functionalized with anti‐epithelial cell adhesion molecules (EpCAMs) Ab with biotin‐streptavidin interaction to obtain the final NPs. These NPs had a shell with a thickness of about 9 nm and significant colloidal stability and superparamagnetic properties. In coculture with EpCAM positive tumor cells, the capturing efficiency of Ab conjugated hybrid shell–magnetic core NPs was about 95%. As expected, based on the reported crosstalk between platelets and circulating tumor cells (CTCs), the NPs covered with hybrid or platelet shell could capture more tumor cells both in the coculture studies and in the human blood medium, compared to the naked or WBC wrapped magnetic beads. Besides, using platelet membranes in the NPs’ shell did not seem to enhance NPs’ interaction with nontumor cells. Finally, the capturing efficiency of the developed hybrid NPs was evaluated in the blood samples received from 20 breast cancer patients. The robust results demonstrated that hybrid platelet–leukocyte membrane‐derived shells had great potential for being introduced as a personalized diagnosis platform to the clinic, due to their high cell capturing efficiency.

Several methods have been reported for isolating immune cells’ membrane, which allow the functionality of its constituents to be preserved. To empty the intracellular components, hypotonic lysis, repeated freeze–thaw cycles, and mechanical cell rupture for example by nitrogen cavitation have been proposed.^[^ ^122^ ^]^ After cell membrane extraction, they can be utilized to cover NPs without undergoing fractionation. For this aim, coextrusion of the isolated cell membranes through a mini extruder with desired pore size can be applied either to coat hybrid membrane or to cloak a single type of cell membrane on the NPs. Another method for this purpose is to cosonicate the cell membrane and NPs. Novel microfluidic approaches have been introduced based on the electroporation of the cell membrane and then incorporating the NPs within them.^[^ ^122^ ^]^

Utilizing distinct types of immune cell membranes, rather than a combination of immune cells found in the total buffy coat, to wrap NPs and endow them with unique properties based on the cell type.^[^ ^122b^ ^]^ Generally, being cloaked in the leukocytes’ membrane increases NP residence time in the circulation and leads to their facilitated transport to the inflammation site via being attached to the inflamed endothelial cells, while circumventing the lysosomal pathway of these cells and hence being protected from degradation.^[^ ^119b^ ^]^ M*ϕ*CM improves adhesion of NPs to the tumor cells,^[^ ^123^ ^]^ and NEs membrane facilitates their attachment to the circulating cancer cells.^[^ ^124^ ^]^ Harnessing the TCs membrane can be useful for recognizing and targeting specific antigens and cells.^[^ ^125^ ^]^

Another important issue regarding the source of the cell membrane is that syngeneic leukolike vectors (LLVs) are preferred to the xenogeneic LLVs because the NPs covered with the syngeneic LLVs would be less internalized and are more biocompatible. This was concluded from an in vivo study on LLVs from murine M*ϕ*s’ membrane and that of human TCs in mice.^[^ ^126^ ^]^ Based on the direct effects of the cell membrane source on the physicochemical properties and pharmacokinetics of the payloads whom they cover, many publications have reported utilizing membranes from a single cell type. Furthermore, despite the superiority of using syngeneic cell membranes, purity and convenience of working with commercial cell lines have led to the prevalent application of xenogeneic ones. Herein, a comprehensive review of the most recent attempts for encapsulating therapeutics and drug‐carrying NPs within various immune cells’ membrane is provided.

### 4.1 Dendritic Cell Membrane for Drug Delivery and NP Cloaking

Because of their essential role in the immune system, DCs have been the subject of many efforts for developing biomimetic DDSs.^[^ ^127^ ^]^ As DCs naturally produce a significant quantity of ECVs, there are relatively few reports on utilizing the DCs’ membrane prepared via cell extrusion for drug delivery purposes. Nevertheless, because of the speed and high yield of cell extrusion technique, Wan et al. developed aptamer‐conjugated DC membrane‐based vesicles for targeted delivery of PTX to the tumor site.^[^ ^128^ ^]^ The nucleolin‐binding aptamer, AS1411, was first attached to the cholesterol‐PEG2000 to be efficiently incorporated within the lipid bilayer membrane of the vesicles. Then the aptamers and DCs were coextruded to produce targeted vesicles. Comparing the extrusion‐based strategy with the natural process of producing ECVs, the extrusion‐based one had a 17‐fold higher yield and was 115 times faster than the natural secretion from the cells. The produced NPs, after cryoprotection and freezing, revealed acceptable stability regarding the particle size over 6 months. The aptamer‐conjugated nanovesicles were then incubated or cosonicated with PTX and it was observed that sonication would lead to a higher loading efficiency of 21.33 ± 1.53% as compared to 5.06 ± 1.12% for incubation approach. The spherical vesicles had a 111 nm diameter and a zeta‐potential of −25.6 mV. Drug release from the aptamer‐conjugated nanovesicles had an initial phase of burst release during the first hour, followed by a sustained release phase until 24 h, releasing about 71.47% of its PTX content. In vitro studies revealed that PTX loaded aptamer‐conjugated nanovesicles had a ≈15‐fold decreased IC50 compared to the free PTX for the triple‐negative breast cancer cells. Besides, in the same dose of PTX, the PTX‐loaded nanovesicles led to G2 arrest in ≈90% of the tumor cells, while the free drug molecules only inhibited about 60% of the tumor cells. This observation was mainly due to the nanovesicles potential for intracellular drug delivery. In vivo pharmacokinetic studies demonstrated significantly higher tumor accumulation and tumor retention for drug‐loaded nanovesicles compared to the free drug, leading to a ninefold higher antitumor efficiency, as well as decreased systemic toxicity.

### 4.2 Biomedical Application of Extracted Proteins or Whole Plasma Membranes of Macrophage and Monocyte

M*ϕ*s have several important features that make them suitable for coating NPs and developing biomimetic DDSs.^[^ ^129^ ^]^ First of all, they are present all around the body and are one of the first cells to reach sites of inflammation. Second, they are important parts of the RES and phagocytose a wide range of invading particles (bacteria, viruses, etc.), while they do not react with the body's own cells. Third, they have broad crosstalk with the tumor tissues. Putting all these together, coating the payloads, such as NPs with M*ϕ*CM can potentiate them for prolonged circulation, trafficking to inflammation sites, and specifically being attached to cancer cells.^[^ ^120^, ^122^, ^130^ ^]^

In addition to the whole‐cell membrane, a series of studies have reported incorporating M*ϕ*CM proteins in the synthetic lipid bilayers for therapeutic applications. For example, Molinaro et al. used membrane proteins from murine M*ϕ* J774 cell line to be incorporated within a lipid bilayer composed of cholesterol and choline‐based phospholipids.^[^ ^131^ ^]^ The optimized unilamellar carriers have a protein to lipid weight ratio of 1:300 with a mean diameter of about 120 nm and zeta‐potential of −13.8 mV. The protein composition analysis of the carrier distinguished 342 distinct proteins with various functions, including being involved in transport (48%), signaling (16%), immunity (12%), cell adhesion (9%), lipid metabolism (5%), and structure (4%). It was confirmed that this concentration of critical leukocyte surface proteins, specifically those being involved in cell adhesion (e.g., lymphocyte function associated antigen‐1 (LFA‐1) and macrophage adhesion molecule‐1 (Mac‐1)) and self‐tolerance (e.g., CD45), on the carriers’ surface was enough for their expected biological activity. Three model drugs, including dexamethasone (hydrophilic), caffeine (amphiphilic), and PTX (hydrophobic) were demonstrated to be successfully loaded into the carriers without affecting their size and shape. Nevertheless, the zeta‐potential of the particles were slightly changed. Compared to the liposomes with no leukocyte proteins, the developed combined carriers had increased circulation and accumulation in the inflamed tissue, which was mediated by their active adhesion (with the direct involvement of LFA‐1 and indirect role of CD45) to the damaged endothelium. Martinez et al. reported harnessing the above‐mentioned carriers for imaging the activated vasculature in breast tumors and atherosclerotic plaques, due to their inflammation targeting properties.^[^ ^132^ ^]^ It was demonstrated that after 1 and 6 h, cell membrane protein combined liposomes had 15 and 9 times higher tumor accumulation, respectively, as compared to the simple liposomes. In addition, the efficiency of the carriers for targeting atherosclerotic plaques in a hypercholesterolemic mouse model showed that after 2 h of systemic administration, the cell membrane protein combined liposomes had a fourfold increased targeting and accumulation in the lesions of the mice's aorta compared to the liposomes. In conclusion, leukosomes appear to be a versatile platform for incorporating therapeutics and diagnostics, with the potential to be introduced to the clinic.

**Figure 14:**
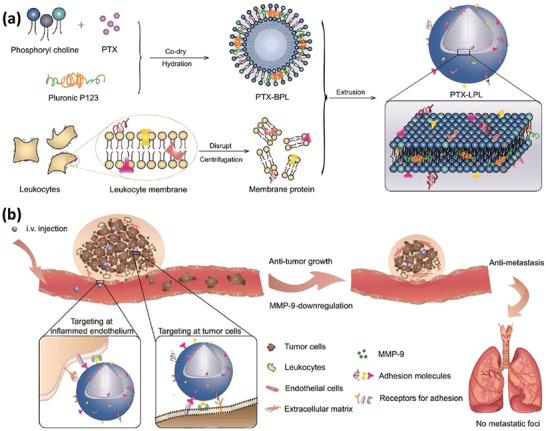
a) Pluronic P123‐lipids‐leukocyte membrane proteins liposome for breast cancer targeting and preventing lung metastasis. Leukocyte membrane proteins were used to confer biomimetic functions to the particles. b) Antitumor growth was obtained by PTX, while Pluronic P123 did not allow the lung metastasis of breast cancer. Leukocyte membrane proteins were used for targeting and biomimicry purposes. Adapted with permission.^[^ ^133^ ^]^ Copyright 2019, Royal Society of Chemistry.

In another attempt, Chen et al. have reported developing a leukocyte‐mimetic platform for PTX delivery to breast cancer to inhibit its growth and metastasis.^[^ ^133^ ^]^ First, they fabricated lipid vesicles based on a mixture of phosphorylcholine lipids and pluronic P123 that contained PTX. Then, the membrane proteins from murine M*ϕ* cell line J774A.1 were incorporated in the vesicles via extrusion following a thin layer evaporation (TLE) procedure (**Figure** **14**a). To form PTX‐loaded pluronic‐lipid liposome (PTX‐BPL), 1,2‐dipalmitoyl‐*sn*‐glycero‐3‐phosphocholine (DPPC), 1,2‐distearoyl‐*sn*‐glycero‐3‐phosphocholine (DSPC), 1,2‐dioleoyl‐*sn*‐glycero‐3‐phosphocholine (DOPC), and cholesterol (5:1:3:1 molar ratio, respectively) were dissolved in a chloroform:methanol mixture (3:1 v/v). Then, pluronic P123 was added to lipids at a molar ratio of 1:9, followed by codrying via solvent evaporation using vacuum‐rotary evaporation to form a thin film. The thin film was further hydrated with PBS to assemble pluronic‐lipid liposomes (BPL). The biomimetic vesicles were prepared by mixing PTX‐BPL suspensions with purified leukocyte membrane (30:1 w/w lipid‐to‐protein ratio) before extrusion through a cellulose acetate membrane ten times. The final size of the particles was 175.4 ± 1.1 nm and zeta‐potential was −20.7 ± 0.8 mV. The protein layer also slightly delayed PTX release from the liposomes compared to nonprotein containing particles. In vitro assays demonstrated that when biomimetic NPs were incubated with cocultured breast cancer and M*ϕ* cells, they were more effectively internalized by the breast cancer cells and less phagocytosed by M*ϕ*s. This happened because of the biologically active LFA‐1 membrane proteins on the particles that allowed their increased attachment to the tumor cells, while mimicking the surface property of the real M*ϕ*s. The in vivo results showed that the leukocyte‐mimicking pluronic‐lipid nanovesicle hybrid could target breast tumor site and also suppress metastasis. The lipid vesicle was endowed with biological functions of leukocyte protein to engage in targeting at the tumor, while pluronic P123 could preserve its antimetastasis effect by inhibiting the tumor cells from escaping to form the metastatic foci at distant organs (Figure 14b). Leukocytes express LFA‐1 and Mac‐1 on their surface, which are receptors for intercellular adhesion molecule 1 (ICAM‐1) that is highly expressed in inflamed endothelial cells and metastatic tumor cells. Therefore, leukocyte membrane proteins can target inflamed endothelium in the vicinity of the tumor tissue and also accumulate in their target cancer site by attachment to ICAM‐1 in the tumor microenvironment.

The antimetastasis effect of pluronic P123 comes from its inhibitory effect on matrix metalloproteinase‐9 (MMP‐9) that induce the degradation of the ECM and help tumor cellsʼ escape from the primary cancer site (Figure 14b).

In addition to the leukocytes' surface proteins, leukocytes’ whole membrane has also been investigated for camouflaging of various types of drugs or NPs to treat a variety of disorders. **Table** **1** demonstrates several outstanding studies in recent years that take advantage of whole M*ϕ*CM for improving biomimicry in the drug delivery platforms.

**Table 1:** Various nano‐ and microparticulate formulations being surface‐coated with the M*ϕ*CM proteins or the whole membrane

Particle^a)^	Fabrication method	Size [nm or µm]Zeta potential [mV]	Main findings	Refs.
UCNPs@M*ϕ*CM	1) M*ϕ*CMs were prepared from RAW 264.7 cells after hypotonic lysing, centrifugation, and passing through a 400 nm polycarbonate porous membrane. 2) M*ϕ*CMs fused onto the fluorescent UCNPs through repeated extrusion using a mini extruder through a 200 nm membrane porous membrane, followed by centrifugation to prepare UCNPs@M*ϕ*CMs.	≈100 nm ≈−20 mV	UCNPs@M*ϕ*CMs exhibited effective cancer‐targeting capability inherited from the mother M*ϕ* cells. Remarkable NIR fluorescence emission performance of the NPs exploited for highly effective cancer imaging. The blood biochemistry, histology analysis, and hematology studies confirmed the desirable in vivo biocompatibility of the UCNPs@*ϕ*CMs.	^[^ ^134^ ^]^
HPMCs@M*ϕ*CMs	1) Layer‐by‐layer assembly was performed to obtain chitosan–alginate‐coated SiO_2_ particles. The resulting particles were treated in HF solution as an etchant to remove the silica template and harvest HPMCs. 2) M*ϕ*CMs were coated on the HPMCs through extruding, followed by centrifugation.	≈2.5 µm −31.4 mV	HPMCs@M*ϕ*CMs could effectively decrease the opsonization and nonspecific clearance by the immune system, and thus, prolonged circulation of the biomimetic nanosystem was achieved in the mice. The M*ϕ*CMs endowed the HPMC capsules with a remarkable enhancement in their accumulation in tumor site in vivo due to the molecular recognition of the membrane‐bound proteins of M*ϕ*CMs with those of tumor cells.	^[^ ^135^ ^]^
DOX‐loaded HMSNs@M*ϕ*CM	1) M*ϕ*CMs were prepared by treating isolated macrophage cells with hypotonic lysing buffer, repeated grinding, centrifugation, and finally mechanical extrusion through a filter membrane. 2) M*ϕ*CMs were coated on the surface of the DOX‐loaded HMSNs.	65.1 nm −16.9 mV	Compared to HMSNs, the M*ϕ*CM‐coated HMSNs displayed high in vivo stability, increased circulation life, and reduced retention in RES organs. DOX encapsulation within HMSNs was 35.7%. In vivo studies demonstrated that the tumor growth was effectively inhibited by utilizing low doses of the therapeutic agent due to the high DOX‐loading capacity of HMSNs and their effective accumulation within the tumor site.	^[^ ^136^ ^]^
AuNS@M*ϕ*CM	1) M*ϕ*CMs were harvested from natural macrophage cells through repeated extrusion. 2) AuNS was formed through a seed‐mediated growth method on the surface of MSNs. 3) AuNSs were coated by M*ϕ*CMs using a mini extruder device.	≈100 nm −20.8 mV	AuNS@M*ϕ*CM exhibited good colloidal stability and biocompatibility, reduced opsonization, prolonged blood circulation time, and enhanced tumoritropic accumulation. Original NIR adsorption of AuNS was retained after camouflaging of the Au nanosystem by M*ϕ*CM. Tumor growth on 4T1 tumor‐bearing mice was effectively suppressed upon NIR irradiation (808 nm, 1 W cm^−2^) and almost disappeared after 25 days.	^[^ ^137^ ^]^
Fe_3_O_4_ NPs@M*ϕ*CM	1) Fe_3_O_4_ NPs were fabricated by the solvothermal method. 2) Fe_3_O_4_ NPs were coated by M*ϕ*CMs through repeated extrusion.	≈100 nm ≈−18 mV	Fe_3_O_4_ NPs@M*ϕ*CM exhibited good biocompatibility, high light‐to‐heat conversion efficiency, immune evasion, and cancer targeting abilities. Highly effective PTT of breast cancer in nude mice was achieved in vivo.	^[^ ^138^ ^]^
Fe_3_O_4_ NPs@M*ϕ*CM	1) After suspending of macrophage cells in a hypotonic lysing buffer, cell debris and nuclei were removed by centrifugation, and then differential centrifugation was used to isolate M*ϕ*CM. 2) Polyethyleneimine‐modified Fe_3_O_4_ NPs were synthesized using the hydrothermal method. 3) M*ϕ*CM‐coated Fe_3_O_4_ NPs were prepared by repeatedly coextruding the mixture of Fe_3_O_4_ NPs and M*ϕ*CM through a 100 nm porous polycarbonate membrane.	≈120 nm +7.6 mV at pH 5.5 and −19.5 mV at pH 7.4	M*ϕ*CM‐coated Fe_3_O_4_ NPs markedly neutralized bacterial endotoxin (also known as lipopolysaccharide), prevented the activation of macrophage cells, reduced the inflammatory reactions, and decreased the release of cytokines. The in vivo studies displayed that the NPs exhibited prominent biocompatibility as well as a remarkable protective effect toward endotoxemic mice.	^[^ ^139^ ^]^
PTX@leutusome	1) HN12 tumor and murine J774A.1 cells were selected as the source of tumor cells and leukocytes, respectively, to prepare composite cell membranes. 2) Exogenous phospholipids were employed as building blocks to fuse the cell membranes to form liposomal NPs named leutusomes. 3) The anticancer drug PTX was loaded into leutusomes to obtain PTX@leutusomes.	≈160 nm −21.6 mV	Original surface biomarkers of both cell membranes of leutusomes were confirmed by Western blot analysis. In vitro studies showed that leutusomes were taken up selectively by tumor cells while their uptake by leukocyte alone was significantly reduced. Leutusomes displayed high drug loading capacity, and the PTX@leutusomes could reduce the IC50 of the drug. The in vivo studies confirmed that the presence of both cell membranes endowed the NPs with prolonged blood circulation and efficient accumulation at the tumor site (79.1 ± 6.6% ID g^−1^ of tumor) was obtained. PTX@leutusomes remarkably inhibited tumor growth without causing systemic adverse effects.	^[^ ^140^ ^]^
AbNPs@M*ϕ*CM	1) Purified M*ϕ*CMs were harvested by using a combination of hypotonic lysis, mechanical membrane fragmentation, differential centrifugation, and then extrusion through a porous polycarbonate membrane. 2) AbNPs were prepared through the nanoprecipitation method and were loaded with PTX. 3) The coextrusion method using a 200 nm porous polycarbonate membrane was employed to encapsulate the drug‐loaded AbNPs within the M*ϕ*CMs.	188.7 nm −10.5 mV	Enhanced cellular uptake efficiency into B16F10 cells was observed by the AbNPs@M*ϕ*CM. The AbNPs@M*ϕ*CM showed prolonged blood circulation time and selective accumulation at the tumor site. Encapsulation efficiencies of PTX within AbNPs and AbNPs@M*ϕ*CM were 93.5 ± 2.1% and 83.4 ± 2.2%, respectively. PTX‐loaded AbNPs@M*ϕ*CM induced remarkable antitumor efficacy and targeted chemotherapy of melanoma were achieved in vivo.	^[^ ^129a^ ^]^
PLGA NP@M*ϕ*CM	1) M*ϕ*CMs were harvested from J774 mouse M*ϕ*s using hypotonic lysis, mechanical disruption, and differential centrifugation processes. 2) The sonication method was used to fuse the M*ϕ*CM on the surface of the PLGA NPs as the core.	102.0 nm −26.7 mV	Using the developed system, sepsis control was successfully achieved through a two‐step neutralization process, including endotoxin neutralization followed by cytokine sequestration. In a mouse *E. coli* bacteremia model, treatment with the PLGA NP@M*ϕ*CM reduced proinflammatory cytokine levels, prevented bacterial propagation, and enhanced the survival of infected mice.	^[^ ^141^ ^]^
QE‐loaded BS‐NPs@M*ϕ*CM	1) Hollow mesoporous BS NPs were prepared by the hydrothermal process method and then anticancer drug, QE, was encapsulated into the BS‐NPs. 2) M*ϕ*CM was isolated from freshly harvested M*ϕ*s. 3) M*ϕ*CM coating on the surface of the QE‐loaded BS‐NPs was performed by the extrusion method.	155.3 nm −19.1 mV	Prolonged blood circulation, high tumoritropic accumulation, proactive recruitment capacity from CCL2/CCR2, and *α*4/VCAM‐1 active targeting property were achieved by QE‐loaded BS‐NPs@M*ϕ*CM NPs, resulting in the inhibition of lung metastasis of breast cancer. Rapid release of QE upon NIR irradiation and obvious decrease of HSP70 (a malignancy‐specific‐overexpressed thermoresistance‐related chaperone) further enhanced the sensitivity of breast cancer to PTT. The combination of HSP70‐inhibition and p‐Akt/MMP‐9 downregulation augmented the potency of the NP in breast cancer therapy. The biomimetic nanosystem successfully employed in CT and IRT imaging.	^[^ ^142^ ^]^
EM‐loaded liposomes@M*ϕ*CM	1) pH‐sensitive EM‐loaded liposomes were prepared by a thin film‐hydration method using EM, DOPE, and DSPE‐PEG with 1:15:15 w/w ratio. 2) M*ϕ*CMs were harvested from RAW 264.7 cells by direct extrusion and centrifugation processes. 3) The decoration of EM‐loaded liposomes with M*ϕ*CM was carried out by the extrusion method to prepare EM‐loaded liposomes@M*ϕ*CM.	115.4 nm 26.2 mV	The encapsulation efficiency of EM into the liposomes was 96.7% and showed a pH‐sensitive drug release profile. EM‐loaded liposomes@M*ϕ*CM could effectively improve the cellular uptake of the drug in metastatic 4T1 breast cancer cells and exhibited high inhibitory effects on the viability of cells. The M*ϕ*CM coating effectively improved the specific targeting of nanosystem to metastases foci in the lung, and significantly inhibited lung metastasis of breast cancer.	^[^ ^123^ ^]^
Fe_3_O_4_@M*ϕ*CMs–anti‐EpCAM Ab	1) Azide‐modified M*ϕ*CMs were prepared through electrostatic interaction between Fe_3_O_4_ clusters and azide‐modified leukocyte membrane fractions using the ultrasonic method. 2) DBCO‐functionalized Ab was prepared through the reaction of NHS‐PEG_4_‐DBCO linker and anti‐EpCAM Ab. 3) Click reaction was used to conjugate the azide‐modified M*ϕ*CM with DBCO‐functionalized Ab to prepare anti‐EpCAM Ab decorated Fe_3_O_4_@M*ϕ*CMs.	≈240 nm ≈−20 mV	Anti‐EpCAM Ab decorated Fe_3_O_4_@M*ϕ*CMs displayed good colloidal stability and can be guided by a conventional magnetic field. The biomimetic immunomagnetosomes exhibited superior CTC recognition efficiency by capturing ≈90% of tumor cells from whole blood in only 15 min.	^[^ ^143^ ^]^
MNCs@M*ϕ*CM–anti‐CD28 Ab‐pMHC‐I	1) Azide‐modified M*ϕ*CM was prepared through culturing J774A.1 cells with azide‐Cho, and subsequent purification by a discontinuous sucrose density gradient before ultracentrifugation to harvest membrane fractions. 2) MNCs were electrostatically coated with the azide‐modified M*ϕ*CM to obtain MNCs@azide‐modified M*ϕ*CM. 3) pMHC‐I and anti‐CD28 Abs, the T‐cell stimulatory signals, were modified by DBCO and then conjugated to MNCs@azide‐modified M*ϕ*CM through highly efficient and mild copper‐free click reaction to obtain MNCs@M*ϕ*CM anti‐CD28 Ab‐pMHC‐I.	≈380 nm ≈−25 mV	The pMHC‐I and anti‐CD28‐functionalized MNCs@M*ϕ*CM could efficiently expand and stimulate CD8+ T cells ex vivo and guided reinfused CTLs to tumor tissues through magnetic control, allowing the MRI of the particles. MRI images showed that the biomimetic nanosystem had superior tumor infiltration, which was attributed to the stealth effect of the coated M*ϕ*CM on the surfaces of the NPs and prolonged blood circulation. In vivo studies showed that the tumor growth was delayed along with negligible systemic toxicity, demonstrating the potency of the developed system for cancer immunotherapy.	^[^ ^144^ ^]^

^a)^ Abbreviations: Ab: antibody; AbNPs: albumin nanoparticles; AuNS: gold nanoshell; azide‐Cho: azide‐choline; BR: bilirubin; BS: bismuth selenide; CCL2: C‐C chemokine ligand 2; CCR2: C‐C chemokine receptor 2; CT: computed tomography; CTC: circulating tumor cell; CTL: cytotoxic T cell; DBCO: dibenzocyclooctyne; DOPE: 1,2‐dioleoyl‐*sn*‐glycero‐3‐phoshoethanolamine; DSPE‐PEG: distearoylphosphatidylethanolamine‐poly(ethylene glycol); *E. coli: Escherichia coli*; EM: emtansine; EpCAM: epithelial cell adhesion molecule; GSH: glutathione; HMSNs: hollow mesoporous silica nanocapsules; HPMC: hollow polyelectrolyte multilayer capsule; HSP70: heat shock protein 70; IRT: infrared thermal imaging; leutusome: leukocyte and tumor cell membrane camouflaged liposome; LPS: lipopolysaccharide; MMP‐9: matrix metalloproteinase‐9; MNCs: magnetic nanoclusters; M*ϕ*CM: macrophage cell membrane; MRI: magnetic resonance imaging; NIR: near‐infrared; p‐Akt: protein kinase B; PDT: photodynamic therapy; PLGA: poly(lactic‐*co*‐glycolic acid); pMHC‐I: peptide (SIINFEKL)‐loaded major histocompatibility complex class‐I; PTT: photothermal therapy; PTX: paclitaxel; PTX2‐TK: thioketal‐linked PTX dimer; QE: quercetin; TC: T cell; UCNPs: upconversion nanoparticles; VCAM‐1: vascular cell adhesion molecule‐1.

For example, Visceral Leishmania, a deleterious tropical disease caused by an intracellular parasite, has poor treatment outcomes as a result of parasite resistance against the first therapeutic line.^[^ ^145^ ^]^ Based on the fact that promastigotes recognize the M*ϕ*s surface proteins, amphotericin‐B‐loaded vesicles of the M*ϕ*CM were used to deceive and destroy the parasite.^[^ ^146^ ^]^ First, the blood MCs were differentiated to M*ϕ*s and their membranes were extracted. It was confirmed that the M*ϕ*s’ surface proteins keep their functionality including on the surface of the vesicles. Compared to the administration of the free drug and the commercial liposomal formulation of the drug, AmBiosome, the developed leukolike system exerted far greater toxicity against promastigotes and intracellular amastigotes in vitro. It was observed that the developed formulation induced more secretion of inflammatory cytokines, IL‐2 and IFN‐*γ*, and further activation of the host immune cells, the process that is usually suppressed by the parasites. It was confirmed that the leukolike formulation led to more production of reactive oxygen and nitrogen species for eliminating intracellular parasites. Interestingly, it was observed that the infected M*ϕ*s attracted leukolike vesicles more than the uninfected M*ϕ*s, implying the specific targeting of the developed formulation. Finally, the safety of the leukolike formulation of amphotericin‐B was compared with its free form and it was demonstrated that healthy mononuclear cells tolerated the leukolike formulation better than the free form.

To date, because of the merits of the porous silicon and silica NPs for drug delivery, many attempts have been made to improve their physicochemical properties, as well as their behavior in biological conditions.^[^ ^49^, ^147^ ^]^ Various types of silicon NPs regarding their payload, porosity, particle size, hydrophobicity, zeta potential, and being intact or conjugated with other materials have been covered with M*ϕ*CM for treating a variety of conditions including autoimmune diseases, cancers, and infections.^[^ ^135^, ^136^, ^137^, ^148^ ^]^ In a novel study, Fontana et al. reported bioengineering of surface‐modified porous silicon NPs for the treatment of rheumatoid arthritis.^[^ ^148^ ^]^ In the first step, three groups of particles were selected to be bioengineered: undecylenic acid‐modified thermally hydrocarbonized porous silicon NPs (UnTHCPSi NPs; negatively charged, hydrophobic), (3‐aminopropyl)‐triethoxysilane modified thermally carbonized porous silicon NPs (APTS‐TCPSi NPs; positively charged), and thermally carbonized porous silicon NPs (TCPSi NPs; negatively charged, hydrophilic). Then, to cover the NPs with KG‐1 M*ϕ*s’ membrane, an extrusion method using Milli‐Q water or sucrose 0.3 m mediums were used. The results showed that the membrane envelope on the APTS‐TCPSi NPs was not complete and hence, they tended to aggregate. On the other hand, the other two types of NPs were completely encapsulated in the M*ϕ*CM, while the TCPSi NPs revealed to hold about twofold more phosphatidylcholine on their surface in comparison with the UnTHCPSi NPs. As the APTS‐TCPSi NPs had the least stability in PBS, further biological evaluations were focused on the other two NPs in their coated and uncoated forms. In vitro studies investigating the possibility of systemic administration and intra‐articular administration of the NPs, demonstrated that in the plasma and the simulated synovial fluid, coated and uncoated TCPSi NPs had the same stability, whereas the coated UnTHCPS NPs had significantly more stability compared to their uncoated counterparts. The immunogenicity evaluations also revealed that unlike the uncoated UnTHCPS NPs, their coated counterparts as well as the coated and uncoated TCPSi NPs did not induce CD80 expression in the KG‐1 M*ϕ*s after 48 h of cotreatment. In conclusion, the developed biomimetic membrane coated platforms were suggested for drug delivery to induce immune tolerance against autoimmune disorders like rheumatoid arthritis.

**Figure 15:**
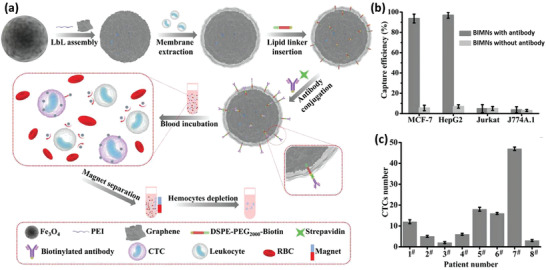
a) Scheme of fabricating leukocyte‐mimicking immunomagnetic nanoplatform and its potential for CTCs isolation from blood samples. b) In vitro capture efficiency of Ab modified and nonmodified BIMNs toward MCF‐7 and HepG2 cells as two antigen‐positive cells as well as Jurkat and J774A.1 as two antigen‐negative cells. c) CTCs enumeration in 1.5 mL blood of eight cancer patients. All bars represent means ± SD (*n* = 3). Reproduced with permission.^[^ ^150^ ^]^ Copyright 2019, Wiley‐VCH.

Metallic NPs are another group of nanomaterials reported to be camouflaged with M*ϕ*CM.^[^ ^134^, ^142^, ^149^ ^]^ Iron oxide NPs, whether alone or in conjugation with other materials, have been harnessed based on their capabilities of magnetic guidance, imaging, and PTT for diagnosis and treatment of cancer.^[^ ^138^, ^139^, ^143^, ^144^, ^150^ ^]^ For instance, in a very recent attempt, Zhou et al. have introduced an immunomagnetic nanoplatform for the detection and isolation of CTCs.^[^ ^150^ ^]^ Through a layer‐by‐layer assembly technique, polyethyleneimine and graphene nanosheets were covered on the negatively charged Fe_3_O_4_ NPs. Interestingly, due to the strong dispersion force between graphene nanosheets and lipid molecules of the cell membrane, NPs could be enveloped within the M*ϕ*CMs simply via incubation. Next, a biotin‐conjugated lipid linker was designed and inserted in the membrane, so that specific Abs could be attached on the surface via streptavidin–biotin interactions. The procedure of fabricating these nanoplatforms is reflected in **Figure** **15**a. After being surface‐modified with the anti‐EpCAM Abs, specificity and sensitivity of the developed platform for isolating tumor cells were studied. HepG2 hepatocellular carcinoma cells and MCF‐7 breast cancer cells both expressing EpCAM, as well as Jurkat TCs and J774A.1 M*ϕ*s as antigen free controls were chosen for the capturing efficiency analysis. It was observed that biomimetic immunomagnetic nanoparticles (BIMNs) modified with Abs could strongly attach to cancer cells and isolate them, while this behavior was not observed for the TCs and M*ϕ*s (Figure 15b). Moreover, the BIMNs without Abs could not even capture cancer cells. The capturing efficiency for antigen‐positive cells was dependent on the NPs’ concentration between 25 and 125 µg mL^−1^, as well as the incubation time between 20 and 90 s. The developed Abs‐modified BIMNs also had a better sensitivity and specificity compared to a commercial formulation, MACS beads. Further, the viability and proliferation of the isolated cancer cells showed no significant difference with the control group. Finally, to assess the reliability and reproducibility of the isolation procedure with the developed nanoplatforms, BIMNs were applied to blood samples from five healthy volunteers and eight patients with epithelial cancers. As shown in Figure 15c, 2 to 48 CTC were detected in 1.5 mL of blood from cancer patients, while healthy samples had no CTC. Besides, good reproducibility (mean RSD 8.7 ± 5.6%) was observed by the enumeration of CTCs in the same blood samples, indicating that Abs‐modified BIMNs are promising for clinical translation.

**Figure 16:**
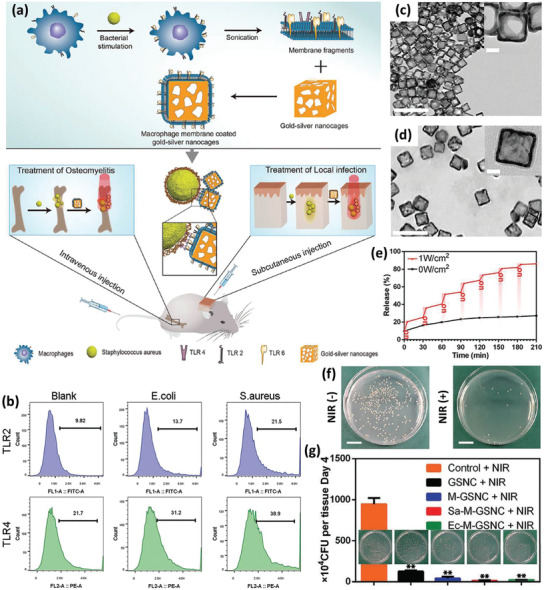
a) M*ϕ*CM enveloped gold–silver nanocages intended for drug delivery and PTT of localized infection. b) Flow cytometry characterization of M*ϕ*s pretreated with bacteria, showing high expressions of TLR2 and TLR4 in M*ϕ*s. c) TEM image of nanocages before coating with the membrane of M*ϕ*s pretreated with *S. aureus*. d) TEM image of nanocages after coating with the membrane of M*ϕ*s pretreated with *S. aureus*. Scale bar is 200 nm. Inserted image scale bar is 20 nm. e) The pattern of drug release from the enveloped nanocages with and without NIR irradiation. f) Antibacterial efficiency of enveloped NPs with or without NIR irradiation (scale bar is 2 cm). g) Quantitative analysis and photographs of the bacterial colony of the infected tissues treated differently by NIR irradiation (808 nm, 1.0 W cm^−2^, 5 min). GSNC, M‐GSNC, Sa‐M‐GSNC, and Ec‐M‐GSNC refer to gold–silver nanocage, M*ϕ*CM enveloped gold–silver nanocages, M*ϕ* membrane enveloped gold–silver nanocages where the membrane was pretreated with *S. aureus*, and M*ϕ*CM enveloped gold–silver nanocages where the membrane was pretreated with *E. coli*, respectively. Adapted with permission.^[^ ^149^ ^]^ Copyright 2018, Wiley‐VCH.

In addition to cancer therapy and cancer diagnosis, M*ϕ*CM coated NPs can be used for antibacterial purposes. Wang et al. have introduced a nanoplatform based on the gold–silver nanocages with antibacterial properties and the potential of being loaded with antimicrobial agents to target complicated bacterial infections via a mechanism inspired by M*ϕ* bacterial recognition ability.^[^ ^149^ ^]^ To this end, M*ϕ*s were first pulsed with *Staphylococcus aureus* to overexpress recognition patterns (pathogen‐related receptors, TLR2, TLR4, TLR6). Then their cell membranes were extracted and coated on the gold–silver nanocages by extrusion for further usage as NIR mediated photothermal agent for the treatment of local infections or osteomyelitis (**Figure** **16**a). The initial hypothesis of the work was proved by treating M*ϕ*s with *S. aureus* and *E. coli* as model Gram‐positive and Gram‐negative bacteria, respectively, and confirming the upregulation of pathogen‐related receptors on the M*ϕ*s (Figure 16b). The flow cytometry results demonstrated stronger TLR2 and TLR4 expression by *S. aureus* as compared to the cells treated with *E. coli*. The activated cell membrane was then coated on the nanocages to form a 3 nm lipid–protein layer as shown in the TEM images (Figure 16c,d). The enveloped nanocages revealed considerable stability in PBS, maintained their NIR absorbance at around 800 nm for being exploited in PTT, and their photothermal stability after 7 radiation cycles remained unchanged. Methylene blue, as a model antimicrobial drug, was loaded in the developed platform and its NIR‐triggered release was observed in vitro (Figure 16e). Membrane‐coated nanocages had significant interaction with the suspended bacteria and it was observed that NIR radiation completely inhibited bacterial growth in the plates containing enveloped nanocages, while in the absence of NIR radiation bacterial growth was significant (Figure 16f). Further, the antibacterial effect of the developed platform was investigated in two mouse models of localized subcutaneous infection and osteomyelitis induced by *S. aureus*. It was observed that enveloped nanocages, when injected subcutaneously or intravenously, have a remarkably higher presence in the infection site and circulation than that of their naked counterparts. Figure 16g clearly shows the results of s.c. infection site that has received NIR irradiation after different treatments.

**Figure 17:**
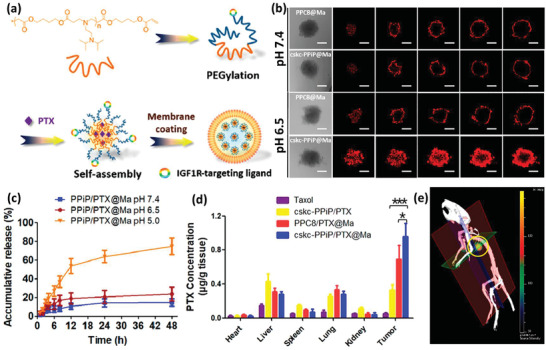
a) Illustration of the preparation procedure of the membrane‐coated nanoparticles for targeted and on‐demand drug release. b) Penetration efficiency of PPC8@Ma and cskc‐PPiP@Ma into tumor spheroids with 2 h of incubation in culturing media with two different pH values of 7.4 and 6.5. Scale bar is 100 µm and the *z*‐axis depth is 20 µm. c) Cumulative drug‐release profile of PPiP/PTX@Ma at various pH values of 7.4, 6.5, and 5, mimicking representatives of the physiologic, tumor microenvironment, and endosomal pH, respectively. d) Quantification of PTX concentration in tumor tissue and different organs of mice after receiving commercial Taxol or different formulations. e) 3D reconstruction of fluorescence signal accumulation in the mouse body 48 h after treatment with cskc‐PPiP@Ma. Reproduced with permission.^[^ ^151^ ^]^ Copyright 2018, American Chemical Society.

Lipid‐based NPs and polymeric NPs have also been reported to be coated with M*ϕ*CM for biomimicry purposes.^[^ ^123^, ^141^, ^151^ ^]^ For instance, a group of researchers has reported designing a biomimetic and pH‐sensitive system for recruitment in the tumor tissue and payload release just at the point of internalization by tumor cells. To this end, a pH‐sensitive poly(*β*‐amino ester) underwent double end PEGylation to be amphiphilic, and then functionalized with a cationic 2‐aminoethyldiisopropyl group (PPiP) to adjust the systems’ buffering capacity to the pH of the tumor microenvironment. Next, a D‐form oligopeptide (cskc) with affinity to insulin‐like growth factor 1 receptor (IGF1R), which is highly expressed on tumor cells, was attached to the PPiP (cskc‐PPiP) for facilitating the NPs’ tumor cell internalization. In the presence of PTX, self‐assembly of the polymeric chain was induced to form cskc‐PPiP/PTX that were further encapsulated into the M*ϕ*CM to create cskc‐PPiP/PTX@Ma (**Figure** **17**a). Extrusion technique was used for M*ϕ*CM coating to increase tumor recruitment while evading the RES. Another polymer with the same carbon atom numbers containing octyl group side chains but lacking pH sensitivity (PPC8) was also developed to be compared with the pH‐sensitive system by producing PPC8/PTX@Ma. The enveloped cskc‐PPiP NPs had spherical shape at pH 7.4 but disrupted at the pH value of 6.5, while the enveloped PPC8 NPs maintained their spherical shape in neutral and acidic conditions. An In vitro penetration experiment was performed on tumor spheroids at two different pH values (Figure 17b). In the blood‐mimicking milieu (pH 7.4), both PPC8@Ma and cskc‐PPiP@Ma did not show any NP penetration and only surface adsorption on the tumor spheroids was observed because their size limited infiltration into tight intercellular junctions. In contrast, cskc‐PPiP@Ma demonstrated significant penetration efficiency in the tumor‐tissue microenvironment‐mimicking milieu (pH 6.5) because the diameter of cskc‐PPiP NPs was slightly increased and the membrane disruption and exposure of the targeted internal NPs to the cells were occurred. On the other hand, PPC8@Ma maintained membrane integration and were adsorbed exclusively on the spheroid surface. In addition, being faced with a more acidic condition in the endosome, NPs were disrupted and released their PTX content (Figure 17c). In mice bearing orthotopic breast cancer, the cskc‐PPiP/PTX@Ma, NPs had the fastest and greatest tumor accumulation (Figure 17d,e), leading to the maximum antitumor efficiency, while revealing the minimum toxicity to the internal organs compared to the Taxol, cskc‐PPiP/PTX, and PPC8/PTX@Ma formulations.^[^ ^151^ ^]^

**Figure 18:**
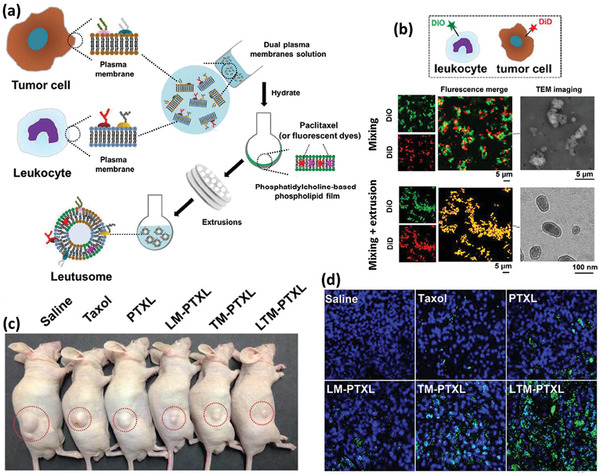
a) Scheme of the fabrication procedure of leutusomes for enhanced solid tumor targeting. b) Verification and characterizations of fusing M*ϕ* and tumor cell membranes into leutusome. Visualization of extracted dual fluorescent‐labeled composite cells membrane before and after extrusion was performed using confocal microscopy and TEM imaging. DiD was used for the labeling of leukocyte membranes (green fluorescence) and DiO was used for the labeling of tumor cell membranes (red fluorescence). c) Antitumor effect of the different formulations. PTXL refers to PTX‐loaded liposomes without any cell membrane fusion. d) Tumor tissue apoptosis induction after treatment with different formulations. Green fluorescence shows apoptotic regions. Reproduced with permission.^[^ ^140^ ^]^ Copyright 2018, American Chemical Society.

Although M*ϕ* coating can increase the homing of the NPs into tumor tissues, He et al. have introduced a novel strategy for even higher augmentation of the particles into the tumor.^[^ ^140^ ^]^ As schemed in **Figure** **18**a, they fabricated liposomes established on synthetic phosphatidylcholine‐based phospholipids and incorporated membrane fragments from both J774A.1 M*ϕ*s and HN12 head and neck carcinoma cells in the lipid bilayer via extrusion. PTX was also encapsulated within the NPs before the extrusion process to form the leukocyte and tumor cell membrane camouflaged liposome (leutusomes). To monitor the presence of both cell membrane components, the leukocyte membrane (L‐membrane) and tumor cell membrane (T‐membrane) were labeled with fluorescent dyes DiO (green) and DiD (red), respectively. The cell membranes were not spontaneously fused into nanoscale vesicles until they underwent an extrusion series after sonication (Figure 18b). The fluorescence images showed that the two cell membranes largely remained separate before extrusion, while the merged yellow color was observed after extrusion. The TEM images demonstrated that they aggregate and exhibit irregular microscale shapes before extrusion and nanoscale vesicles after extrusion. This observation confirmed that extrusion is needed to fuse two membrane fragments into nanoscale vehicles. The NPs were co‐incubated with the M*ϕ*s‐tumor cells coculture to study the effect of the liposomal composition on its uptake. It was revealed that the leutusomes have increased tumor cell uptake similar to that of the tumor cell membrane fused PTX‐loaded liposomes (TM‐PTXLs), and the reduced M*ϕ* uptake was similar to that of the M*ϕ*CM fused PTX‐loaded liposomes (LM‐PTXLs). Besides, it was observed that PTX‐loaded leutusomes (LTM‐PTXL) could significantly reduce the IC50 of PTX, specifically compared to the commercial Taxol. Next, after the i.v. injection of the formulations to the mice in 6 doses given every 4 days, it was revealed that LM‐PTXL and the LTM‐PTXL underwent significantly limited clearance due to their leukomimetic properties. On the other hand, TM‐PTXL and the LTM‐PTXL had dramatically increased tumor accumulation in a xenograft model of head and neck carcinoma, leading to less extensive biodistribution in the other organs. Ultimately, the prominent antitumor efficiency of LTM‐PTXL was confirmed with the more inhibitory effects on tumor growth as well as extensive tumor cells’ apoptosis (Figure 18c,d). As a conclusion derived from all the above‐mentioned reported formulations for treating cancers, it is clear that being enveloped with M*ϕ*CM enhanced the tumor recruitment of the NPs compared to their naked counterparts, and this issue directly resulted in high antitumor efficiency.

MCs’ cell membrane (MCM) can also be deployed for simply enveloping therapeutics or being coated on different nanoparticulate DDSs.^[^ ^152^ ^]^ A group of researchers has proposed a method for producing membrane vesicles with a higher yield than the natural rate of Exo production in the MCs.^[^ ^153^ ^]^ They placed filters with different pore sizes in the spin cups, loaded the cell suspension above the filters, and centrifuged the spin cup to force the cells through the filters and generated vesicles of the desired size. The procedure could be repeated with filters of smaller sizes when smaller vesicles are required. Finally, a size‐exclusion chromatography column could be applied to purify the developed vesicles. With this method, cell‐derived nanovesicles (CDNs) with a diameter of 50–200 nm were fabricated. Importantly, the CDNs had a 15‐fold higher protein concentration compared to the naturally secreted Exos being isolated via centrifugation. The speed of CDN production was also more than that of the Exos, while the Exos have a relatively smaller diameter and less positive surface potential. In vitro studies showed that various mechanisms were involved in CDN cellular uptake, while the main mechanism was clathrin‐mediated endocytosis. In vivo biodistribution studies also revealed significant tumor accumulation of CDNs, while the liver was the main site of accumulation. In the next step, four methods were investigated for DOX loading into the CDNs, including 1) incubation at 37 °C for 5 min, 2) incubation in the presence of saponin for 5 min, 3) incubation at room temperature for 24 h, and 4) three freeze–thaw cycles. It was revealed that the best results in terms of high drug loading efficiency and a modest particle size increment were associated with incubation in the presence of saponin and incubation at 37 °C for 5 min. Therefore, the vesicles being loaded with these two methods were selected for the next evaluations. Release studies showed that both of the selected formulations released less than 50% of their drug content within the first 36 h, while those from the saponin‐treated group revealed even less drug leakage. Further, cellular uptake studies and cell viability tests confirmed the preferential tumor‐targeting provided by the CDNs compared to the free DOX.^[^ ^154^ ^]^

In another study, Krishnamurthy et al. proved that the *α*4*β*1 integrin, necessary for MC adhesion to the vascular cell adhesion molecule 1 (VCAM‐1) on the breast tumor cells was well preserved on the MCM after PLGA NP coating via extrusion.^[^ ^152b^ ^]^ The enveloped particles had a diameter of ≈177–197 nm, a zeta‐potential of −16.5 mV, good serum stability within 120 h, high drug loading capacity, and sustained drug release over 72 h. In vitro analyses revealed that the tumor cells took up enveloped NPs more than the naked PLGA NPs with a *α*4*β*1 integrin‐dependent mechanism. The enveloped NPs were found to have a threefold lower IC50 for the breast tumor cells compared to the bare NPs. Hollow polymeric capsules covered with MCM has also been introduced for selective cancer PTT.^[^ ^155^ ^]^ To fabricate these particles, PAH and polystyrene sulfonate (PSS) were coated via layer‐by‐layer assembly technique on silica NPs. Next, gold NPs were adsorbed on top of the polymeric layer and the silicon NPs template was solubilized to form hollow capsules. In the next step, the AuNS was modified with methoxy‐omega‐mercapto‐polyethylene glycol (HS‐PEG) and then the NPs were incubated with the fragments of MCM. The final particles had a diameter of 5 ± 0.5 µm, a zeta‐potential of −26.4 ± 2.14 mV, and good stability in PBS and cellular media for 24 h. In a coculture of the tumor and nontumor cells, it was demonstrated that the microparticles only attach to the tumor cells. Further, an NIR laser with the wavelength of 808 nm could destroy the tumor cells that were incubated with the developed microparticles.

In conclusion, as it was implied by the results of the above‐mentioned studies, M*ϕ*s and MCs’ membrane brings at least two important advantages for the DDSs. First, because of the leukocyte‐specific molecules, they have minimal interactions with the body's leukocytes and provide prolonged circulation of the biomimetic platforms. Second, because of expressing the recognition patterns for specific inflammatory conditions like bacterial infection or tumors, they can provide targeted delivery of the developed systems. Harnessing these properties, promising platforms could be developed for various applications like drug delivery, PTT and PDT, blood detoxification, cell isolation, and imaging.^[^ ^122b^ ^]^

### 4.3 Neutrophil Cell Membrane for Biomimetic Drug and NP Delivery

Due to the abundant number of NEs in circulation, their extraction for preparing biohybrid materials is quite facile. Several techniques based on cell lysis and serial centrifugation is available for isolating NEs’ membrane.^[^ ^156^ ^]^ Furthermore, a nitrogen cavitation method has been proposed to disrupt living NEs by generating a mechanical force, leading to the spontaneous formation of membrane‐based vesicles.^[^ ^157^ ^]^ The most important issue regarding the cell membrane's isolation is to assure the maintained functionality of key molecules like specific receptors on the extracted membrane. It has been proved that nitrogen cavitation is more reliable for maintaining the functionality of surface proteins compared to cell lysis techniques because they quickly disrupt the cell membrane.^[^ ^66^ ^]^ Based on the membrane composition of the NEs, biomimetic NPs and microparticles enveloped with the NEs’ membrane have the potential of targeting various conditions like acute inflammations, localized tumors, tumor metastasis, and sepsis.^[^ ^66^, ^74^, ^122^, ^158^ ^]^

**Figure 19:**
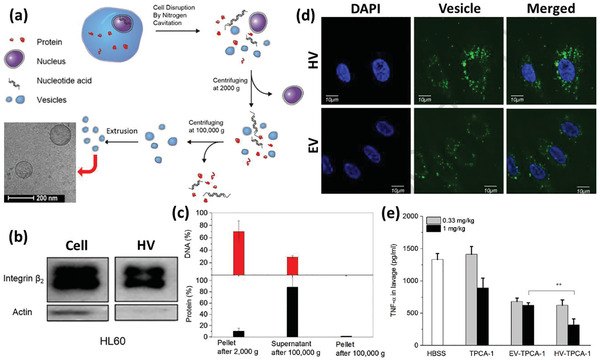
a) Schematical represention of the process of HV generation using the cell membrane of HL 60 NEs and cryo‐TEM of the final product. b) Western blot of HL 60 cells and their HVs to assess the expression of integrin *β*2 and actin. Integrin *β*2 on HVs promotes their binding to ICAM‐1 overexpressed HUVECs and their internalization. c) Quantification of DNA and proteins in each step of centrifugation. The pellet after centrifugation at 2000 × *g* contained nucleus. The supernatant of the samples after centrifugation at 100 000 × *g* contained intracellular proteins and released DNA, while its pellet was the final HVs that had very low amount of DNA and protein. d) Fluorescence confocal images of HUVECs after incubation with Dil‐fluorescently labeled HVs or EVs. HUVECs were treated with 100 ng mL^−1^ of TNF‐*α* to increase the expression of ICAM‐1. HVs could attach and penetrate into the cells more effectively than EVs due to the surface integrin *β*2 on the HVs. e) HV‐TPCA‐1 could show the highest attenuation of vascular inflammation in vivo, confirmed by quantification of the TNF‐*α* concentration in BALF 10 h after i.v. injection of Hank's balanced salt solution (HBSS), TPCA‐1‐loaded EVs (EV‐TPCA‐1), and HV‐TPCA‐1 in mice 3 h after LPS challenge (10 mg kg^−1^). The administered doses of TPCA‐1 were 0.33 and 1 mg kg^−1^. Reproduced with permission.^[^ ^159^ ^]^ Copyright 2016, Elsevier.

To address vascular inflammation, a group of researchers developed a NE membrane‐based DDS being prepared via a novel nitrogen cavitation method.^[^ ^159^ ^]^ To assure the targeting potential of the membrane vesicles, first the NE‐like cells (HL60 cell line) were primed with dimethyl sulfoxide (DMSO) to express *β*2 integrins and then the cells were disrupted in a nitrogen cavitation vessel under the pressure of about 350 psi for 20 min. With two centrifugation steps (2000 × *g* and 100 000 × *g*), the membrane vesicles were extracted and then extruded to form uniform NPs with a diameter of 200 nm and PdI of 0.25 (**Figure** **19**a). The surface potential of HL60 cell membrane‐formed nanovesicles (HVs) was about −16 mV and they contained ≈50% w/w protein and ≈50% lipids, which was consistent with a plasma membrane composition. The pharmaceutical stability of their lyophilized form revealed that the NPs had a 10% increase in diameter after 6 days storage in 4 °C, and when 4% of sorbitol was added to the formulation, reconstituted nanovesicles had a dramatic increase in size that could be reversed to the initial 200 nm by sonication. Sodium dodecyl sulfate polyacrylamide gel electrophoresis (SDS‐PAGE) analysis showed a similar protein pattern as the HV nanovesicles HL 60 cell lysate for integrin *β*2, which is expressed on the cell membrane surface. In contrast, actin (an intracellular protein) was not detected in HV nanovesicles at the same loading amount of samples, confirming the successful separation of HVs (Figure 19b). The amount of deoxyribo nucleic acid (DNA) and total protein were also remarkably diminished in the pellet after the final step of centrifugation at 100 000 × *g* (Figure 19c). This observation demonstrated that the DNA and intracellular proteins were not available in the purified HV sample. To mitigate the vascular inflammation, TPCA‐1, an inhibitor of the NF‐*κ*B signaling pathway, was loaded within the NPs. Furthermore, to evaluate the nanovesicles binding to the vascular cells, they were incubated with human umbilical vein endothelial cells (HUVECs) and significant HV uptake (threefold increase) compared to the erythrocyte vesicles (EVs) was observed (Figure 19d) as a result of the interaction of *β*2 integrins of HVs with highly expressed ICAM‐1 proteins on the surface of the HUVECs mediated by TNF‐*α*. The same mechanism was also confirmed to be the key reason for the HV attachment to the activated endothelium in vivo. It was observed that the HVs accumulated adjacent to the native NEs in the inflamed vasculature, but did not interact with them. When administered in a mouse model of lung vasculature inflammation, it was observed that the accumulation of the HVs was increased 5–10 times compared to the healthy animals. Moreover, it was observed that the developed formulation could significantly reduce the expression of ICAM‐1 in the inflamed lung vasculature, which is responsible for NE infiltration to the inflammation site and exacerbating the inflammatory condition. Finally, significantly diminished concentration of IL‐6 and TNF‐*α* in the bronchoalveolar lavage fluid (BALF) after the administration of TPCA‐1‐loaded HVs (HV‐TPCA‐1) implied the mitigated inflammatory response, in comparison to the other tested groups (Figure 19e).

In a further step, piceatannol (another inhibitor of the NF‐*κ*B signaling pathway) was loaded into the same developed vesicles to treat acute lung inflammation. Importantly, drug loading was performed by generating a pH gradient between the inner cavity of nanovesicles and their surrounding environment. Disrupting the NEs in a buffer with pH 9 led to basification inside the membrane vesicles and increased the drug loading of the piceatannol, which is a weak acid. It was observed that the drug‐loaded nanovesicles could decrease the ICAM‐1 expression on endothelial cells in vitro. Intravenous administration of the developed formulation in a mouse model of acute lung inflammation led to a significant decrease in leukocytes’ infiltration into the lung tissue, reduced concentration of IL‐6 and TNF‐*α*, and even the reversal of lung edema compared to the free piceatannol. Furthermore, in mice receiving a lethal dose of LPS as a model of sepsis, i.v. injection of drug‐loaded nanovesicles prevented an increase in inflammatory cytokines, NE infiltration in lung, kidney, and liver, and specifically reduced the death incidence by 80%, while administration of the free piceatannol could not increase mice survival.^[^ ^160^ ^]^ In another study, the same developed vesicles were used to deliver resolvin D2 to the ischemic brain for preventing ischemia/reperfusion injury. First, the nanovesicles were formed through nitrogen cavitation of mature NEs and then extruded to form uni‐size particles of 190 nm with a PdI of 0.21. After being loaded with the resolvin D2, the zeta potential of the NPs changed from −10.1 to −14 mV, stating that the resolvin molecules might be placed within the lipid bilayer. The particles had good stability in a solution with 20% serum and in a release study, liberated 80% of their drug content within 24 h. After it was confirmed that the proteins that are required for the attachment of NEs to the inflamed vasculature are present on the surface of the nanovesicles, the formulation was tested in vivo on a mouse model of ischemia/reperfusion injury. It was demonstrated that the resolvin D2‐loaded NE‐based nanovesicles could specifically target and accumulate in the injury site and therefore increase drug delivery rate. Besides, a significant reduction of the ICAM‐1 expression on damaged endothelial cells, as well as a tenfold decrease in the NEs’ infiltration to the injured lesion led to the inhibition of secondary damage after stroke generated by NEs’ inflammatory response. Histological studies finally confirmed a meaningful reduction in infarcted volume in the mice receiving the developed nanoformulation compared to the control group receiving PBS buffer.^[^ ^161^ ^]^

Due to the NEs’ key role in inflammatory disorders like arthritis, it was hypothesized that polymeric NPs wrapped in NEs’ membrane could alleviate synovial inflammation and joint damage by mimicking the NEs’ and thus inhibiting their activity. To this end, PLGA NPs were cloaked within the membrane of blood NEs and the resulting biomimetic particles had a diameter of 90–150 nm, a zeta potential of about −17 mV, a spherical core–shell structure and maintained the key protein composition of the NEs’ membrane with a right side‐out orientation. Like NEs, the NE‐NPs could also attach to the inflamed cells (HUVEC and chondrocytes), probably via the interaction of LFA‐1 on the membrane of NEs and ICAM‐1 of inflamed cells in vitro. It was observed that NE‐NPs could inhibit chondrocyte activation and apoptosis induced by IL1‐*β* and TNF‐*α* in a dose‐dependent manner. In the co‐incubation of chondrocytes with synovial fluids from patients with rheumatoid arthritis, it was also observed that NE‐NPs could neutralize proarthritogenic factors and inhibit the activation of chondrocytes. Next, it was revealed that in a mouse femoral head explant being treated with inflammatory cytokines, NE‐NPs could deeply penetrate the injured cartilage and be taken up by activated chondrocytes. Furthermore, NE‐NPs could significantly prevent cartilage matrix loss compared to the control group. In line with the in vitro results, in vivo studies further unraveled the NE‐NPs potential to prevent and treat joint destruction through chondral protection and inhibition of CD248‐positive and fibronectin‐positive fibroblasts that exacerbate the inflammatory condition in the joint.^[^ ^162^ ^]^ PLGA‐coated NPs with the NE membrane have also been utilized to target circulating tumor cells for preventing metastasis. To fabricate the NPs, the membrane from NEs was first pretreated and activated with LPS, and then, extracted and assembled into vesicles. In the next step, Carfilzomib‐loaded PLGA NPs were cosonicated with the membrane vesicle to generate the membrane coated NPs based on the electrostatic interactions between the polymer and the membrane. The NE‐NPs were spherical core–shell structures, with an average diameter and zeta potential of about 95 nm and −35 mV, respectively. The membrane coating revealed to increase the particle's colloidal stability in both PBS and serum‐containing PBS. After the confirmation of presence and functionality of NEs’ adhesion molecules (like LFA‐1) on the surface of the NE‐NPs, the specific targeting potential of the NE‐NPs for breast cancer cells was evaluated both in static and sheared conditions in vitro and revealed significant dependence to ICAM‐1 and CD44 overexpression on the tumor cells. It was observed that the NE‐NPs could attach to the inflamed endothelial cells under the shear stress associated with the blood circulation. Biodistribution studies revealed that NE‐NPs target and recruit in the breast cancer lung metastatic foci 2.12 times more than bare NPs 24 h after i.v. injection. Besides, the Carfilzomib‐loaded NE‐NPs could significantly decrease lung metastatic foci compared to the bare NPs and the free drug.^[^ ^124^ ^]^ Despite exploiting activated NEs’ membrane in the previous studies, another nanoformulation has been explored to target inflammation in pancreatic carcinoma taking advantage of naïve NEs’ membrane. First, blood NEs were collected and turned into NE membrane vesicles, and then they were extruded with celastrol‐loaded PEG‐PLGA NPs to form the core–shell NE‐NPs with a size of ≈167 nm diameter and a ≈−15 mV zeta potential. The proteomic analysis further approved that the protein composition of the NPs’ envelope was similar to the NEs’ membrane and the drug release analysis revealed a biphasic pattern with <50% liberation within the first 5 h. NE‐NPs had a greater uptake efficiency compared to the bare NPs in HUVEC and Panc02 cellosaurus cancer cells, but not in not‐stimulated M*ϕ*s. Due to the increased internalization efficiency in Panc02 cells, NE‐NPs could diminish IC50 and induced more apoptosis. In vivo studies in a xenograft Panc02 mouse model demonstrated the prolonged circulation, as well as greater tumor accumulation of the NE‐NPs, leading to significantly higher antitumor efficiency in comparison with the control groups. The same results were also observed in an orthotopic pancreatic cancer model, in addition to a significantly increased animal survival and meaningful inhibition of liver metastasis, while inducing no clear off‐target toxicity.^[^ ^163^ ^]^ Further, the similar celastrol‐loaded PEG‐PLGA NPs coated with the NE membrane was evaluated for its anti‐inflammatory efficiency in acute pancreatitis.^[^ ^164^ ^]^ A major difference of particles fabricated in this study compared to the previous one was the smaller diameter of the core PEG‐PLGA NPs, because using core NPs with a 150 nm diameter led to an increased NP accumulation in the inflamed pancreas. As expected, NE‐NPs had significantly more accumulation in the inflamed region compared to the bare NPs in vivo, most likely because of the chemokine gradient rather than the targeting of neovasculature. As a result of more efficient drug delivery to the inflammation site, all of the inflammation hallmarks, including the activity of pancreatic amylase, weight of ascetic fluids, pancreatic edema, and infiltration of inflammatory cells were significantly decreased. During treatment, the serum concentrations of TNF‐*α* and IL‐6 were significantly abated, implying that the developed NPs could ameliorate both local and systemic inflammation.

**Figure 20:**
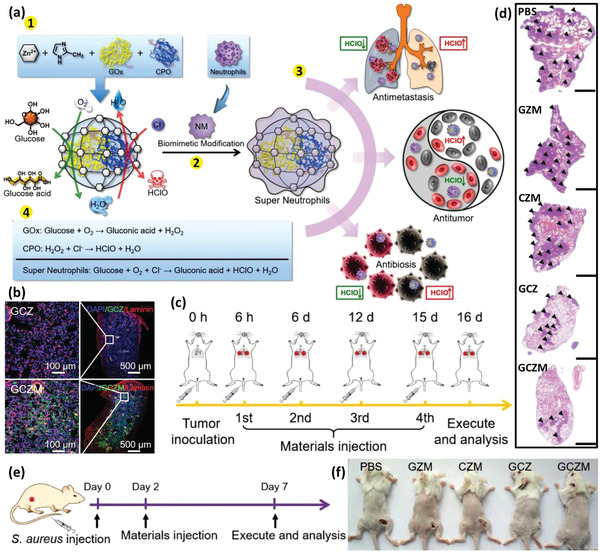
a) Schematic depiction of the fabrication, biological mechanism, and different biomedical applications of the “super NEs,” prepared by modifying the surface of the GOx/CPO‐embedded ZIF‐8 NPs with the natural NE membrane. b) Confocal microscopy analysis of the lung tissues after i.v. injection of FITC‐labeled GCZ and GCZM. Mice were intravenously injected with 4t1 cells before the administration of the NPs. c) Schematic demonstration of the establishment of early lung metastasis model and therapeutic procedure in BALB/c mice. d) The images of H&E stained lung tissues after the treatment of mice with different materials at day 16 post‐tumor inoculation. The black arrows show the metastatic nodules. Scale bar is 2 mm. e) Schematic demonstration of the timeline for the establishment of *S. aureus*‐infected mouse model and treatment schedule. f) Representative digital images of *S. aureus*‐infected mice at day 7 postinjection of the bacteria. Reproduced with permission.^[^ ^7^ ^]^ Copyright 2019, Wiley‐VCH.

Another advanced platform has recently been developed by Zhang et al. not only for mimicking the inflammation targeting potential of the NCs, but also for imitating their HClO production for antitumor and antimicrobial purposes.^[^ ^7^ ^]^ To tailor this so‐called artificially fabricated “super NE,” glucose oxidase (GOx) and chloroperoxidase (CPO) were incorporated within the microporous organometal structure of Zn^2+^/2‐methylimidazole‐composed zeolitic imidazolate framework‐8 (ZIF‐8). Next, repeated extrusion was used to cloak the membrane in highly active NEs, extracted from the blood of breast tumor‐bearing mice, to form glucose oxidase and chloroperoxidase coloaded zeolitic imidazolate framework‐8 coated with NE membrane (GCZM) NPs. GOx‐loaded ZIF‐8 (GZ), CPO‐loaded ZIF‐8 (CZ), GOx and CPO coloaded ZIF‐8 (GCZ), GOx‐loaded ZIF‐8 coated with NE membrane (GZM), and CPO‐loaded ZIF‐8 coated with NE membrane (CZM) were also prepared as the control groups. As illustrated in **Figure** **20**a, the “super NEs” would have the competence to target inflammation sites via NE membrane‐mediated protein‐receptor interactions. In the presence of endogenous H_2_O_2_ and Cl^−^, highly reactive HClO could be produced due to the efficient catalysis of CPO loaded in GCZM. In fact, the CPO was able to transform the H_2_O_2_ and the Cl^−^ into HClO. The efficiency of HClO generation could be greatly improved via biocatalytic cascades of GOx to consume glucose and provide sufficient H_2_O_2_ for the generation of HClO for antitumor and antibiosis purposes, while reducing H_2_O_2_‐involved systemic side effects. The GCZM NPs had a diameter of about 150 nm and a zeta potential of about −20 mV. They were also confirmed to maintain NEs’ adhesion molecules for inflammation targeting, including L‐selectin, C‐X‐C motif chemokine receptor 4 (CXCR4), and *β*1 integrin, on their surface. It was also confirmed that the GCZ NPs produce HClO eight times more efficiently, compared to the activated NEs, in a glucose‐dependent manner. GCZM NPs revealed a superior antitumor efficiency on breast cancer cells in vitro via depleting GSH and damaging both the DNA and mitochondria, specifically compared to the GCZ NPs that were lacking the targeting potential offered by the NEs’ membrane.

In vivo studies on a premetastatic mouse model indicated that the GCZM NPs significantly target and accumulate in premetastatic niches via being attached to the *α*SMA marker of fibroblast in the ECM, CD31 marker of neovasculature, and the F4/80 marker of resident m*ϕ*s. As shown in Figure 20b, laminin, an ECM component that is the main ligand for *β*1 integrin, could provide an essential target for tumor cells to accumulate in the lung. At 24 h postinjection of 4T1 cells, the expression of laminin was enhanced in the lung. 20 h postinjection of 4T1 cells, fluorescein isothiocyanate (FITC)‐labeled GCZ and GCZM were injected intravenously. The higher tumor homing effect of GCZM as compared to GCZ could be observed in the laminin‐abundant area of the lung by the detection of strong green fluorescence, suggesting the potential of “super NEs” to specifically bind to laminin in the premetastatic niches of the lung. After confirming the GCZM targeting to the premetastatic niche, various samples were intravenously injected four times (6 h, 6 days, 12 days, and 15 days) to mice inoculated with 4T1 cells (Figure 20c), and at day 16, the mice were euthanized for hemotoxylin and eosin (H&E) staining of lung tissues to visually observe the amounts and sizes of the premetastatic niche (Figure 20d). Among all the tested groups, the GCZM‐treated mice showed fewer and smaller premetastatic foci and less inflammation than the other groups as a result of excellent tumor targeting by the NE membrane. In addition, an *S. aureus* infected mice model was established by sc injection of the bacteria into the right flank of BALB/c female mice. Cy5.5‐labeled GCZ and GCZM particles were injected intravenously and fast targeting and accumulation of GCZM was observed at the inflamed infected tissues within 2 h using in vivo fluorescence imaging. Besides, in vivo antibiosis efficiency was monitored in *S. aureus* infected mice injected with various materials (PBS, GZM, CZM, GCZ, and GCZM) (Figure 20e). Five days postinjection of various materials, the mice were euthanized and the inflamed tissues were excised to quantify the number of bacteria. From the grown colonies, GCZM exhibited excellent bacterial inhibition ability with negligible wound size, confirmed by camera images shown in Figure 20f. Currently, most of the developed systems based on the NEs’ membrane have only taken advantage of its RES‐evading and targeting characteristics for inflammation and tumor sites. However, NEs’ membrane can be used to target different regenerative nanosystems to the damaged tissues for regenerative purposes, due to their potential for accumulation within the inflamed tissues.

### 4.4 Camouflaging Drug and/or NPs into T Cell Membrane

**Figure 21:**
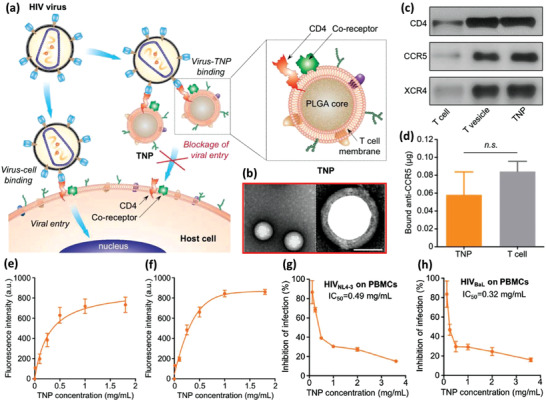
a) Schematic illustration of TNPs fabricated for reducing HIV infectivity. The cell membranes of natural CD4^+^ TCs were wrapped around polymeric cores, while preserving key coreceptors for viral targeting. By mimicking the surface antigen profile of TCs, TNPs can bind to viruses and block viral entry into the target alive cells. b) TEM images of TNPs. c) Analysis of CD4 receptor, and CCR5 and CXCR4 coreceptors related to HIV binding in TCs, T vesicle, and TNP using Western blot assay. d) Measurement of the fluorescence intensity of TNPs (100 µL, 0.5 mg mL^−1^ protein concentration) or TCs (100 µL, ≈2.5 × 10^6^ cells) comprising an identical amount of membrane content after staining with fluorescein‐isothiocyanate‐labeled anti‐CCR5 Ab. Binding capacity and specificity of TNPs against HIV envelope glycoprotein e) gp120_IIIB_ and f) gp120_BaL_. Inhibition of infection by g) HIV_NL4‐3_ and h) HIV_Bal_ on PBMCs, in response to various concentrations of TNP. Reproduced with permission.^[^ ^166^ ^]^ Copyright 2018, Wiley‐VCH.

Compared to the other immune cells, TCs express a higher level of adhesion molecules on their surface; hence, TC membranes have been widely utilized for enveloping drug‐loaded NPs to enhance their targeting efficiency.^[^ ^130^, ^165^ ^]^ A research group has reported cloaking PTX‐loaded PLGA NPs within the cytotoxic TC's membrane for targeting gastric cancer. The enveloping procedure was based on coextrusion and membrane cloaking was further confirmed with the presence of TC membrane proteins, LFA‐1 and CD3z, on the NPs’ surface as well as a core–shell structure shown in the TEM images. The NPs had a diameter of 165.9 ± 1.0 nm and revealed a biphasic drug release pattern, liberating about 35% of the drug content within the fast initial phase in the first 48 h. In vitro analyses revealed that the developed NP platform had acceptable stealth properties (23.99% less uptake by M*ϕ*s compared to the bare NPs), and was biocompatible. Intravenous administration of the membrane coated NPs had far greater accumulation in a xenograft model of gastric cancer. Moreover, low dose irradiation of the tumor before the NPs administration significantly increased the tumor localization of the NPs. After receiving 2 doses of the NPs 1 week apart, it was observed that the tumor volume in mice receiving the NPs was significantly reduced compared to those receiving free PTX.Combined radiotherapy and chemotherapy had a synergistic antitumor effect, resulting in an intense antitumor effect and even complete tumor remission in two of the subjects.^[^ ^125^ ^]^ In another study, the membrane of natural CD4^+^ helper TCs was coated on PLGA NPsʼ core for trapping and neutralizing the human immunodeficiency virus (HIV) (**Figure** **21**a). To this end, PLGA NPs were prepared via a nanoprecipitation method and cosonicated with the membrane vesicles that were prepared through combined TCs’ hypotonic lysis, mechanical disruption, and centrifugation. The resulting T‐cell‐membrane‐coated NPs (TNPs) had a diameter of 105.4 ± 4.4 nm and a zeta potential of −29.5 ± 1.2 mV, revealing a core–shell structure with a unilamellar lipid bilayer membrane in the TEM images (Figure 21b) and acceptable colloidal stability in PBS and 50% serum media. The membrane envelope of the NPs had a protein composition similar to that of the parent cells’ membrane and specifically maintained and enriched the viral receptors related to the HIVs’ cell entry, including CD4 receptor, CCR5 receptor, and CXCR4 receptor, on the surface of TNPs (Figure 21c). In addition, the coated membrane had the right‐side‐out protein orientation, confirmed by staining TNPs and TCs, containing equal amounts of membrane content, with fluorescence‐labeled anti‐CCR5 Ab (Figure 21d). After washing and removal of free Abs, both samples showed very similar fluorescence intensity. Since inside‐out membrane coating would reduce Ab staining and diminish fluorescence intensity, this observation suggested that TNPs were coated by a right‐side‐out membrane orientation. The TNPs had good specificity and binding efficiency for recombinant HIV‐1 glycoproteins, particularly gp120BaL, which represents gp120 of R5 viruses, and HIV‐1 gp120IIIB, which represents gp120 of X4 viruses (Figure 21e,f). An in vitro test on CD4^+^ cells from healthy donors also revealed that when incubated with gp120 in the presence of TNPs, their survival markedly increases compared to the control groups, because a portion of the cytopathic gp120 was neutralized by the TNPs. In another test utilizing the HIV_BaL_ and HIV_NL4‐3_, increased size and decreased zeta potential of the viruses insinuated their attachment to the TNPs, leading to a significant decline in the viruses’ infectivity and reproduction in the peripheral blood mononuclear cells. The neutralization of infectivity by X4 tropic HIV‐1_NL4‐3_ strain and R5 tropic HIV‐1_BaL_ strain was evaluated on PBMCs. To do so, 200 TCID_50_ virus was incubated with TNPs ranging from 0 to 3.6 mg mL^−1^ for 1 h before adding the mixture to 5 × 10^5^ PMBCs and culturing for 48 h. HIV p24 antigen production was measured as an indicator of HIV. In cultures without TNPs, a remarkable increase in p24 was observed as a result of viral entry and infection of the host cells, while p24 levels decreased with the increase of TNPs added to the medium, indicating a dose‐dependent neutralization effect (Figure 21g,h). The IC50 of the TNPs determined to be 0.49 and 0.32 mg mL^−1^ for inhibiting X4‐tropic HIV_NL4‐3_ and R5‐tropic HIV_BaL_, respectively, while when the TNPsʼ CCR5, CXCR4 and CD4 receptors were blocked with Abs, they exerted no inhibitory effect on HIVs’ infectivity.^[^ ^166^ ^]^ Overall, this study revealed the potential of TNPs as a novel nanosized therapeutic agent against HIV infection.

Another study has investigated a leukocyte membrane‐enveloped NP to increase the permeability of tumor vasculature. Human TC's membrane and murine M*ϕ*s were distinctly exploited to cover disc‐shape nanoporous silicon particles (NPSs), that were previously developed,^[^ ^119b^ ^]^ via electrostatic interactions between the negatively charged cell membrane and positively charged (3‐aminopropyl)triethoxysilane (APTES) on the NSPs’ surface to prepare the LLVs. Further, it was confirmed that LFA‐1 and Mac‐1 adhesive proteins were transformed to the NPs’ surface and it was observed that they have key roles in the attachment of LLVs to the endothelial cells, with LFA‐1 representing a more significant role, both in vitro and in vivo. When co‐incubated with the HUVEC activated by TNF‐*α* (a model of inflamed endothelium) under flow for 10 min, LLVs accumulated in the cell‐cell interfaces, while the bare NPSs were distributed more evenly on the cells’ surface. On the other hand, following being incubated with the LLVs, an increase in cytoplasmic Ca^2+^ and activation of protein kinase C alpha (PKC*α*) in the HUVEC implied the activation of the ICAM‐1 pathway, which involves phosphorylation of vascular endothelial cadherin (VE‐cadherin) that results in the partial disruption of the endothelial intercellular junction. It was also demonstrated that the expression of the VE‐cadherin significantly diminished in HUVEC after being incubated with the LLVs or the leukocytes (as positive control), but not with the NPSs. Furthermore, in vivo studies confirmed that LLVs targeted and bound to the tumor vasculature more firmly, detached less easily, and finally led to significantly increased extravasation of the fluorescent marker to the subendothelial space compared to the NPSs.^[^ ^119c^ ^]^ Besides taking advantage of the whole‐cell membrane for biomimicry, exploiting extracted membrane proteins is also trending. In this regard, Corbo et al. have reported a comparison between three lipid nanovesicles for treating inflammatory bowel disease; liposomes consisted of phospholipids and cholesterol, liposomes integrated with membrane proteins from unstimulated TCs (LKs), and liposomes integrated with the membrane proteins from TCs that had been incubated with retinoic acid to overexpress *α*4*β*7 integrin (SLKs). Liposomes, LKs, and SLKs had an average diameter of 166, 164, and 147 nm and a zeta potential of −36.3, −33.2, and −32.4, respectively.^[^ ^167^ ^]^ In vivo biodistribution studies on a mouse model of colon inflammation revealed that liposomes following a passive targeting mechanism, and LKs and SLKs following an active targeting mechanism, have similar accumulation efficiencies in the inflamed colon, although they had different localization patterns. Liposomes were placed in the interstitial space, while LKs and SLKs revealed a higher tendency to the vasculature. Histological studies and gene expression analysis of the inflamed colon revealed that SLKs exerted the maximum reduction in leukocyte infiltration and tissue normalization, and significantly reduced the expression of IL‐6 and TNF‐*α* and increased the expression of Mannose receptor C type 1 (MRC‐1), while showing a decreased serum concentration of TNF‐*α*. Altogether, the results indicated the superior localization and systemic anti‐inflammatory effects of the SLKs.

### 4.5 The Membrane of NKCs for Biomimetic Drug/NP Delivery

NKCs with significant antitumor activity via generating perforin and granzyme, have been reported to express receptors for targeting specific ligands on the tumor cells’ surface.^[^ ^168^ ^]^ This fact has been an underlying reason for exploiting NKCs’ membrane to develop biomimetic drug delivery systems with improved tumor targeting and recruiting. Pitchaimani et al. reported extracting activated NKCs’ membrane via hypotonic lysis and centrifugation and incorporating the membrane fragments in fusogenic CLs through extrusion to form NKsomes.^[^ ^169^ ^]^ Evaluating various protein/lipid ratios for the formulation of NKsomes, it was revealed that a 1:500 weight ratio provides the optimum colloidal stability. The resulted NKsomes contained the key targeting proteins of the parent cells (CD56, natural killer group 2 member D (NKG‐2D), natural killer receptor P30 (NKp30)), had an average diameter of 88 ± 1 nm, and a zeta potential of 14 ± 0.3 mV. The optimized NKsomes had a DOX loading efficiency comparable to that of the simple CLs, while revealing a significant sustained drug release both in the physiological and acidic pH, associated with the tumor microenvironment. The ability of the NKsomes to target the tumor cells was then confirmed by their accumulation in breast cancer cells, while they revealed no tendency to target normal human osteoblast cells under continuous flow condition in vitro. Biodistribution studies in vivo, not only showed a prolonged circulation for the NKsomes compared to liposomes, but also an increased accumulation in the breast tumor and RES organs was observed. Finally, the antitumor efficiency of the DOX‐containing NKsomes confirmed to be superior compared to the free DOX, regarding the tumor volume after treatment. In another study, Pitchaimani et al. have developed a biomimetic MRI and NIR fluorescence imaging system using NKCs’ membrane.^[^ ^170^ ^]^ To this end, PLGA NPs, a phospholipid‐conjugated gadolinium‐based contrast agent (lipid‐Gd), and membrane fragments from activated NKCs with various ratios were coextruded to form different types of biomimetic nanoconstructs (BNcs) and further attached to a NIR‐dye. The protein composition analyses confirmed the presence of the NKC's key tumor‐targeting receptors, NKG‐2D and NKp30, on the surface of the BNcs. Then, among the various formulations, the most stable BNc with PLGA:lipid‐Gd:membrane protein ratio of 5:1:1, a diameter of 134 ± 4.4 nm and a zeta potential of −41.1 ± 0.59 mV were co‐incubated with the breast cancer cells to evaluate their uptake efficiency. It was observed that BNcs accumulated in the perinuclear intracellular space and their accumulation in the tumor cells was higher than that of bare PLGA NPs in vitro. Besides, studies revealed that only about 2% and 10% of the Gd content would leak from the BNcs within 48 h in the physiological and acidic pH, respectively. In the next step, prolonged circulation, as well as accumulation in the tumor was double that in the bare PLGA NPs, which were significantly declared with the BNcs. Additionally, they had a deep penetration in the tumor tissue, providing improved MRI results.

**Figure 22:**
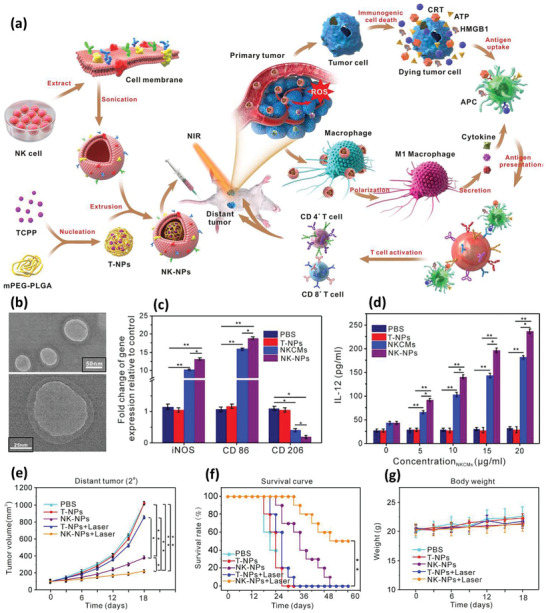
a) Schematic demonstration of NK cell‐membrane‐cloaked NPs for combined PDT and immunotherapy. NK cell‐membranes could elicit proinflammatory M1 M*ϕ*s polarization in tumor for triggering antitumor immunotherapy. Infiltration of effector TCs (CD4^+^ T cells and CD8^+^ T cells) in tumors could efficiently inhibit both primary and abscopal tumors. b) TEM image of the NK‐NPs. c) Gene expression associated with the M1 phenotype in vitro following various treatments. d) In vitro level of proinflammatory IL‐12 cytokine in THP‐1 cells treated with human NK‐NPs, human NK cell membranes (NKCMs), and T‐NPs. e) The antitumor response following various treatments in vivo in abscopal tumors in terms of the tumor volume. f,g) Animal survival and body weight of animals in different groups during the in vivo study. Reproduced with permission.^[^ ^172^ ^]^ Copyright 2018, American Chemical Society.

In addition to improving the targeting efficiency and tumor homing ability of the NPs or drugs, cloaking in the NKCs’ membrane has been reported to induce M1 M*ϕ* polarization, which is favorable for eradicating cancer cells. As it is schemed in **Figure** **22**a, to design a potent tumor treatment based on PDT, the photosensitizer 4,4′,4″,4‴‐(porphine‐5,10,15,20‐tetrayl) tetrakis (benzoic acid) (TCPP) was loaded within the poly(ethylene glycol) methyl ether‐*block*‐poly(lactide‐*co*‐glycolide) (mPEG‐PLGA) to form T‐NPs and then coextruded with the membrane vesicles from the NKCs to form NK‐NPs, which had a spherical core–shell structure (Figure 22a), a hydrodynamic diameter of 85 ± 1.2 nm and a zeta potential of −11.8 ± 0.8 mV. As a minimally invasive cancer therapy approach, PDT can kill tumor cells by utilizing TCPP photosensitizer to generate ROS under light irradiation. However, impaired tumor cells can generate DAMPs (such as calreticulin (CRT), high‐mobility group box 1 (HMGB1), and adenosine triphosphate (ATP)) by PDT‐induced immunogenic cell death (Figure 22a). CRT acts as an “eat me” signal, driving the engulfment of dying cancer cells by phagocyte cells and the uptake of tumor antigens by DCs to stimulate the production and proliferation of tumor‐specific effector T cells. The release of both HMGB1 and ATP also triggers the optimal presentation of tumor antigens to T lymphocytes.^[^ ^171^ ^]^ Nevertheless, PDT‐mediated antitumor immune responses are insufficient to elicit all immunological pathways needed for effective cancer therapy. Therefore, to promote a strong, prolonged, and systemic antitumor immune response, this study combined PDT with immune inducers during antitumor therapy. It was revealed that the NK‐NPs (Figure 22b) maintain the protein composition of the parent NKCs on their surface, specifically those with key roles in tumor targeting (DNAX accessory molecule‐1 (DNAM‐1) and NKG‐2D) and M1 M*ϕ* polarization (immunity‐related GTPase family M member 1 (IRGM1), cannabinoid receptor 1 (CB1), galectin‐12, RAB10, and receptor activator of nuclear factor kappa‐Β ligand (RANKL)) to cope with the immunosuppressive environment of the tumor tissue. Further, as a result of membrane coating, improved stability and diminished TCPP leakage compared to the naked T‐NPs were revealed in the NK‐NPs. In vitro cell culture studies and in vivo biodistribution investigations confirmed the tumor recognition and accumulation potential of the NK‐NPs in comparison with the bare T‐NPs. To examine the NK‐NPs ability to induce M1 polarization in M*ϕ*s, the cells were cultured in the presence of the NK‐NPs or T‐NPs. It was observed that the M1 phenotype markers (iNOS and CD86) as well as the M1 related cytokines (TNF‐*α*, IL‐6, and IL‐12) were upregulated, while the M2 marker CD206 and M2 related cytokine (IL‐10) was significantly downregulated in the presence of the NK‐NPs, but not the T‐NPs (Figure 22c,d). In the tumor mass of the mice treated with the NK‐NPs, the quantity of M1 M*ϕ*s was almost 5.5 times more than that of the mice treated with the T‐NPs. After the approval of tumor‐targeting efficiency and M1 phenotype induction of the NK‐NPs, they were studied for their PDT efficiency. Due to the mentioned tumor targeting, in the presence of irradiation, in vivo ROS generation and apoptosis induction in the tumors treated with the NK‐NPs were more than that of those treated with T‐NPs. To explore the antitumor effect of PDT with NK‐NPs on the abscopal tumors, a bilateral subcutaneous mammary carcinoma mouse model was developed and subjected to systemic administration of the NK‐NPs. Next, one of the tumor sites (the primary tumor) was irradiated and significant tumor inhibition was observed not only in the primary tumor, but also in the abscopal tumor (not‐irradiated distant tumor site) (Figure 22e), leading to meaningful increased animal survival in comparison with the subjects receiving T‐NPs (Figure 22f,g). Further investigations maintained that this antiabscopal tumor effect was due to the NK‐NP‐mediated PDT leading to immunogenic tumor cell death, which was associated with the DAMPs expression resulting in the host immune system's activation. It was observed that in these mice, DC maturation and the population of both tumor‐infiltrating helper and cytotoxic TCs, as well as the serum concentration of the proinflammatory cytokines were significantly increased. This study provided strong evidence for the potential benefits of combined immunotherapy and PDT.^[^ ^172^ ^]^

## 5 Extracellular Vesicles of Immune Cells

ECVs are natural nano/microscale messengers, substantial for cell‐cell communication, especially in multicellular organisms. The ECVs are classified based on their origin and function into three main categories: the Exos that originate from the endolysosome, the MVs that are the results of cell membrane budding, and the apoptotic bodies that are apoptotic cells’ components being packaged in the cell membrane vesicles, each revealing distinct physicochemical and biological properties. Transferring a variety of lipids, proteins, and nucleic acids, they have been recognized to take part in both hemostasis and pathogenesis, and therefore, they could potentially serve as either therapeutic targets or therapeutic tools.^[^ ^173^ ^]^ ECVs’ secreted from immune cells, including antigen presenting cells (APCs), polymorphonuclear cells, and lymphocytes, play key roles in stimulation and suppression of immune responses in a variety of conditions.^[^ ^174^ ^]^ They have been reported to be efficient in cancer vaccination and immunotherapy, as well as neurodegenerative and inflammatory diseases. To exploit these ECVs as therapeutic carriers, several issues must be taken into consideration, including their source‐related differences, their reproducible production, and purification regarding the physicochemical properties of the ECVs, their engineering possibilities, and their pharmacokinetic–pharmacodynamic characteristics.^[^ ^175^ ^]^ In an experimental setting, various cell sources cultivated under different conditions produce ECVs with dissimilar yields, usually not translatable to the clinical settings. Therefore, several attempts have been made to introduce a high‐yield, robust, and reproducible technique for ECV production. For instance, Integra CELLine is a culture system that not only allows an increased ECV production, but also confines the ECVs with a semipermeable membrane.^[^ ^176^ ^]^ Several methods are available for isolation of the ECVs from culture media or biological environments, including ultracentrifugation, ultrafiltration, size exclusion chromatography, aqueous two‐phase system, polymer‐based precipitation, and immunological separation.^[^ ^177^ ^]^ ECVs’ surface and contents could be engineered via manipulating their source cell or by being directly modified after production and purification.^[^ ^178^ ^]^ For example, the parent cells could be transfected with nucleic acids or be co‐incubated with small or large therapeutic molecules, while the cargos could be loaded into the ECVs via simply mixing, electroporation, sonication, extrusion, saponin treatment, or repetitive freeze–thaw cycles.^[^ ^179^ ^]^ Although being in their infancy, recently developed microfluidic technologies have facilitated and accelerated the ECVs preparation, form the purification steps to their surface engineering, making scale up fabrication of these ECVs possible even for clinical purposes.^[^ ^180^ ^]^ Engineering of ECVs is done for various purposes including prolonged circulation time, improved targeting, and intracellular drug delivery. ECVs have been demonstrated to be rapidly cleared from circulation via phagocytic cells following intravenous administration. The use of PEGylation and insertion of “self” markers like CD47 has been reported to increase the ECVs circulation time. ECVs usually do not have targeting properties for a specific cell type, hence many studies evaluated various targeting moieties, including peptides,^[^ ^181^ ^]^ aptamers,^[^ ^182^ ^]^ polysaccharides,^[^ ^183^ ^]^ as well as exploiting magnetic NPs^[^ ^184^ ^]^ to develop targeted DDSs. Just like synthetic NPs, an impediment of cytoplasmic drug delivery with ECVs is the target cells’ uptake machinery, which ends in the endolysosomal compartment. Although, it has been reported that ECVs from specific sources (like DCs), and those engineered with specific materials (like viral components and several types of peptides) could avoid the endolysosomal pathway.^[^ ^185^ ^]^ In addition to the targeting properties of the ECVs, it is also noticeable that ECVsʼ administration through various routes, including intravenous, intraperitoneal, oral, intranasal, subcutaneous, and intratumoral, culminate in different biodistribution, treatment efficiency, and off‐target toxicity.^[^ ^186^ ^]^ In the following sections, we will have a look at the most recent advances in exploiting the immune cell‐derived ECVs for ameliorating hard‐to‐treat diseases, with a glance at the various aspects of their preparation, physicochemical properties and their interaction with the biological environment.

### 5.1 Dendritic Cell‐Derived Extracellular Vesicles (D‐ECVs)

D‐ECVs including Exos inherit the antigen‐presenting properties of their parent cells by carrying MHC‐I and II complexes, while not inheriting several disadvantages associated with the living DCs, which would limit their exploitation in immunotherapy and drug delivery. These include expensive and difficult scale‐up procedure, limited stability and shelf life, the hardly controllable phenotype of the cells especially in the tumor microenvironments with immunomodulatory signals, and off‐target toxicity potential in case of unpredicted cell replication in vivo.^[^ ^187^ ^]^

Several studies have harnessed the DC‐derived ECVs for cancer vaccination and immunotherapy. Wahlund et al. have investigated the potential immune response stimulated by the Exos or MVs derived from OVA‐pulsed DCs.^[^ ^188^ ^]^ Despite their similar physical characteristics and even likeliness in the expression of MHCI, MHCII, CD40, CD54, CD80, CD81, CD86, and LFA‐1, they were distinguishable due to the presence of alpha‐actinin 4 and syntenin‐1 in MVs and Exos, respectively. Following a scheduled i.v. administration of the MVs or Exos to mice, it was revealed that only Exos could activate the germinal center B cells, as well as the production of OVA‐specific cytotoxic TCs in the spleen. Moreover, utilizing Exos led to higher OVA‐specific IgG concentration compared to the MVs. These observations were attributed to the Exos’ significantly higher capacity to incorporate the antigen. Another group of researchers has investigated whether utilization of different adjuvants for parent DCs’ maturation during Exo production, affects the antitumor vaccine potential of the resulted Exos or not. In this regard, Exos from untreated DCs (DEXO_unt_), Exos from DCs treated with OVA as antigen (DEXO_OVA_), and Exos from DCs treated with either of the three adjuvants LPS (TLR4 ligand) (DEXO_OVA+LPS_), pIC (TLR3 ligand) (DEXO_OVA+pIC_), and CpGB (TLR9 ligand) (DEXO_OVA+CpGB_) were extracted from DCs’ culture media (X‐VIVO 15) via serial ultracentrifugation. The cup‐form vesicles had a diameter of 30–150 nm and no significant difference in their physical properties was observed. The five developed formulations were injected into mice intravenously or intradermally, 1 day after they had received OVA‐specific naïve cytotoxic TCs (naïve OT1 cells). Next, OT1 maturation and proliferation were studied 6 days after vaccination in the spleen and lymph nodes of the animals and it was revealed that DEXO_OVA+pIC_ had the highest stimulatory effects on naïve OT1 cells. Both i.v. and intradermal routes of administration for the developed vaccines led to the acceptable immunogenicity in mice. Further in vivo studies also demonstrated that vaccination with DEXO_OVA+pIC_, would specifically lead to a T helper 1 (Th1) proinflammatory phenotype in helper and cytotoxic TCs, as well as a cellular rather than humoral immune response. In the next step, the same ECV production protocol was adapted to produce DEXO_B16+pIC_ against B16F10 melanoma and intradermal administration of the vaccine led to a delayed, but not inhibited, tumor progression and increased animal survival. The underlying mechanism could be the significant recruitment of DEXO_B16+pIC_ in the tumor‐draining lymph nodes, leading to significant activation of B16F10‐specific effector CD8+ TCs and homing of effector TCs, NKCs, and NK‐TCs to the tumor site.^[^ ^189^ ^]^

**Figure 23:**
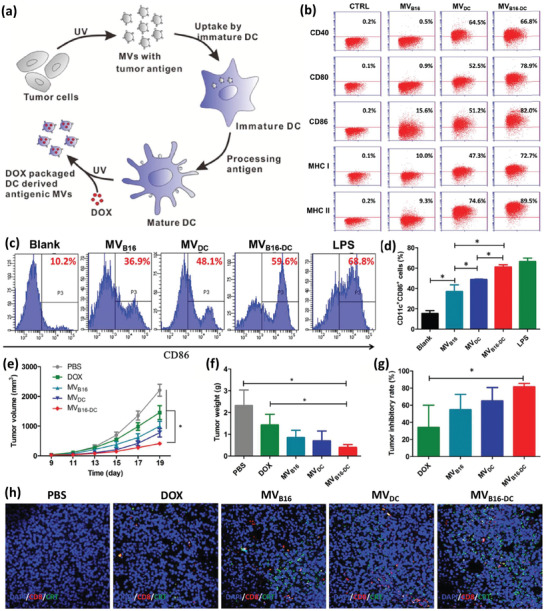
a) Schematic illustration of preparing MV_B16‐DC_. b) Flow cytometric analysis of the phenotypes of MV_B16_, MV_DC_, and MV_B16‐DC_. c) Flow cytometry analysis of CD 86 expression in CD11c^+^ cells after incubating BMDCs at different conditions. d) Quantitative analysis of CD11c^+^CD86^+^ cells by flow cytometry (*n* = 3, *p < 0.05). e) Melanoma tumor volume after administrating various formulations (*n* = 6, *p < 0.05). f) Tumor weight at day 19 after treatment with different formulations (*n* = 6, *p < 0.05). g) Tumor inhibitory rate of melanoma (*n* = 6, *p < 0.05). h) Immunofluorescence staining of tumor tissues. Nucleus of cancer cells, CD8^+^ TCs, and CRT^+^ cells for ICD recognition were stained with blue DAPI, red anti‐CD8 Ab, and green anti‐CRT Ab, respectively. Reproduced with permission.^[^ ^190^ ^]^ Copyright 2017, Wiley‐VCH.

To evaluate the D‐ECVs’ potential for both immunotherapy and chemotherapy, Wu et al. have conducted a study and compared its results with that of the tumor cell‐derived ECVs.^[^ ^190^ ^]^ First, a melanoma cell line was radiated by UV light to induce ECV production (MV_B16_). The resulting particles were then incubated with DOX and showed a dose‐dependent loading efficiency. They also had good stability in PBS and serum for 5 days, a pH‐independent release profile in pH 7.4 and 6.8, and a protein composition similar to the parent cell, especially in terms of tumor antigens and cytoskeleton proteins. In the second step, DCs were incubated with MV_B16_ as the tumor antigen, and with LPS for upregulation of co‐stimulatory molecules and transporting the formed MHC–peptide complexes to the cell surface. Then the DC‐derived ECVs (MV_B16‐DC_) were produced with the same protocol as MV_B16_ (**Figure** **23**a). They were also packed with DOX and a similar drug loading and release pattern as the MV_B16_ were observed for them. In comparison with MV_DC_ prepared from DCs that were not incubated with the MV_B16_, MV_B16‐DC_ had significantly more MHCI, MHCII, CD80, and CD86 (Figure 23b) expression, leading to higher induction of TC activation. In vitro uptake studies revealed that melanoma cells take up both MV_B16_ and MV_B16‐DC_ with similar efficiencies, leading to their equal antitumor activity, while free DOX had less tumor cell uptake and hence lower antitumor effectiveness. It was demonstrated that MV_B16‐DC_ could effectively stimulate maturation of the naïve DCs, as shown by its significant effect on increased expression of CD86 in CD11c^+^ cells after incubating with bone marrow dendritic cells (BMDCs) (Figure 23c,d). Further in vivo studies revealed significant tumor accumulation and retention of all of the ECV formulations. Moreover, following the same drug administration schedule, the most significant tumor suppression was observed in melanoma mice models receiving MV_B16‐DC_ (Figure 23e–g). Histological analysis of tumor‐draining lymph nodes and resected tumors further revealed that the underlying mechanism of such prominent antitumor efficiency could be the significant induction of DCs, helper TCs, and cytotoxic TCs’ activation. This may not only be a result of antigen presenting by the MV_B16‐DC_, but also via DAMP expression and immunogenic cell death (ICD) effect of the DOX through the recruitment of APCs for tumor antigen processing and presentation.^[^ ^191^ ^]^ CRT is one of the DAMPs that can be used to identify ICD. After different treatments, the tumor tissues were therefore stained with anti‐CD8 Ab and anti‐CRT Ab. The confocal microscopy images showed that MVs induces stronger expression of CRT than free DOX as a result of better uptake and accumulation in the tumor tissue, while there was no significant difference among the three tested MVs (Figure 23h). All in all, because of the protective vaccine‐like effect of the developed formulations, it was concluded that the D‐ECVs had a synergistic immuno–chemotherapeutic potential for cancer eradication.

Besides being compared to their parent cells, D‐ECVs have been compared to synthetic material like liposomes for their antigen presenting and drug delivery capabilities. In one case, D‐ECVs purified with density gradient ultracentrifugation from JAWSII cell culture, and fusogenic liposomes based on 1,2‐dioleoyl‐*sn*‐glycero‐3‐phosphoethanolamine (DOPE) and 3*β*‐hydroxy‐5‐cholestene 3‐hemisuccinate (CHEMS) were compared for cytoplasmic delivery of small RNAs. The D‐ECVs had a particle size of 30–300 nm, a negative surface charge, and revealed the capability of incorporating cholesterol‐conjugated siRNA (chol‐siRNA) within their membranes. Although, despite the fusogenic liposomes having a similar size and zeta potential, the D‐ECVs could not deliver their associated siRNA to the target cells, and therefore not efficiently causing the expected gene silencing response. This phenomenon was demonstrated to be based on the D‐ECVs inability to avoid the endolysosomal pathway when being taken up by the target cells.^[^ ^192^ ^]^

To improve D‐ECVs’ targeting properties, aptamers have been widely investigated as a targeting moiety. A study has reported incorporating a nucleolin‐binding aptamer, AS1411, in D‐ECVs for targeted delivery of RNA molecules to breast cancer lesions.^[^ ^193^ ^]^ To this end, first MCs were derived from mice bone marrow and differentiated into naïve DCs. D‐ECVs were then purified by serial ultracentrifugation. Second, cholesterol‐modified peptide, 1‐(3‐dimethylaminopropyl)‐3‐ethylcarbodiimide hydrochloride (EDC), NHS, and NH_2_‐AS1411 were mixed to form cholesterol‐conjugated aptamers (T‐AS1411). Then, the D‐ECVs and T‐AS1411 aptamers were incubated and allowed the aptamers to be incorporated in the vesicles’ membrane via their cholesterol group. Next, the miRNA cel‐miR‐67 was loaded into the aptamer‐conjugated vesicles via electroporation and in the defined condition, the loading efficiency was determined to be about 3%. The drug‐loaded vesicles were spherical, had a mean diameter and a zeta potential of about 77 nm and −16.4 mV, respectively. The vesicles released ≈98% and 94% of their miRNA content within 24 h in serum and PBS, respectively. Further in vitro and in vivo studies confirmed that the developed platform had a remarkable potential for targeted intracellular delivery of miRNA, leading to a significant tumor inhibitory effect.

Another study recently reported the potential of targeted drug delivery with aptamer‐conjugated Exos and its plausible underlying mechanisms. First of all, Exos were purified from the immature DCs culture media through differential ultracentrifugation. The Exos were cup‐shape with an average diameter of 55.0 ± 12.4 nm (hydrodynamic diameter of ≈91.2 nm). Then, sgc8 nucleic acid aptamer (recognizing PTK7 on the tumor cell membrane) was attached to a diacyl lipid via a PEG linker, spontaneously being inserted in the lipid bilayer of the Exos during an incubation period. As a result of being decorated with aptamers, the hydrodynamic size of the Exos slightly raised to 111.4 nm, and their zeta potential was determined to be −2.06 mV. In vitro studies demonstrated that aptamer‐conjugated Exos could specifically recognize PTK positive tumor cells and localized in their lysosome after being taken up. Utilizing a variety of internalization pathways’ inhibitors, it was observed that the major mechanism for the energy‐dependent uptake of aptamer‐conjugated Exos was clathrin‐mediated endocytosis, while caveola‐mediated endocytosis and micropinocytosis also played minor roles. Finally, being loaded with DOX via electroporation (14.157 ng DOX per 1 µg of Exos), it was confirmed that the aptamer‐conjugated Exos selectively deliver higher concentrations of DOX to PTK‐positive tumor cells compared to the nontarget cells.^[^ ^182^ ^]^

### 5.2 Macrophage‐Derived Extracellular Vesicles

ECVs derived from M*ϕ*s play important roles in a variety of physiological and pathological conditions because of their ability to transport small and large molecules.^[^ ^194^ ^]^ Therefore, these ECVs seem to hold great potential for drug delivery purposes and various investigations from in vitro settings to clinical trials have been conducted to evaluate their efficiency.^[^ ^173^, ^195^ ^]^ Before these evaluations become useful, it is necessary to characterize these ECVs comprehensively.^[^ ^174^, ^178^ ^]^ A study conducted by Charoenviriyakul et al. has investigated several properties of Exos of various origins including murine M*ϕ*s.^[^ ^196^ ^]^ Among the tested cell lines, murine M*ϕ*s had a relatively high yield of Exo production (6 × 10^8^ Exos µg^−1^ protein), with particles in the same size range as other cells (105 ± 4 nm) and a zeta potential of −38.8 ± 0.6 mV. In **Table** **2**, an attempt to offer a concise review of the most recent studies focusing on DDSs based on natural M*ϕ*‐derived ECVs has been provided, while more detailed description of novelties is offered in the following paragraphs.

**Table 2:** Examples of M*ϕ*‐derived vesicles for therapeutic applications

Particle^a)^	Fabrication method	Size [nm] Zeta potential [mV]	Main findings	Refs.
DOX‐loaded AuNR‐FA‐RGD‐modified Exos	1) The Exos were prepared by culturing M*ϕ* cells in medium containing DSPE‐PEG‐RGD and DSPE‐PEG‐SH to modify the membrane of the cells with RGD and sulfhydryl groups. 2) DOX was loaded using the electroporation method before AuNRs attachment on the Exo through the formation of Au—S bonds. 3) Exos were further functionalized by FA through covalent bonds to obtain DOX‐loaded AuNR‐FA‐RGD‐modified Exos.	≈225 nm ≈−20 mV	The cooperative dual ligand targeting property of the engineered Exos endowed them with high accumulation at the tumor site through receptor‐mediated endocytosis. The heat generated by AuNRs could enhance the permeability of the Exo membrane to increase DOX release from the carriers, thus inhibiting tumor recurrence in a programmable manner. Superb chemo–photothermal synergistic therapeutic effect was observed by the DOX‐loaded AuNR‐FA‐RGD‐modified Exos both in vitro and in vivo.	^[^ ^197^ ^]^
Serum‐derived Exos	1) Serum‐derived Exos were prepared by ultracentrifugation or polymer precipitation methods. 2) Electroporation or incubation method was employed to encapsulate immune‐stimulating biomolecules within Exos.	36 nm −22 mV	Among mouse serum, human serum, and FBS‐derived Exos, the latter one exhibited narrower size distribution and higher yield of production for lymph node delivery of immune‐stimulating biomolecules. The loading efficiencies of CpG ODN, OVA, and MPLA into FBS‐derived Exos were 0.4 ± 0.1, 41.5 ± 5.3, and 41.0 ± 9.6%, respectively. FBS‐derived Exos were delivered not only to subcapsular sinus M*ϕ* zones but also to T cell zones of lymph node, allowing efficient stimulation of both T cells and APCs. The encapsulation of the immune‐stimulating molecules within FBS‐derived Exos greatly increased intracellular delivery to M*ϕ*s via phagocytic pathways, which induced higher TNF‐*α* and IL‐6 secretion than free biomolecules. MPLA‐incorporated Exos induced higher TNF‐*α* and IL‐6 levels in vitro and, importantly, they activated and differentiated T cells, which elevated cytokine IFN‐*γ* and TNF‐*α* induction for CD3^+^ T cells in vivo, confirming that the FBS‐derived Exos were efficient biocarriers to deliver immune stimulators to lymph nodes to elicit desired immune responses.	^[^ ^198^ ^]^
TPP1‐l or TPP1‐t‐loaded Exos	1) TPP1‐t‐loaded Exos were prepared by the incorporation of TPP1 into M*ϕ*‐derived Exos through transfection of parental cells with TPP1‐encoding pDNA. 2) TPP1‐l‐loaded Exos were made by the loading of TPP1 into empty Exos. Saponin or sonication in the presence of TPP1 was used to permeabilize Exos membranes.	≈130 nm NDet	The Exos significantly increased the stability of TPP1 against protease degradation and provide efficient TPP1 delivery to target cells in an in vitro model of neuronal ceroid lipofuscinoses 2, a lysosomal storage disorder. Using confocal studies, it was confirmed that the majority of the TPP1‐loaded Exos were delivered to lysosomes. It was found that TPP1‐l‐loaded Exos delivered more enzyme to CLN2 cells than the TPP1‐t‐loaded Exos. Remarkable brain accumulation of Exo carriers and increased lifespan was recorded in the LINCL mouse model after the intraperitoneal administration of TPP1‐loaded Exos.	^[^ ^194b^ ^]^
MiRNA‐155‐loaded‐Exos	1) Exo purification was carried out by the ultracentrifugation method. 2) Electroporation method was used to transfer MiRNA‐155 into Exos derived from M*ϕ*s to prepare MiRNA‐155‐loaded‐Exos.	NDet	MiRNA‐155 promoted the expression of inflammatory cytokines, including TNF‐*α*, IL‐23, and IL‐6, and boosted the expression of MCH‐I, CD40, CD63, and CD81. Inflammatory signal proteins, such as NF‐*κ*B and MyD88 in *H. pylori* infection. M*ϕ*s were downregulated because of the overexpression of MiRNA‐155. It was shown that supernatant of cell culture of M*ϕ*s transfected with MiRNA‐155‐loaded‐Exos could prevent the proliferation of *H. pylori*, thus confirming that cytokines secreted from M*ϕ*s had a crucial role in the inhibition effect. MiRNA‐155 triggered M*ϕ*s to kill or inhibit *H. pylori* by regulating the inflammatory response of cells to hamper gastritis caused by *H. pylori* infection.	^[^ ^199^ ^]^
PTX‐loaded‐Exos	1) Exos were harvested from the supernatants of RAW 264.7 cells cultured in Exo‐depleted media utilizing the ExoQuick‐TC Kit. 2) Next, incubation at room temperature, sonication, and electroporation methods were employed to incorporate PTX into the Exos to prepare PTX‐loaded‐Exos.	132–288 nm	Using different methods including sonication, electroporation, and incubation at room temperature, the amount of PTX loaded into Exos was determined to be 28.29 ± 1.38, 5.3 ± 0.48, and 1.44 ± 0.38, respectively. Particle diameters were also 287.7 ± 0.7, 145.3 ± 1.0, and 132.2 ± 2.3, respectively. It was found that incorporation of DOX, a Pgp substrate, into the Exos significantly increased DOX accumulation in MDR cells as compared to free DOX, confirming overcoming of Pgp‐mediated drug efflux by the drug‐loaded Exos. PTX‐loaded‐Exos increased cytotoxicity more than 50 times in drug‐resistant MDCK_MDR1_ (Pgp^+^) cells. Using the PTX‐loaded‐Exos, nearly complete colocalization of the Exos with cancer cells in a model of murine Lewis lung carcinoma pulmonary metastases as well as potent anticancer effects were observed.	^[^ ^200^ ^]^
PTX‐loaded‐AA‐PEG‐Exos	1) AA‐PEG‐Exos were prepared by conditioned media of the supernatants of RAW 264.7 cells cultured in Exo‐depleted media utilizing the ExoQuick‐TC Kit. 2) DSPE‐PEG‐AA was added to the mixture of the isolated Exos, and PTX was then added to the mixture to incorporate PTX into the Exos using sonication method.	305 nm −4 mV	PTX loading capacity for the AA‐PEG‐Exos formulation was ≈33%. Robust accumulation of systemically administered AA‐PEG‐Exos with cancer metastases was observed, highlighting the importance of both AA‐vector, and LFA‐1 protein expressed on exosomal membranes. PTX‐loaded‐AA‐PEG‐Exos showed a potent antitumor effect against LLC mouse model of pulmonary metastases.	^[^ ^201^ ^]^
Iron oxide NP loaded‐EXOs	1) THP1‐M*ϕ*s, previously treated with phorbol 12‐myristate 13‐acetate were incubated with drugs and magnetic NPs to obtain iron oxide NPs and different drug‐loaded M*ϕ*s. 2) To harvest drug/photosensitizer‐iron oxide NP loaded‐Exos, THP1‐M*ϕ*s were cultured in serum‐free medium for 2 days. Cell stress induced by serum depletion released Exos. Magnetic sorting was used to purify the MVs from the conditioned starvation medium.	673 nm −30 mV	Human M*ϕ*s were successfully loaded with iron oxide NPs and different cargos, including DOX and t‐PA, as drugs, as well as TPCS2a and mTHPC, as photosensitizer to produce M*ϕ*‐derived MVs. The hybrid cell MVs were magnetically guided and readily manipulated by magnetic forces. It was demonstrated that the magnetic field induced faster drug uptake. Utilizing photosensitizer‐loaded vesicles, uptake of the MVs by cancer cells could be kinetically modulated and spatially controlled under the magnetic field. It was shown that cancer cell death was improved by magnetic targeting.	^[^ ^202^ ^]^
M1 Exos	1) M1 polarization of M*ϕ*s was carried out in the presence of IFN‐*γ* as inducer. 2) M1 Exos were isolated by differential centrifugation method.	50 nm −45 mV	It was demonstrated that the M1 Exos could be home to the lymph node after subcutaneous injection and taken up by the DCs and M*ϕ*s, thus boosting the release of a pool of Th1 cytokines. When M1 Exos were used as adjuvant with lipid calcium phosphate NP‐encapsulated Trp2 vaccine, significant growth inhibition of melanoma was observed than CpG oligonucleotide as adjuvant alone.	^[^ ^203^ ^]^
miR‐143BP‐loaded‐Exos	1) Transfection of miR‐143BPs with THP‐1 cells was achieved by using CL, according to the manufacturer's Lipofection protocol. 2) miR‐143BPs‐Exos were harvested by incubation of the miR‐143BP‐transfected THP‐1 cells in fetal bovine serum‐free medium followed by ultracentrifugation.	NDet	After i.v. injection of miR‐143BP‐transfected THP‐1 cells, miR‐143 was detected in the serum 8 h after the last injection and mainly in the kidneys and tumor, confirming that the cells had generated miR‐143BP‐loaded‐Exos.	^[^ ^204^ ^]^
LZD‐loaded‐Exos	1) After culturing of RAW 264.7 cells with Exo depleted serum, the M*ϕ*‐derived Exos were harvested from the culture supernatants utilizing the ExoQuick‐TC Kit. 2) LZD was loaded into Exos by the co‐incubation method at 37 °C.	76 nm −7 mV	The loading capacity of the EXOs for LZD was 5.06% ± 0.45%. The LZD‐loaded‐Exos was more effective against intracellular MRSA infections in vitro and in vivo than the free LZD. The Exos efficiently accumulated in M*ϕ*s and a potent intracellular bactericidal effect was observed by the Exos loaded with LZD.	^[^ ^205^ ^]^
DOX‐loaded‐Exos	1) DOX‐loaded‐Exos were prepared by incubating PCCs, PSCs, or M*ϕ*s with DOX. 2) Differential ultracentrifugation was employed to isolate the drug‐loaded Exos.	120–155 nm NDet	Among the different cell types tested in this study, PCCs generated the most Exos and exhibited most efficient in drug loading (14.06 ng µg^−1^) followed by M*ϕ*s (7.27 ng µg^−1^) and PSCs (3.99 ng µg^−1^). M*ϕ*‐derived Exos loaded with DOX showed the highest antitumor activity than the two above mentioned cell‐derived Exos.	^[^ ^206^ ^]^
miR‐365‐loaded‐Exos	Exos were prepared by differential centrifugations using M2‐polarized murine peritoneal M*ϕ*s.	≈90 nm NDet	Through a comprehensive mechanistic study, it was shown that TAM communicated with the TME via secretion of ≈90 nm Exos, which was selectively uptaken by cancer cells. In both in vitro and in vivo tests, it was demonstrated that the secreted M*ϕ*‐derived exosomes significantly reduced the sensitivity of pancreatic ductal adenocarcinoma cells to gemcitabine. Such effect was mediated by the transfer of miR‐365 in M*ϕ*‐derived exosomes, impairing activation of gemcitabine. It was confirmed that the immune transfer of the miR‐365 antagonist boosted the sensitivity to gemcitabine. In contrast, adoptive transfer of miR‐365 in TAM induced gemcitabine resistance in pancreatic ductal adenocarcinoma‐bearing mice. This showed the importance of blocking of miR‐365 to increase gemcitabine response.	^[^ ^207^ ^]^
MicroRNA‐223‐loaded‐Exos	Exos were harvested by differential centrifugations using IL‐4‐activated M*ϕ*s.	57 nm NDet	Using a coculture system containing breast cancer cells and IL‐4‐activated M*ϕ*s and without direct cell‐cell contact, the shuttling of exogenous MicroRNAs from IL‐4‐activated M*ϕ*s to cocultivated breast cancer cells was observed. MicroRNA‐223 was detected within the Exos released by the IL‐4‐activated M*ϕ*s and elevated in the cocultivated SKBR3 and MDA‐MB‐231 cells significantly. Breast cancer cell invasion was decreased when the M*ϕ*s were treated with a MicroRNA‐223 antisense oligonucleotide that inhibited the expression of MicroRNA‐223, demonstrating MicroRNA‐223 was responsible for M*ϕ*‐promoting breast cancer cell invasion.	^[^ ^208^ ^]^
*M. tuberculosis* CFP pulsed Exos	RAW 264.7 mouse M*ϕ*s were treated with CFP and then differential centrifugations were used to harvest Exos from M*ϕ*s pulsed with *M. tuberculosis* CFPs.	50–150 nm NDet	It was demonstrated that Exo‐derived from M*ϕ*s pulsed with *M. tuberculosis* CFPs induced antigen specific IFN‐*γ* and IL‐2‐expressing CD4^+^ and CD8^+^ T cells. In the *M. tuberculosis* mouse model, it was found that the Exo derived from M*ϕ*s pulsed with *M. tuberculosis* CFPs as cell‐free vaccine, primed protective immune response, and boosted prior BCG immunization as well. Similar Th1 immune responses but more limited Th2 responses were found in the Exo‐vaccinated mice than the BCG‐vaccinated mice.	^[^ ^209^ ^]^
IFN‐*α*‐Exos	IFN‐*α*‐treated M*ϕ*s or LSECs were prepared based on differential ultracentrifugation method.	<100 nm NDet	After cultivation of IFN‐*α*‐treated M*ϕ*s or SECs together with replicable HBV, it was found that the replication of HBV was greatly restricted, thus, confirming that uninfected cells adjacent to infected cells efficiently conferred the antiviral activity of IFN‐*α* on the infected cells through Exos from the IFN‐*α*‐treated liver nonparenchymal cells. It was found that the Exos involved in the antiviral response of IFN‐*α* to the mouse hepatitis virus A59 and the adenovirus in mice as well.	^[^ ^210^ ^]^
Exos derived from BMDMs	Exos derived from BMDMs were prepared by the ultracentrifugation method after lipopolysaccharide stimulation of BMDMs.	105 nm NDet	It was found that, after nerve injury, ROS production in injured nerve recruited CX3CR1‐dependent inflammatory responses. The study demonstrated that functional NADPH oxidase 2 complexes incorporated within M*ϕ* Exos released the enzyme into injured axons via endocytosis of the Exos. The internalized active NOX2 was retrogradely transported to the cell body mediated by importin‐*β*1–dynein‐dependent mechanism. Subsequently, the released NOX2 oxidized PTEN through cysteine oxidation of the protein, thus downregulating its activity and then stimulating PI3K signaling to achieve axonal regeneration.	^[^ ^211^ ^]^
Immunoadjuvant‐loaded‐Exos	After J774 cell line, WEHI‐164 cell lysate, HSP70 enriched WEHI‐164 cell lysate, PRP, and NLX as adjuvant were added into separated flasks containing fresh medium without FBS, and then corresponding Exos were purified by centrifugation and subsequent filtration.	40–150 nm NDet	Among different tested immunoadjuvants, it was found that HSP70‐enriched Exos were more effective than others, stimulating more immune responses in animal models and, therefore, leading to more efficient tumor regression. Compared to the control group, NLX‐enriched Exos exhibited less splenocyte proliferative responses in treated mice and the tumor number was constant after 28 days.	^[^ ^212^ ^]^

^a)^ Abbreviations: AA‐PEG: aminoethylanisamide‐polyethylene glycol; APCs: antigen presenting cells; AuNR: gold nanorods; BCG: Bacillus Calmette‐Guérin; BMDMs: bone marrow‐derived M*ϕ*s; CD: cluster of differentiation; CFPs: culture filtrate proteins; CL: cationic liposome; CLN2: ceroid lipofuscinoses 2; CpG ODN: CpG oligodeoxynucleotides; CX3CR1: CX3C chemokine receptor 1; DSPE‐PEG: 1,2‐dioleoyl‐*sn*‐glycero‐3‐phosphoethanolamine‐poly(ethylene glycol)‐2000; DSPE‐PEG‐SH: sulfhydryl functionalized 1,2‐dioleoyl‐*sn*‐glycero‐3‐phosphoethanolamine‐poly(ethylene glycol)‐2000; Exos: exosomes; FA: folic acid; FBS: fetal bovine serum; *H. pylori*: *Helicobacter pylori*; HBV: hepatitis B virus; HSP70: heat shock protein 70; IFN‐*α*: interferon‐*α*; IFN‐*γ*: interferon‐*γ*; IL: interleukin; LFA‐1: lymphocyte function associated antigen‐1; LINCL: late‐infantile neuronal ceroid lipofuscinosis; LSECs: liver sinusoidal endothelial cells; LZD: linezolid; M*ϕ*: macrophage; *M. tuberculosis*: *Mycobacterium tuberculosis*; miR‐143BPs: chemically modifed microRNA‐143s; MiRNA‐155: microRNA‐155; MPLA: monophosphoryl lipid A; MRSA: methicillin‐resistant *Staphylococcus aureus*; mTHPC: 5,10,15,20‐tetra(*m*‐hydroxyphenyl)‐chlorin; NADPH; nicotinamide adenine dinucleotide phosphate; NDet: not determined; NLX: naloxone; NOX2: NADPH oxidase 2; OVA: ovalbumin; PCCs: pancreatic cancer cells; PCR: polymerase chain reaction; PI3K: phosphoinositide 3‐kinase; PRP: propranolol; PSCs: pancreatic stellate cells; PTEN: phosphatase and tensin homolog; RGD: arginyl–glycyl–aspartic acid; TAM: tumor‐associated M*ϕ*s; Th: T helper; TME: tumor microenvironment; TNF: tumor necrosis factor; t‐PA: tissueplasminogen activator; TPCS2a: disulfonated tetraphenyl chlorin; TPP1: tripeptidyl peptidase‐1; TRP2: tyrosinase‐related protein 2; TSG101: tumor susceptibility gene 101.

**Figure 24:**
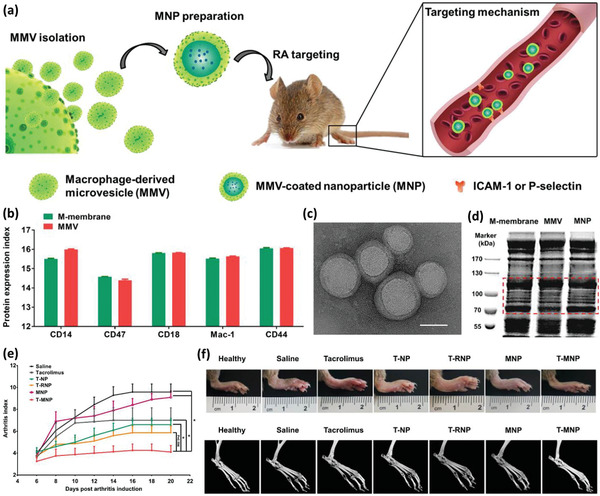
a) Schematic demonstration of the concept behind the study of tacrolimus‐loaded PLGA NPs cloaked with M*ϕ*‐derived ECVs to target rheumatoid arthritis. b) Comparing the surface protein expression of MMV and its mother M*ϕ* membrane (m‐membrane). c) TEM image of the MNPs. d) Protein analysis of m‐membrane, MMV, and MNP. e) Arthritis index in different tested groups over 14 days of treatment; *n* = 5, *p < 0.05. f) Macroscopic swelling and micro‐CT images of the arthritic paw in different groups. Reproduced with permission.^[^ ^214^ ^]^ Copyright 2018, American Chemical Society.

The ECVs characteristics directly dictate the efficiency of their associated DDS and these characteristics may vary based on the different production techniques. For example, M*ϕ*‐derived Exos have been reported to be less effective than serum‐derived Exos in delivering immunostimulating molecules to the lymph nodes because of their larger size. This study compared two Exo isolation methods and it was revealed that the polymer (PEG) precipitation method resulted in Exos with significantly smaller diameters compared to the ultracentrifugation method.^[^ ^198^ ^]^ A significant drawback reported concerning the M*ϕ*s as a source for ECVs is their low yield of production because both research and clinical applications require larger quantities of the vesicles. In this regard, Shyong et al. suggested that adding calcium phosphate particles to the M*ϕ*s and MCs’ culture media would increase their yield of Exo secretion by up to twofold.^[^ ^213^ ^]^ Moreover, another prominent study reported a targeted DDS for rheumatoid arthritis (**Figure** **24**a) by taking the advantage of cytochalasin B to reverse the interaction between the M*ϕ*’s membrane and its cytoskeleton, thus stimulating MVs production with a high yield (0.3 mg ECV per 10^7^ cells).^[^ ^214^ ^]^ The ECVs were then collected via centrifugation from the cell culture and revealed to have similar protein composition to that of the M*ϕ* membrane (Figure 24b). Specifically, they contained self‐marker surface proteins and inflamed endothelium adhesion molecules. The size of macrophage‐derived microvesicle (MMV)‐coated NPs (MNP) (Figure 24c) was about 130 nm with a zeta potential similar to that of the cell membrane, ≈−25.5 mV, while it was observed that the protein composition of the MMVs was the same as MNPs (Figure 24d). Further studies revealed that MNPs are stable regarding size and less coagulation was observed in serum‐containing media than the bare PLGA NPs. MNPs were found to liberate their content in a retarded manner in comparison with the PLGA NPs. In vitro tests clarified significantly higher and specific binding of the MNPs to a cellular model of inflamed endothelia via interactions between CD44‐P selectin and Mac‐1‐ICAM‐1, mimicking the M*ϕ*‐inflamed endothelial cell interactions. In vivo experiments involved mice, where each mouse had one paw with collagen‐induced arthritis and another normal paw. After receiving the developed formulations intravenously, MNPs revealed the highest accumulation in the morbid paw, with no off‐target presence in the normal one, that could be attributed to the significant expression of P selectin and ICAM‐1 in the inflamed joints. Biodistribution studies not only demonstrated a prolonged circulation of the MNPs compared to the naked NPs, but also revealed their prolonged retention in the inflamed tissue compared to the control groups. This led to a significant therapeutic effect of tacrolimus‐loaded MNPs, confirmed by diminished paw swelling as an index of arthritis, mitigated bone erosion, and abated proinflammatory cytokine expression. As shown in Figure 24e, swelling was observed in saline and MNP‐treated groups, while free tacrolimus, tacrolimus‐loaded NP (T‐NP), and tacrolimus‐loaded red blood cell membrane‐coated NP (T‐RNP) could slightly suppress the progression of rheumatoid arthritis, but not completely stop the paw inflammation. In contrast, the tacrolimus‐loaded MNP (T‐MNP) significantly suppressed the severity of rheumatoid arthritis and stabilized the arthritis index. Figure 24f also confirmed that very little paw swelling of mice in the T‐MNP group and the normal and morbid paws appeared similar. Bone erosion investigation by micro‐computed tomography (CT) analysis also revealed that T‐MNP efficiently mitigates bone erosion as a result of drug accumulation in the damaged area.

**Figure 25:**
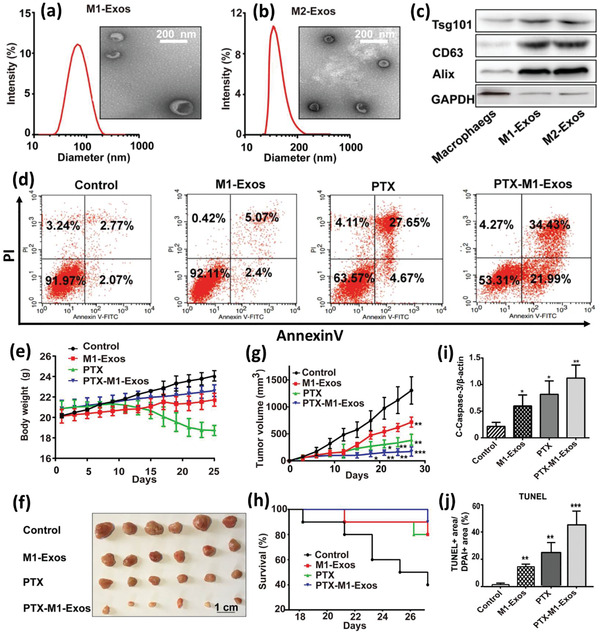
a,b) Size distribution and morphological characterization of M1‐Exos and M2‐Exos. c) Markers of Exos derived from M1 and M2 M*ϕ*s. d) Evaluation of cell apoptosis by flow cytometry at 24 h in different groups. e–h) Body weights, photographic images of tumor size, tumor volumes, and survival rates in each group. PTX‐loaded Exos derived from M1 M*ϕ*s have superior antitumor efficiency in breast cancer mouse models. i) The expression level of caspase‐3/*β*‐actin. j) The quantitative analysis of the apoptosis in the tumor tissues. Values were expressed as mean ± SD (*n* = 6; *p < 0.05, ^**^ *p* < 0.01, and ^***^ *p* < 0.001 vs the control group). Reproduced with permission.^[^ ^215^ ^]^ Copyright 2019, Ivyspring International Publisher.

Exploiting M*ϕ*‐derived ECVs has been a promising strategy for drug delivery in various cancers. A recent study has compared several characteristics of Exos from a pancreatic cancer cell line (PCC), pancreatic stellate cells (PSCs), and M*ϕ*s for delivering DOX to PCCs in vitro. It was clarified that although both Exo shedding quantity and drug loading efficiency were highest for the PCC‐derived Exos, the most significant antitumor efficiency belonged to the Exos originating from M*ϕ*s. This confirmed the superior efficiency of M*ϕ*‐derived Exos for cancer therapy.^[^ ^206^ ^]^ Another research group has reported immunizing animals with Exos from M*ϕ*s that had been treated with heat‐shock protein 70 (HSP70)‐rich tumor cell lysate, result in significantly fewer tumor node formation following inoculation of fibrosarcoma cells and it was deduced that these Exos could be used as an immune adjuvant in cancer immunotherapy.^[^ ^212^ ^]^ Wang et al. have recently investigated the antitumor effect of PTX‐loaded Exos from M*ϕ*s of different phenotypes (M1‐Exo and M2‐Exo).^[^ ^215^ ^]^ In this study, naïve M*ϕ*s were treated with IFN‐*γ* or IL‐4 to present the M1 or M2 phenotypes, respectively. After 24 h, the Exos were extracted from their culture media via ultracentrifugation. Further analyses gave an illustration of the Exos’ shape and size distribution (**Figure** **25**a,b), besides confirming the expression of M1 and M2 Exo markers on the developed vesicles (Figure 25c). In vitro uptake assay demonstrated that breast cancer cells take up M1‐Exos in a time‐dependent manner. Besides, when naïve M*ϕ*s were cultured with the M1‐Exos, their NF‐*κ*B pathway was activated. Apoptosis assay demonstrated that PTX‐loaded M1‐Exos (PTX‐M1‐Exos) induced higher killing effect on the cancer cells than those of the free drug and unloaded M1‐Exos (Figure 25d). In vivo studies on mice with breast cancer further demonstrated that PTX‐M1‐Exos had no negative effect on the body weight of animals, while retarding the tumor growth slightly more than free PTX and enhancing the survival rate of animals (Figure 25e–h). Further studies confirmed higher induction of caspase3 and more positive expression of TUNEL in the tumor cells in PTX‐M1‐Exos‐treated group, confirming that the PTX loading into M1‐Exos could suppress tumor growth (Figure 25i,j). Histological studies also revealed that M1‐Exos penetrate the tumor and activate the residing M*ϕ*s.

Kim et al. have reported that from a technical point of view, drug loading with sonication is far more efficient compared to electroporation and incubation.^[^ ^200^ ^]^ They showed that PTX‐loaded Exos derived from M*ϕ*s, loaded with the sonication technique, produced a significantly higher antineoplastic effect on lung cancer cells with multidrug resistance compared to the free drug and Taxol in vitro. Intranasal administration of the developed formulation led to a significant accumulation of Exos in the lung metastatic nodules in vivo. To improve the targeting efficiency of the developed formulation, a lipid‐conjugated aminoethylanisamide‐polyethylene glycol (AA‐PEG), which specifically targets sigma receptors (overexpressed in several types of lung cancer cells) was incubated with the Exos before being loaded with PTX. Following intravenous injection, the targeted Exos led to significantly more accumulation in the lung metastatic nodes and revealed superior antineoplastic efficiency compared to the nontargeted formulation.^[^ ^201^ ^]^

In addition to the cancers, M*ϕ*‐derived ECVs have extensively been investigated for CNS drug delivery. Haney et al. have evaluated M*ϕ*‐derived Exos for protecting the catalase molecules and delivering them to the brain for the treatment of Parkinson's disease (PD).^[^ ^216^ ^]^ After the Exos were extracted from the culture media with centrifugation, their drug loading efficiency with various methods, including incubation at room temperature with or without saponin, repeated freeze–thaw cycles, sonication, and extrusion was evaluated. The results implied that loading efficiency increased as follows: incubation in the room temperature without saponin < freeze–thaw < incubation in the room temperature with saponin < extrusion ≈ sonication, with the two latter causing a reformation/deformation of Exos. All of the Exos had a diameter of 100–200 nm, with increasing order as follows: incubation in the room temperature without saponin ≈ incubation at room temperature with saponin < freeze–thaw ≈ extrusion < sonication. Atomic force microscopy (AFM) images revealed round particles as a result of incubation or extrusion, while those prepared with freeze–thaw seemed to reveal aggregated vesicles, and sonication resulted in nonspherical particles with various shapes. Comparing the uptake efficiency of the developed formulations in pheochromocytoma12 (PC12) cells showed that, despite significantly larger size, the Exos being loaded via sonication had the maximum uptake. Moreover, the Exos being loaded with catalase in the presence of saponin exerted higher neuroprotection in vivo.

Another study has also investigated the possibility of utilizing M*ϕ*‐derived Exos without extra targeting moieties for protein delivery to the inflamed endothelium based on the hypothesis that these Exos can attach to the inflamed brain endothelium and pass through the BBB. To this end, Exos were isolated from the M*ϕ*s’ culture media by centrifugation and were characterized. Then, their uptake by human cerebral microvascular endothelial cells (hCMEC/D3) as an in vitro model of BBB was evaluated. After a 4 h lag, Exo accumulation in the cells increased in a linear time‐dependent manner until 48 h. Using inhibitors of different endocytosis pathways, it was revealed that multiple pathways, including clathrin and caveolae‐mediated endocytosis, as well as macropinocytosis, were involved in their uptake. Besides, the involvement of specific carbohydrate‐binding receptors like glucosamine‐binding C‐type lectin was observed in equal extents in both normal and inflamed endothelial cells in vitro. Further in vivo studies also demonstrated that brain accumulation of Exos in the inflamed model was about 5.8 times higher than that of healthy brain 10 min after intravenous injection of the Exos. In the next step, brain‐derived neurotrophic factor (BDNF) was incubated with the Exos and attached to their surface, resulting in particles with a diameter of 209.5 ± 4.7 nm. Intravenous administration of the BDNF‐EXO resulted in a higher accumulation of BDNF in the inflamed brain but not in the healthy brain compared to the naked protein.^[^ ^217^ ^]^

M*ϕ*‐derived ECVs have also been evaluated for delivery of tripeptidyl peptidase 1 (TPP1) to the lysosomal compartment of cells in the brain tissue to treat Batten disease. Three groups of M*ϕ*‐derived ECVs were developed as test formulations: a) ECVs being isolated from M*ϕ*s that had been transfected with the TPP1‐encoding DNA (tECV), b) ECVs that were isolated from naïve M*ϕ*s and loaded with TPP1 via sonication (lECV_sonication_), and c) ECVs that were isolated from naïve M*ϕ*s and loaded with TPP1 via saponin‐mediated permeabilization (lECV_saponin_). The ECVs derived from naïve M*ϕ*s were also used as control (nECV). Five days after the parent cells were transfected, 10^11^ tECVs contained 10 µg of active TPP1, while the amounts of TPP1 in the lECVS loaded with sonication and saponin‐mediated permeabilization were 7 and 5 times higher, about 70 and 50 µg, respectively. Besides, the nECVs revealed to naturally contain about 1 µg of active TPP1. Importantly, tECVs also contained the TPP1 DNA (≈1955 copies per 10^6^ tECVs). Particle size analysis revealed that nECVs, tECV, lECV_sonication_, and lECV_saponin_ groups had a diameter of 106.3 ± 9.3, 133.8 ± 4.1, 130 ± 8, and 135 ± 12 nm, respectively. ECV formulation was revealed to provide sustained release, as well as protecting the TPP1 upon storage and in the presence of degrading enzyme. In an in vitro model of Batten disease, it was observed that lECV_sonication_ and lECV_saponin_ would result in a higher TPP1 accumulation in the damaged cells, compared to the nECVs and tECVs. Meanwhile, the lECV_sonication_ formulation had 1.5 times higher efficiency compared to the lECV_saponin_ formulation. Cell localization analysis further revealed that 1 and 4 h after incubating the ECVs with the PC12 cells as neuron in vitro models, 74.2 ± 15.2% and 68.4 ± 16.7% of the ECVs would be recruited in the lysosomal compartment, respectively. Intraperitoneal administration of the developed formulations in a mouse model of Batten disease revealed that the maximum ECV accumulation in the brain would be 72 h after injection and the ECVs were slowly cleared from the brain over 3 weeks. On the other hand, the free TPP1 molecules administered could hardly be detected in the brain. Comparing the lECV_sonication_ with nECVs with the same treatment protocol, it was also observed that lECV_sonication_ formulation exerts a significant anti‐inflammatory effect, leading to increased animal survival.^[^ ^194b^ ^]^

Since microglia are activated in inflammatory poststroke conditions and exacerbate the brain injury,^[^ ^218^ ^]^ it has been attempted to shift their M1 to M2 phenotype using M*ϕ*‐derived Exos for therapeutic purposes. To this end, M*ϕ*s were first treated with LPS to stimulate the secretion of Exos containing anti‐inflammatory agents (LPS‐Exo). The LPS‐Exos had a diameter of 111.3 ± 10.1 nm and a surface potential of −5.5 mV, while these parameters for Exos from nonstimulated M*ϕ*s (Exos) were reported to be 98.3 ± 9.8 nm and −4.9 mV. The Exos and LPS‐Exos were then co‐incubated with LPS‐stimulated microglial BV2 cells with a 50 or 200 µg mL^−1^ concentration. It was observed that they could decrease the levels of NF‐*κ*B p65, TNF‐*α*, and IL‐6 in the cells, while the LPS‐Exos, specifically with the concentration of 200 µg mL^−1^, exerted a more efficient inhibition compared to the Exos. Furthermore, it was observed that microglial cells take up the LPS‐Exos with an efficiency of 24.5% in 0.5 h, reaching to 67.8% after 4.5 h. Immunofluorescence assay and polymerase chain reaction (PCR) also revealed that when BV2 cells were treated with the LPS, they would present M1 markers, CD80, and iNOS, while when the LPS‐stimulated BV2 cells became exposed to LPS‐Exos, they shifted to M2 phenotype, identified with upregulation of CD206 and Arg1. In a further step, LPS‐stimulated BV2 microglial cells were co‐incubated with SH‐SY5Y neuron cells, which led to an obvious decrease in neuron survival and increase in the expression of apoptosis‐related proteins. On the other hand, when the LPS‐stimulated BV2 cells were treated with LPS‐Exos before being cultured with the neuron, they led to significantly increased viability of the neurons and abated the level of apoptosis‐related proteins. In vivo evaluations on a rat model of ischemia/reperfusion, revealed that LPS‐Exos, following intravenous injection, could significantly penetrate the BBB and specifically home in the infarction lesion. Besides, significant neuroprotection was confirmed in rats receiving LPS‐Exo. Moreover, the number of CD206^+^ cells was significantly higher in the brain of rats receiving LPS‐Exos compared to not‐treated animals, which contained a higher population of CD80^+^ cells, suggesting the M1 to M2 transformation of microglial cells in the presence of LPS‐Exos. Once more, the levels of NF‐*κ*B p65, TNF‐*α*, and IL‐6 in the brain of untreated animals were higher than that of the LPS‐Exo‐treated rats. Altogether, LPS‐Exo treatment culminated in a significantly increased survival of neurons following stroke.^[^ ^219^ ^]^

**Figure 26:**
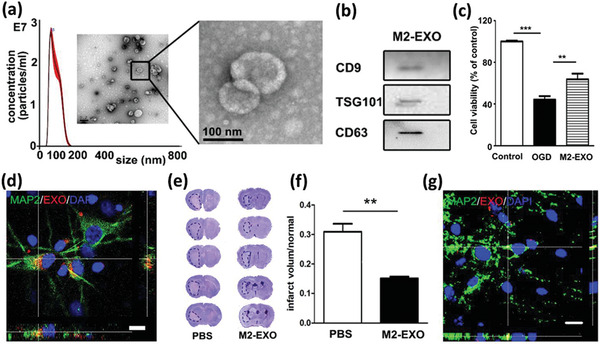
a) Size distribution and morphology of the M2‐EXOs. b) Western blot analysis showed the expression of exosome markers, including CD9, TSG101, and CD63. c) Rate of neurons’ survival being treated with M2‐EXOs after ischemia. d) Confocal imaging demonstrated the uptake of PKH‐26‐labeled exosomes (red) by neurons (green) in vitro. Scale bar is 50 µm. e) Cresyl violet staining of brain sections after 3 days of treatment with PBS and M2‐Exo post‐transient middle cerebral artery occlusion. The infarcted area is shown by dashed line. f) Bar graph of infarct volume normalized to the normal side, confirming that microglial cell‐derived Exos with M2 phenotype can promote neurons’ survival after stroke. g) Confocal image of brain tissue, demonstrating the uptake of PKH‐labeled exosomes (red) by MAP‐2+ neurons. Scale bar = 10 µm. Reproduced with permission.^[^ ^220^ ^]^ Copyright 2019, Ivyspring International Publisher.

In addition to M*ϕ*s‐derived ECVs, those from the microglial cells, as CNS‐residing phagocytes, have also been reported to exert therapeutic effects in a variety of CNS morbidities. They can be utilized alone or as a carrier for anti‐inflammatory agents. Recently, Exos from BV2 microglial cells, which had been pretreated with IL‐4 to present the M2 phenotype (M2‐Exo), have been proposed to protect the mice brain from ischemia/reperfusion injury. In an in vitro model of neurons undergoing oxygen‐glucose deprivation (OGD), M2 BV2 conditioned media seemed to significantly increase neuronal survival, while when the Exo secretion of BV2 cells was blocked by GW4869, such improved survival was not observed. In the next step, Exos were isolated, characterized (**Figure** **26**a,b), and incubated with neurons for 24 h, before the neurons undergo OGD for 45 min. It was observed that after 12 h of reoxygenation, M2‐Exos led to a significant increase in cell viability compared to the control group (Figure 26c). In addition, to examine the uptake of M2‐Exo by neurons, PKH26‐labeled M2‐Exos were incubated with neurons for 24 h and 3D confocal images showed their localization in the cytoplasm of MAP‐2^+^ neurons (Figure 26d). Intravenous administration of M2‐Exos in ischemic mice also led to brain recruitment of the M2‐Exos, and a decrease in neurobehavioral deficits, infarct volume (Figure 26e,f), and neuron apoptosis three days postischemia. In agreement with in vitro data, in vivo confocal imaging also demonstrated the uptake of PKH26‐labeled M2‐Exos by MAP‐2^+^ neurons (Figure 26g). Moreover, knocking down the miR‐124 gene in the parent cells of Exo as well as blocking its downstream target ubiquitin‐specific protease 14 (USP14) in distinct experiments confirmed that miR124 and USP14 played key roles in the antiapoptosis effect of M2‐Exos on the neurons.^[^ ^220^ ^]^

Controlling the inflammatory response of CNS‐residing phagocytes with manipulated ECVs has been also reported as a promising strategy for ameliorating MS. To this end, microglial cells, as brain residing M*ϕ*s, were utilized as the source of ECVs. First, the ECVs were isolated via ultracentrifugation from the BV2 microglial cell culture media, in which the cells had been transfected with IL‐4‐coding DNA or IL‐4‐coding DNA and Mfg‐e8 DNA. It was observed that ECVs form the IL‐4 DNA‐transfected cells contain IL‐4 mRNA and protein, as well as those of IL4‐receptor *α*. Administration of these IL‐4^+^ ECVs induced upregulation of anti‐inflammatory markers, CD206 and arginase 1 (Arg1), in primary microglia and peritoneal M*ϕ*s, and this effect was revealed to happen in a STAT6‐dependent manner in vitro.It was clarified that IL‐4^+^Mfg‐e8^+^ ECVs, with the targeting characteristic provided by Mfg‐e8, were more effectively taken up by myeloid cells and hence, resulted in significantly more IL‐4 delivery compared to the IL‐4^+^ ECVs. Inhibiting lysosomal activity in the recipient cells also significantly increased the IL‐4 delivery and increased the expression of anti‐inflammatory markers ym1 and arg1. In vivo, intravenous injection of ECVs failed to deliver IL‐4 to the CNS, while intracisternal injection allowed them to spread into liquoral spaces, the brain, and the spinal cord until the thoracic region. Intrathecal administration of ECVs led to their interactions with phagocytes that traffic through the CSF. Additionally, IL‐4^+^Mfg‐e8^+^ ECVs, injected intrathecally, could prolong the IL‐4 half‐life in the brain, significantly reduced the clinical and histopathological hallmarks of experimental autoimmune encephalomyelitis (EAE) as a model of MS, and increased the cells with anti‐inflammatory markers arg1 and ym1, while decreasing the cells with the inflammatory marker iNOS. The phenotype transformation of recipient myeloid cells from M1 to M2 was further confirmed by studying the mRNAs associated with the anti‐inflammatory and proinflammatory markers in the myeloid cells and helper TCs isolated from the CNS of the subjects.^[^ ^221^ ^]^ Two other studies also provided evidence for the direct and indirect effect of microglia‐derived ECVs’ glioma cells. In the first report, primary rat microglial cells were isolated from the brain of neonates and purified with an immunomagnetic strategy. After being cultured in the presence and absence of LPS, Exos were isolated and revealed an Exo production efficiency of 27.5 × 10^7^ and 36.1 × 10^7^ Exos per 5 × 10^5^ cells. When the glioma cells were incubated in the presence or absence of Exos from either nontreated or LPS‐treated parent cells, it was revealed that LPS‐Exos significantly reduced the size and invasion of the glioma spheroids. This effect was presumed to be attributed to the miR16 family being transferred by the Exos.^[^ ^222^ ^]^ In the second study, MVs and Exos were distinctly obtained from the microglial BV2 cells and primary mouse microglia, that were pretreated with LPS/IFN‐*γ* or IL‐4. It was observed that LPS/IFN‐*γ* MVs, but not Exos, inhibited the migration of glioma cell in vitro, while the IL‐4 MVs, and not Exos, promoted glioma cells’ migration. On the other hand, LPS/IFN‐*γ* MVs had no direct effect on the viability and proliferation of glioma cells. Nevertheless, in a coculture of microglia and glioma cells in the presence of LPS/IFN‐*γ* MVs, an indirect inhibitory effect on glioma cell viability was observed. One week after engrafting glioma cells to the brain of mice, the LPS/IFN‐*γ* MVs were infused in the tumor region, and one week after, a significant reduction of tumor size was observed, compared to the infusion of IL‐4 MVs that resulted in the increased tumor size. Besides, gene expression of tumor‐associated myeloid cells purified from the tumor tissues, confirmed that LPS/IFN‐*γ* MVs had induced an antitumor phenotype in the TAMs. Complex mRNA content of these MVs also revealed that they were involved in the induction of various inflammatory proteins expression, which led to the significant antiglioma effect.^[^ ^223^ ^]^

Astrocytes are other types of glial cells that play a key role in CNS immune surveillance.^[^ ^224^ ^]^ They have also been reported to regulate phagocytosis activity of microglial cells, and this fact has guided the researchers to exploit astrocyte‐derived ECVs for the treatment of morphine‐induced neurodegeneration. It was reported that morphine treatment stimulates the production of EVs in astrocytes, which can be further taken up by microglial cells and impair their phagocytic activity via TLR7‐NF‐*κ*B‐lincRNA‐Cox2 axis, leading to neurodegeneration. To reverse this phenomenon, morphine‐treated mice intranasally received lincRNA‐Cox2 siRNA‐loaded astrocyte‐derived ECVs. It was observed that they penetrated the brain and were specifically taken up by microglial cells, culminating in the downregulation of lincRNA‐Cox2 in microglia. A significant restoration of phagocytic activity was then demonstrated in the microglia of these mice.^[^ ^225^ ^]^

**Figure 27:**
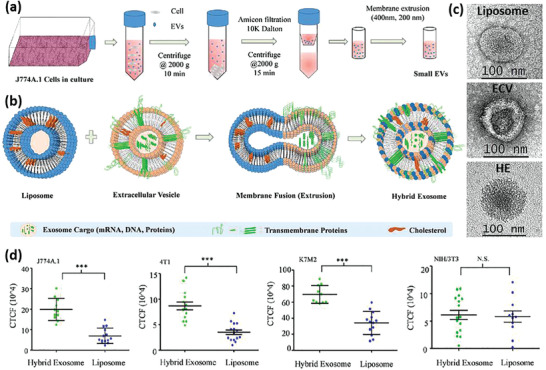
Schematic illustration of the fabrication of hybrid Exo–liposome exosome through a) isolation of small EVs from J774A.1 and b) hybridization of immune cell‐derived small EVs with synthetic liposome using membrane extrusion. c) TEM images of liposomes, ECV, and hybrid Exos (HE). d) Cellular internalization of HE and liposomes in mouse M*ϕ*s, breast tumor cells, mouse osteosarcoma cells, and normal fibroblasts. Internalization was quantified via confocal microscopy according to corrected total cell fluorescence (CTCF). Reproduced with permission.^[^ ^195a^ ^]^ Copyright 2019, Elsevier.

It is worth pointing out that the natural ECVs are not always ideal in their morphology, size, and targeting properties to be used in drug delivery. In this regard, an appropriate strategy to improve the characteristics of ECVs is to hybridize them with synthetic liposomes. A study has reported developing hybridized vesicles based on M*ϕ*‐derived Exos and liposomes based on various anionic, neutral, and cationic lipids. After the Exos were isolated with centrifugation and microfiltration, they were mixed with the liposomes and underwent freeze–thaw cycles. The number of the freeze–thaw cycles was shown to significantly affect fusion efficiency, but no significant difference between the size and morphology of hybrid vesicles and the Exos was detected. Cellular uptake studies demonstrated that being hybridized with anionic and neutral liposomes did not affect exosome uptake efficiency, while the cationic lipids decreased the cellular uptake.^[^ ^226^ ^]^ Another study has compared M*ϕ*‐derived Exos and Exo–liposome hybrids for tumor‐targeted delivery of DOX. To this end, small ECVs were isolated from the culture media via centrifugation and filtration, followed by membrane extrusion to obtain uniform ECVs. The liposomes were also prepared via a thin film hydration technique from egg phosphatidylcholine and cholesterol (66:34 w/w%). Then the ECVs and liposomes were hybridized with a thin film hydration method followed by coextrusion in the ratio of 1:5 ECV protein content: liposome lipid weight, to fabricate the hybridized Exos (HEs). **Figure** **27**a–c illustrates the fabrication procedure and the morphology of the three types of vesicles prepared. The size of liposomes, ECVs and HEs were 131 ± 21 nm (PdI = 0.16 ± 0.05), 139 ± 19 nm (PdI = 0.25 ± 0.02), and 177 ± 21 nm (PdI = 0.19 ± 0.04) and their zeta potential were −31 ± 2, −12 ± 1, and −26 ± 3 mV, respectively. Stability studies revealed that HEs are more stable than small ECVs in terms of diameter and PdI over 30 days. After the hybridization was confirmed through protein assay and fluorescence resonance energy transfer analysis, DOX loading was performed via extrusion in different drug concentrations and 100 µg mL^−1^ was shown to culminate in the highest loading and the least particle aggregation. The pattern of drug release in HEs was similar to that of liposomes in both physiologic and acidic pH, with more drug liberated in the acidic condition (55% vs 83%) in 48 h. In vitro uptake studies on M*ϕ* cell line, osteosarcoma, breast cancer, and mouse fibroblasts revealed that following a 3 h incubation, HEs had a higher internalization in M*ϕ*s, osteosarcoma, and breast cancer cells but not in mouse fibroblasts, compared to the liposomes and ECVs (Figure 27d). Proteinase K assay further demonstrated that proteins of HE had a key role in their increased internalization, compared to the liposomes. IC50 of the HE formulation was similar to free DOX in osteosarcoma and normal fibroblast cells, while it was significantly lower than that of the free drug for breast cancer cells. Ultimately, the HEs revealed increased stability, high drug loading, pH‐sensitive sustained drug release property, significantly improved internalization in the cancer cells, and specific targeting of tumor cells compared to the normal cells.^[^ ^195a^ ^]^

### 5.3 Natural Killer Cell‐Derived Extracellular Vesicles

NKCs are key players of the innate immune system against cancers, applying two distinct strategies, perforin release and inducing caspase‐dependent apoptosis. Based on the fact that Exos inherit the structural and functional properties of their parent cells, a group of researchers has extensively investigated NKC‐derived Exos (NK‐Exo) for cancer treatment.^[^ ^227^ ^]^ Zhu et al. reported separating the NK‐Exos from the culture media of NK92 cells via ultracentrifugation techniques.^[^ ^228^ ^]^ They exhibited a spherical morphology and a diameter of 100–150 nm. Composition analysis of NK‐Exos and NK‐Exos’ membrane being isolated using a membrane protein extraction kit, confirmed that both perforin and FasL, being responsible for the cytotoxic effect of NKCs, were present in the NK‐Exos with a significantly higher concentration than NKCs. Furthermore, NK‐Exos revealed to contain TNF‐*α*. Being cocultured with melanoma cells, it was observed that compared to the NKCs, NK‐Exos exert a higher proliferation inhibition in the tumor cells in a dose‐ and time‐dependent manner.

Using a FasL inhibitor, it was also demonstrated that this protein was engaged in the antitumor activity of NK‐EXOs, as expected, leading to tumor cell death and inhibited proliferation. Finally, intratumoral injection of NK‐Exos in a xenograft melanoma model in mice culminated in a significant decrease of tumor volume by threefold compared to PBS‐treated mice, as well as increased apoptotic cells in the tumor mass. Taking a step forward, based on the studies that had reported increased cytotoxicity of NKCs after being treated with IL‐15, the antitumor activity of ECVs being derived from IL15‐treated NKCs (NK‐ECV_IL15_) was investigated. Primary measurements implied more than twofold increment in both ECV production and ECVs’ protein content for the NKCs following treatment with IL‐15, while the morphology and diameter of the ECVs did not change significantly. Cellular uptake studies further evinced comparable internalization of the NK‐ECVs and NK‐ECV_IL15_ in glioblastoma cells in vitro, while the cytotoxicity of the latter was higher in various concentrations. Similar results were suggested by cytotoxicity analyses on breast cancer and anaplastic thyroid cancer cells. However, no obvious toxicity associated with the NK‐ECV_IL15_ toward normal cells was reported. Intravenous injection of the NK‐ECV_IL15_ and NK‐ECVs in mice exhibited strong tumor targeting and prolonged circulation of the NK‐ECV_IL15,_ while the main accumulation site for both of the vesicles was the liver. Intravenous administration of the ECVs for 8 consecutive days in a mouse model of glioblastoma led to a significant decrease in tumor volume compared to the PBS‐treated group, while the NK‐ECV_IL15_ produced better results (<600 vs ≈900 mm^3^). Similar to previously reported results implying the underlying mechanism for cytotoxicity of the NK‐ECVs, engagement of FasL in that of the NK‐ECV_IL15_, as well as induction of both intrinsic and extrinsic apoptosis pathways were also confirmed.^[^ ^229^ ^]^ The same research group has also compared the natural NK‐Exos and the NK‐Exo mimetic vesicles (NK‐EMV) being fabricated via extrusion of alive NKCs using membrane filters with 5 and 1 µm pore sizes. This method increased the yield of vesicle production by 50‐fold, compared to the natural secretion procedure. The NK‐Exos and NK‐EMVs had a diameter of 118 ± 33.1 and 99.2 ± 2 1.5 nm, respectively. Likewise the NK‐Exos, the NK‐EMVs contained FasL and perforin and revealed to induce both intrinsic and extrinsic mechanisms of apoptosis, meanwhile affecting phosphoinositide 3‐kinase (PI3K) and MAPK signaling pathways, resulting in the proliferation inhibition of the tumor cells. The NK‐Exos and NK‐EMVs had similar cell uptake values. However, in various doses, the superior toxicity of NK‐EMV compared to the NK‐Exos was observed on glioblastoma cells. The superior cytotoxicity of NK‐EMVs was later confirmed during in vitro studies involving breast cancer, thyroid anaplastic carcinoma, and liver cancer cells. Finally, intravenous administration of the NK‐EMVs in a mouse model of glioblastoma indicated their accumulation in the tumor, leading to a significant decrease in tumor size regarding both the volume and the weight.^[^ ^230^ ^]^

## 6 Artificial Immune Cells for Therapeutic Aims

Immune cells are responsible for keeping the body's immunosurveillance. To do their job, they have developed specific mechanisms for cell‐to‐cell adhesion, antigen presentation, mobility and navigation, extravasation and tissue recruitment, cytokine secretion, pathogen and tumor cell detection, scavenging exogenous particles and cell debris, and several other type‐exclusive properties. Within the emerging context of biomimicry, a variety of studies have recently mimicked these characteristics of immune cells to develop more effective therapeutics for hard‐to‐treat disorders.^[^ ^231^ ^]^ The most attempts in this regard have been focused on developing artificial antigen‐presenting cells (aAPCs).^[^ ^232^ ^]^ The aim of designing aAPCs is to activate and expand TCs more vigorously ex vivo and in vivo. Investigating the published material, we have realized that aAPCs could be generally classified into two classes; material‐based (synthetic) aAPCs and nonimmune cell‐based aAPCs. To develop synthetic aAPCs, usually a ligand for TCR (signal 1) and a co‐stimulatory molecule (signal 2) would be anchored on the surface of micro‐ and nanostructures, and cytokines (signal 3) could be incorporated within the aAPCs as well. On the other hand, cells rather than immunocytes could be genetically or nongenetically engineered to present the stimulatory molecules required for the activation and expansion of TCs.

**Figure 28:**
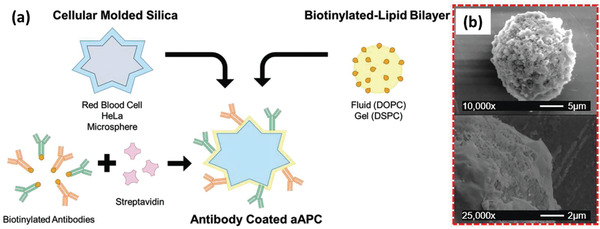
a) Schematic depiction of preparing cell‐templated aAPC using red blood cells or HeLA cells. Cellular molded silica particles were further coated with lipid 1,2‐dioleoyl‐*sn*‐glycero‐3‐phosphocholine (DOPC) that possesses a fluid‐like state at physiological temperatures or 1,2‐distearoyl‐*sn*‐glycero‐3‐phosphocholine (DSPC) that has a gel‐like state at physiological temperatures. The particles were surface conjugated with anti‐CD3*ε* and anti‐CD28 Abs through biotin–streptavidin reaction. b) SEM images of HeLa molded silica microparticles. Reproduced with permission.^[^ ^234^ ^]^ Copyright 2018, Wiley‐VCH.

Physicochemical characteristics of the synthetic aAPCs, including shape, size, flexibility, and the density of targeting ligand on the surface can determine the efficiency of them for activation, differentiation, and proliferation of the TCs.^[^ ^233^ ^]^ To tune these physicochemical properties, multifarious biomaterials have been utilized to develop aAPCs. For example, e mesoporous silica microparticles with different shapes and sizes were prepared using erythrocytes and HeLa cells as templates. Then, liposomes of various compositions and fluidity, with different molar ratios of loaded biotin for further Ab attachment, were developed as the membrane for the silica particles (**Figure** **28**a,b). To envelope the silica particles with the liposomes, they were incubated in a 1:1 mass ratio at room temperature and underwent intermittent mixing by inversion. Next, biotinylated anti‐CD3*ε* and anti‐CD28 Abs with different molar ratios (0:1, 1:5, 1:1, 5:1, and 1:0 of anti‐CD3*ε*‐to‐anti‐CD28) were docked on the lipid membrane of the silica particles via streptavidin linker. Overall, 18 different types of aAPCs were fabricated and analyzed after co‐incubation for 72 h with primary human TCs. Expression of both the TC activation marker (4‐1BB) and Th1 cytokines (IFN‐*γ*, IL‐2, and TNF‐*α*) were found to be dependent on the density of Ab and membrane fluidity. The results revealed that increased Ab density and membrane fluidity have the most significant effects on the TCsʼ expansion. Meanwhile, nonspherical particles with increased membrane fluidity shifted the helper TC/cytotoxic TC ratio to a more equal state, which is favorable in case of working with CAR‐TCs. Nonspherical platforms with increased Ab density also led to larger populations of less differentiated TC phenotype.^[^ ^234^ ^]^

**Figure 29:**
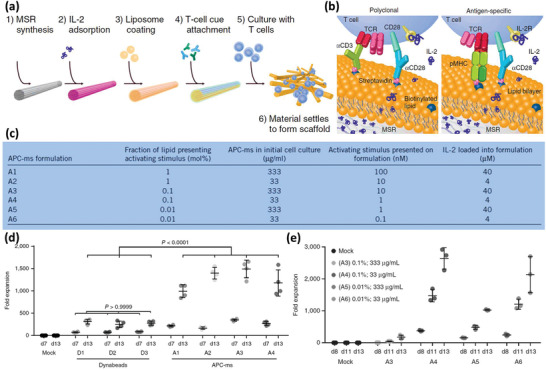
a) The process of APC‐ms formation from the MSRs. b) For polyclonal T cell expansion, *α*CD3 and *α*CD28 were attached on the surface of the SLB‐MSR particles (left), while for antigen‐specific T‐cell expansion, pMHC and *α*CD28 were attached (right). c) Summary of APC‐ms formulations used for the TC expansion studies shown in panels (d) and (e) of the figure. d) Polyclonal expansion of primary mouse TCs that were either untreated (mock), or cultured with Dynabeads or APC‐ms. The effect of Dynabeads of varying doses (D1, D2, D3), or APC‐ms of varying formulations (A1, A2, A3, A4) were tested on TC expansion at various time points (day 7 and day 13 of the cotreatment). As for Dynabeads, the tested bead‐to‐cell ratios were 1:1 (D1), 5:1 (D2), and 25:1 (D3). e) Antigen‐specific expansion of primary mouse CD8^+^ TCs that were either untreated (mock), or cultured with various APC‐ms formulations (A3, A4, A5, A6; see panel (c) of the figure). Reproduced with permission.^[^ ^235^ ^]^ Copyright 2018, Springer Nature.

Another APC‐mimimicking system based on silica particles has also been established for the polyclonal expansion of TCs. To this end, silica microrods (MSRs) (≈70 µm length, ≈4.5 µm diameter, and pore size of 10.9 nm) were prepared and was loaded with IL‐2. Then, liposomes containing biotinylated lipids were prepared via a thin film‐rehydration method and then extruded to obtain monodisperse 140 nm vesicles. Liposomes and MSR were incubated together in the next step with a 1:4 mass ratio for 1 h to form supported lipid bilayer‐silica microrods (SLB‐MSR). Finally, activating Abs against CD3 (*α*CD3) and CD28 (*α*CD28) for polyclonal T‐cell expansion or peptide‐loaded major histocompatibility complex (pMHC) and *α*CD28 for antigen‐specific T‐cell expansion were conjugated on the surface of the SLB‐MSR via streptavidin linkers (**Figure** **29**a,b).^[^ ^235^ ^]^ SLB‐MSR particles functionalized with TC activation cues could spontaneously form antigen presenting cell‐mimetic scaffolds (APC‐ms) in the cell culture. Like natural APCs, these APC‐ms presented both stimulatory and co‐stimulatory molecules to TCs and showed first‐order kinetic for IL‐2 release to infiltrate TCs in to the empty spaces of the scaffold.

In the defined experiment conditions (13 days) on the polyclonal expansion of primary mouse TCs, APC‐ms led to three‐ to fivefold greater TC expansion compared to Dynabeads (Figure 29c,d), while they both induced the same levels of exhaustion markers in the TCs. Another significant difference between the resulted TC populations was the CD8/CD4 ratio, in which APC‐ms caused CD8‐skewness and Dynabeads caused CD4‐skewness. When the same dose of anti‐CD3 and anti‐CD28 was presented to the TCs by either APC‐ms or Dynabeads, APC‐ms exerted more proliferation induction within 7 and 13 days.

As for the antigen‐specific expansion of primary mouse T cells, anti‐CD3 Ab was replaced with a biotinylated H‐2K(b) MHC class I monomer presenting SIINFEKL to evaluate the efficiency of APC‐ms to expand mouse monoclonal CD8 TCs. The results showed robust expansion of CD8^+^ TCs for different formulations of the APC‐ms (Figure 29c,e). In a further in vitro study, the resulted TCs could recognize the antigen on the melanoma cells and destroy them. The TCs being primed with the APC‐ms or commercial microbeads (Dynabeads; microspheres functionalized with *α*CD3 and *α*CD28) revealed similar antitumor efficiencies in vivo on a xenograft model of Burkitts’ lymphoma. However, the former one can be considered as a desirable alternative for the Dynabeads because the context, in which Dynabeads present three critical signals to TCs is not representative of how they are naturally presented by APCs. This can lead to T cell products with limited or dysregulated functions and suboptimal T‐cell expansion rates. Moreover, Dynabeads are nondegradable and they must be separated from the cell product before infusion, increasing cost and manufacturing challenges.

Another class of materials being used in the development of aAPCs is magnetic particles, which enable the aAPCs to also be utilized in the isolation of specific cells. A group of researchers has reported the attachment of signal 1 (MHC‐Ig dimer) and 2 (anti‐CD28 Ab) on the surface of dextran‐coated paramagnetic iron oxide NPs with a diameter of 50–100 nm. Polyclonal CD8^+^ TCs were concentrated from the mouse spleen and lymph node homogenate using a commercial CD8 magnetic enrichment column. The CD8‐enriched lymphocytes were then mixed with the antigen‐specific aAPCs and further passed through a magnetic enrichment column. The negative fraction was discarded and the antigen‐positive fraction was cultured in an appropriate medium to expand the cells. The purification efficiency seemed to be aAPC dose‐dependent and the developed nanosystem could increase the purity of the antigen‐specific TCs by more than tenfold. Also, 1000–10 000‐fold TC expansion was obtained in distinct experiments, which is comparable to the in vivo CD8^+^ TC expansion in response to viral infections. In vivo studies on a melanoma model ultimately confirmed the superior antitumor efficiency of the TCs being enriched and expanded using the developed aAPCs, concerning both the tumor size and animal survival as compared to the control groups.^[^ ^236^ ^]^ SPIONs have also been utilized for investigating the effect of aAPCs’ size on the TC activation and expansion rate.^[^ ^237^ ^]^ The aAPCs were developed by conjugating chimeric peptide‐loaded major histocompatibility complex (pMHC‐Ig), containing the model SIY antigen (Signal 1) and anti‐CD28 Ab (Signal 2) on the surface of SPIONs with average diameters of 50, 300, 600, and 4500 nm. In vitro studies revealed that particles larger than 300 nm activate the TCs more efficiently than those with a 50 nm diameter. Zhang et al. have investigated the effect of the surface texture of aAPCs on TC activation, using smooth or coarse Fe_3_O_4_‐loaded magnetic poly(l‐lactide) (PLLA) microspheres.^[^ ^238^ ^]^ To prepare the aAPCs, PLLA microspheres with a ≈2.1 µm diameter were fabricated via a single emulsion‐solvent evaporation method and three bilayers of PAH/bovine serum albumin (BSA) or PAH/silk fibroin were coated on the particles. Being treated with THF, a wrinkled surface with a higher surface/volume ratio was created, which allowed docking of more signal 1 (anti‐CD3 Ab) and signal 2 (anti‐CD28 Ab) on each particle compared to the particles with a smooth surface, as well as multivalent interaction with the TCs, leading to a higher TC activation efficiency. In vitro studies unraveled a ten time increase in interaction between the particles with coarse surface and the TCs compared to that of the smooth‐surface particles.

Due to the importance of multivalent interaction of aAPCs with TCs for their activation and expansion, a group of researchers has worked on semiflexible filamentous polymeric structures that allowed the attachment of signal 1, signal 2, and other stimulating agents. To this end, three different types of polyisocyanopeptide copolymer (PIC) were synthesized by changing the polymer length and the number of anti‐CD3 Abs (*α*CD3). The azide group was utilized for the attachment of bicyclononyne (BCN)‐functionalize streptavidin, which further facilitated the conjugation of the biotinylated *α*CD3 (signal 1) to the polymeric backbone. To evaluate the effect of the length of these aAPCs on the TCs activation, P1 and P2 polymers with the length of 175 and 350 nm were synthesized, while attempting to maintain equal signal 1 density on them. Hence, the P1 molecule carried 1–2 *α*CD3 and the P2 molecule about 3 *α*CD3. Treating peripheral blood lymphocytes with the developed aAPCs demonstrated that in short‐term, P2 induced TC activation in smaller amounts, and in mid‐term, it led to stronger TC activation. To evaluate the effect of the density of signal 1 conjugated to the polymer on the TCs activation, polymers with the same length of 400–450 nm were synthesized, carrying 3–4, 5, or 10–11 *α*CD3 per molecule (P3a, P3b, P3c, respectively). P3c induced more vigorous TC activation compared to the free anti‐CD3 Ab, P3a, and P3b. Indeed, the density of anti‐CD3 Ab demonstrated a positive correlation with the amount of IFN‐*γ* released by the TCs, as a marker of their activity.^[^ ^239^ ^]^ In another study, it was shown that when both anti‐CD3 Ab and IL‐2 were loaded on the same PIC backbone, they would induce stronger TC activation, compared to administration of the two stimulants being loaded on separate backbones or exploiting free IL‐2 along with the *α*CD3 conjugated PIC.^[^ ^240^ ^]^

PLGA particles, due to their desirable biodegradability and biocompatibility, have extensively been used for developing aAPCs.^[^ ^241^ ^]^ Meyer et al. have investigated the effect of aAPC shape on their TC expansion efficiency by exploiting PLGA‐based aAPCs.^[^ ^242^ ^]^ To this end, spherical and ellipsoidal PLGA NPs with the same volume were fabricated and signals 1 and 2 complexes were anchored on the surface of each group of the particles. In vitro studies revealed that ellipsoidal particles induce more TC activation as a result of enhanced multivalent TCR binding and TCR clustering, which the ellipsoidal particles provided due to their more planar shape. Another advantage that ellipsoidal particles revealed was their reduced uptake by M*ϕ*s, leading to their prolonged circulation and higher maximum serum concentration in vivo. When either of the particles along with the TCs were administered concurrently in vivo, a significant increase in the expansion of antigen‐specific cytotoxic TCs was observed in the mice receiving ellipsoidal aAPCs compared to those receiving the spherical NPs. Similarly, a study has recently reported even more prolonged circulation and more vigorous in vitro and in vivo TC activation in response to PEGylated ellipsoidal PLGA NPs.^[^ ^243^ ^]^

Several studies have also taken advantage of PLGA‐based aAPCs combined with other strategies to enhance TC activation. For instance, the effect of PLGA‐based aAPCs along with a checkpoint inhibitor (anti‐programmed cell death protein 1 (PD‐1) Ab) on TC activation and proliferation was investigated.^[^ ^244^ ^]^ PLGA particles were fabricated and then the melanoma antigen‐loaded MHC‐IgG dimer (signal 1) and anti‐CD28 Ab (signal 2) were anchored on the surface of particles by EDC–NHS chemistry. The particles had a 4–5 µm diameter and their surface‐anchored protein content indicated an incubation time‐dependency. It was revealed that the combined therapy by aAPCs and anti‐PD‐1 Ab significantly increased the activation of antigen‐specific cytotoxic TCs in vitro and a synergistic effect between the aAPCs and anti‐PD‐1 Ab was declared, leading to significantly increased production of IFN‐*γ* by the treated TCs. In a mouse model of melanoma, dual therapy with antigen‐specific TCs co‐incubated with aAPCs and injection of anti‐PD‐1 Ab resulted in a significant increase in the activation of the TCs and increased animal survival. This superior in vivo effect could be partly due to the neutralization of tumor‐induced modulatory effect with the aAPCs. In another example of using aAPCs along with other strategies of TC activation, Zhang et al. have reported developing IL‐2 and anti‐cytotoxic T‐lymphocyte‐associated antigen 4 (CTLA4) Ab‐loaded PLGA microparticles, with a diameter of about 4.5 µm, spherical shape, and smooth surface, to concurrently present an activating signal and anti‐inhibitory signal to TCs.^[^ ^245^ ^]^ The particle's surface was then activated by EDC–NHS chemistry and PEI was ultimately conjugated to it. The encapsulation efficiency was about 80% independently for each of the molecules. Their drug release efficiency over 28 days was 81% and 92% for IL‐2 and anti‐CTLA4 Ab, respectively, showing a sustained drug release manner. To upgrade the drug‐loaded PLGA particles to aAPCs, an antigen‐MHC complex, as well as anti‐CD28 Ab, were anchored on the surface of the particles. Co‐incubation of the IL‐2 and anti‐CTLA4 Ab‐loaded aAPCs with splenocytes for 7 days significantly activated and expanded splenocytes to produce a large population of antigen‐specific cytotoxic TCs, larger than those being incubated with aAPCs containing only IL‐2 or anti‐CTLA4 Ab. Then the splenocytes were harvested and cocultured with the target melanoma cells, leading to significantly increased cytotoxicity compared to the control groups. To evaluate the efficiency of the developed particles in vivo, the tumor cells and the developed aAPCs were injected into the mice simultaneously. After 28 days, the mice receiving the IL‐2 and anti‐CTLA4 Ab‐loaded aAPCs demonstrated significantly smaller tumor size as well as increased survival, compared to the groups being treated with IL‐2 or anti‐CTLA4 Ab‐loaded aAPCs. This study clearly illustrated the capability of PLGA particles to act as both aAPC and macromolecular therapeutics carriers.

In a further step, the same research group developed a similar PEI‐coated PLGA particles as aAPCs, carrying a combination of eleven immune molecules, including two antigens (H‐2Kb/TRP2180‐188‐Ig dimers and H‐2Db/gp10025‐33‐Ig dimers), two co‐stimulatory molecules (anti‐CD28, anti‐4‐1BB, and anti‐CD2), and one self‐marker (CD47‐Fc) onto the surface, while two cytokines (IL‐2 and IL‐15), one chemokine (C‐C motif chemokine ligand 21 (CCL21)), and two checkpoint inhibitors (anti‐CTLA4 and anti‐PD‐1) within the particles to maximize the effect of aAPCs.^[^ ^232a^ ^]^ In the defined experimental setting, all of the incorporated molecules had loading efficiencies of 81–88.3%, comparable to when only one type of molecules was loaded within the same particles. Incubating spleen lymphocytes or antigen‐specific CD8^+^ TCs with the developed aAPCs resulted in the induction of ≈270 and ≈330 times more expansion in the cells, respectively. Moreover, the cytotoxicity of the spleen lymphocytes being incubated with the aAPCs against melanoma cells increased by ≈810 times (lymphocyte:tumor cell ratio of 50:1). Administering the aAPCs at the same time with tumor inoculation in mice led to a supplementary significant increase in the population of antigen‐specific CD8^+^ TCs in the peripheral blood, decreased tumor size, and improved animal survival. Biodistribution studies further revealed the colocalization of the aAPCs not with the tumor, but with the spleen and lymph nodes, where TCs would home. Altogether, these results confirmed superior activation of TCs as a result of therapy with multifunctional aAPCs, resembling the natural APCs.

The PEI‐coated PLGA‐based particles were further exploited to develop a different subclass of synthetic aAPCs, called killer aAPCs (KaAPCs). These particles take advantage of the antigen‐presenting mechanism to target the pathologically reactive TCs in autoimmune diseases or tissue engraftment and kill TCs via inducing apoptosis signaling pathways. A group of researchers have reported attaching MHC I‐IgG dimer along with anti‐Fas monoclonal Ab on the PEI–PLGA particles and injecting it to mice receiving alloskin transplantation in the 9th day, 11th day, and 13th day postengraftment. It was observed that these alloantigen presenting KaAPCs could increase the survival of the alloskin from ≈20 days in the control groups to more than 60 days in the KaAPC‐treated mice. Histological evaluations on the alloskin of these treated mice revealed a sharp decline in the population of alloreactive TCs, decreased infiltration of CD8 (but not CD4) TCs to the alloskin graft, and significantly reduced inflammation. Additionally, it was observed that the population of alloreactive TCs in the peripheral blood and spleen was decreased. Importantly, the developed KaAPCs were well tolerated, neither killing the nonpathologic immune cells nor impairing the overall immune system of the host.^[^ ^246^ ^]^ In another attempt, the micrometer‐sized KaAPC platform was exploited to modulate both CD4 and CD8 pathologic TCs in a mouse model of EAE. To this end, PLGA–PEI microparticles, encapsulating the inhibitory transforming growth factor‐beta (TGF‐*β*) cytokine were prepared and decorated with antigen‐MHC complexes, regulatory molecules including anti‐Fas Ab, PDL1‐Fc, and the self‐marker CD47‐Fc. The developed KaAPCs were injected to the EAE mice intravenously, intraperitoneally, or subcutaneously and the most successful was found to be the intravenous route. Intravenous administration of the KaAPCs based on a defined schedule ameliorated the CNS inflammation. Although the KaAPCs could not pass through the BBB, they were recruited in the lymphoid organs, destroying the antigen‐specific autoreactive CD4 and CD8 TCs. They also induced expansion of regulatory TCs, increased the expression of inhibitory cytokines, while limiting the secretion of inflammatory molecules in the splenocytes.^[^ ^247^ ^]^

**Figure 30:**
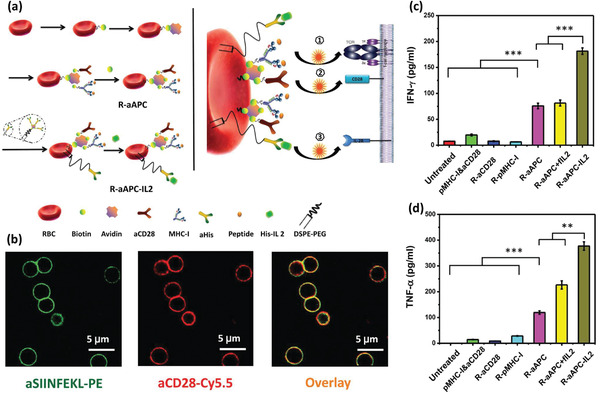
a) Scheme of preparing RBC‐based aAPC modified with pMHC‐I, aCD28, and IL‐2, as well as their role in activating CD8+ T cells. b) Confocal images of RBC‐based aAPCs labeled with fluorescent probes. PE green fluorescence indicated anti‐OVA257–264 (SIINFEKL) Ab labeled pMHC‐I and Cy5.5 red fluorescence showed aCD28. The scale bar is 5 µm. c,d) Quantification of IFN‐*γ* and TNF‐*α* release from the untreated T cells or T cells treated with free pMHC‐I and aCD28 (pMHC‐I&aCD28), T cells treated with RBC‐aCD28 (R‐aCD28), T cells treated with RBC‐pMHC‐I (R‐pMHC‐I), T cells treated with R‐aAPC without IL‐2 (R‐aAPC), T cells treated with R‐aAPC plus free IL‐2 (R‐aAPC + fIL‐2), and T cells treated with IL‐2 conjugated R‐aAPC (R‐aAPC‐IL‐2). Error bars were based on standard deviation (SD) of triplicate samples and *p* values represent *p < 0.05, ^**^ *p* < 0.01, ^***^ *p* < 0.001, and ^****^ *p* < 0.0001. Reproduced with permission.^[^ ^249^ ^]^ Copyright 2017, Wiley‐VCH.

The second class of aAPCs is cell‐based systems. A variety of cells with various origins have been exploited for this purpose.^[^ ^248^ ^]^ The first example refers to the RBCs, being nongenetically engineered. Their merits for being exploited for biomedical applications include high biocompatibility, biodegradability, prolonged circulation, large surface area, and membrane flexibility, which are favorable for a particulate drug carrier and an aAPC.^[^ ^249^ ^]^ Sun et al. have reported inserting signal 1 (MHC–antigen complex) and signal 2 (anti‐CD28 Ab) via biotin–avidin interaction on to the RBCs’ lipid membrane. Signal 3 (IL‐2) was also anchored on the surface via attaching the His‐tagged IL‐2 to an anti‐His Ab‐conjugated 1,2‐distearoyl‐*sn*‐glycero‐3‐phosphorylethanolamine‐polyethylene glycol (DSPE‐PEG) (**Figure** **30**a). The attachment of the signals was confirmed using confocal microscopy imaging (Figure 30b), while it was observed that their attachment did not impair the stability of RBCs in fetal bovine serum or PBS containing 10% serum for 48 h. When the aAPCs were cocultured with the splenocytes, strong activation and proliferation of the splenocytes resulted in a large population of antigen‐specific CD8 TCs, secreting a significant amount of IFN‐*γ* and TNF‐*α* (Figure 30c,d). The activity level of TCs being pulsed with aAPCs was found to be much higher than that of TCs being pulsed with RBCs only containing signal 1 or signal 2, or receiving IL‐2 as free molecules instead of being attached to the RBC surface. Next, to evaluate the efficiency of the aAPCs in TCs to combat against tumor cells, melanoma cells with or without the aAPCs’ associated antigen were cocultured with naïve or aAPC‐pulsed splenocytes. It was demonstrated that the aAPC‐treated splenocytes led to antigen‐specific tumor cell lysis.

Besides nongenetic engineering methods, various genetic modifications have also been employed to several cell types for transforming them into aAPCs. Garnier et al. have reported genetically engineering fibroblast‐derived cells to express human leukocyte antigen‐DR (HLA‐DR) isotype, co‐stimulatory B7.1, and ICAM‐1 and LFA‐3 adhesion molecules.^[^ ^250^ ^]^ Three types of cellular aAPCs were developed by guiding the cells to express HLA‐DR1, HLA‐DR15, or HLA‐DR51, being loaded with influenza virus hemagglutinin‐derived antigen, myelin basic protein, or factor VIII, respectively. In coculture tests, it was observed that the aAPCs could take up a whole protein, process that, and present the proper epitope to the CD4 TCs, leading to TC activation at an even greater level of activation than with the natural professional APCs. Besides, these aAPCs could reactivate and amplify a high number of specific memory CD4^+^ T cells, introduced as promising tools for adoptive CD4 TC therapy.

In another study, the cellular platform exploited for developing aAPC was a type of human B cell‐lymphoma, expressing high levels of HLA‐A2 and TC co‐stimulatory molecules, including CD80 and CD86. These cells were genetically engineered to also express alphafetoprotein_158‐166_ (AFP_158‐166_) peptide and IL‐15. It was observed that the aAPCs present the AFP_158‐166_ peptide in complex with the HLA and produced IL‐15 at a higher level compared to the DCs. Being cocultured with the cytotoxic TCs, it was revealed that the aAPCs presented the antigen to the TCs with a higher density than the DCs being pulsed with the AFP_158‐166_ peptide and led to more expansion of antigen‐specific CD8 TCs. The number of CD8 TCs secreting IFN‐*γ* was significantly higher in the population treated with the aAPCs as compared to DCs. Moreover, in vivo studies showed TCs being pulsed with the aAPCs expanded more efficiently and had very high tumor infiltration.^[^ ^251^ ^]^

Another cellular platform that has been widely investigated for developing aAPCs is K562 erythroleukemia cells. These cells do not express HLAs but contain adhesion molecules like ICAM‐1 and LFA‐3.^[^ ^252^ ^]^ Shao et al. have reported genetic engineering of these cells to express HLA, CD80, and 4‐1BBL and incubated them with tumor antigens to generate aAPCs.^[^ ^253^ ^]^ The developed aAPC or DC cells treated with the same antigens were cocultured with the cytotoxic TCs in comparable ratios and it was revealed that aAPCs induced more expansion in antigen‐specific TCs than the DCs. The population of TCs being activated via aAPCs contained more effector memory TCs highly expressing CD27 compared to the TC population being activated with DCs. In vitro tests confirmed a higher level of IFN‐*γ*, TNF‐*α*, and IL‐2 production and a decreased level of IL‐6 and IL‐12 secretion in TCs being pulsed with aAPCs, implying the improved potential of antitumor cellular immunity response as compared to TCs treated with DCs.

An important indication of aAPCs would be in the context of CAR‐TC therapy, where there is a significant demand for efficient methods of CAR‐TC expansion, while maintaining their functionality. In this regard, Sahin et al. have reported developing first generation (G1) and third generation (G3) CAR‐TCs against an antigen of glioblastoma multiforme (EGFRvIII), as well as two cell‐based aAPCs.^[^ ^254^ ^]^ The aAPCs were comprised of K562 erythroleukemia cells being genetically engineered to express the antigen (EGFRVIIIΔ654 aAPC) or to express the antigen and co‐stimulatory molecules CD32, CD80, and CD137L (CD32‐80‐137L‐EGFRVIIIΔ654 aAPC). When the G3 CAR‐TCs were co‐incubated with either of the aAPCs, it was observed that in the short‐term (3 weeks), CD32‐80‐137L‐EGFRVIIIΔ654 aAPC induced a significant increment in the expansion of TCs. Besides, in the long‐term coculture of various CAR‐TCs with either of the aAPCs in the presence of IL‐2 and hygromycin, it was confirmed that CD32‐80‐137L‐EGFRVIIIΔ654 aAPCs led to the significant proliferation of G3 CAR‐TCs, while the TCs remained active and viable. Moreover, the G3‐CAR‐TCs being treated with CD32‐80‐137L‐EGFRVIIIΔ654 aAPC produced the highest level of IFN‐*γ* in vitro. Injecting the G1 and G3 CAR‐TCs being treated with CD32‐80‐137L‐EGFRVIIIΔ654 aAPC to the tumor‐bearing animals revealed that G3 CAR‐TCs significantly prolonged animal survival. Besides, histological analyses revealed that G3 CAR‐TCs had an extended survival within the tumor tissue, while remaining active. Altogether, the results implied that the developed CD32‐80‐137L‐EGFRVIIIΔ654 aAPCs significantly increased the expansion and activity of the G3 CAR‐TCs and after in vivo administration, the TCs remained viable and active in the tumor milieu.

**Figure 31:**
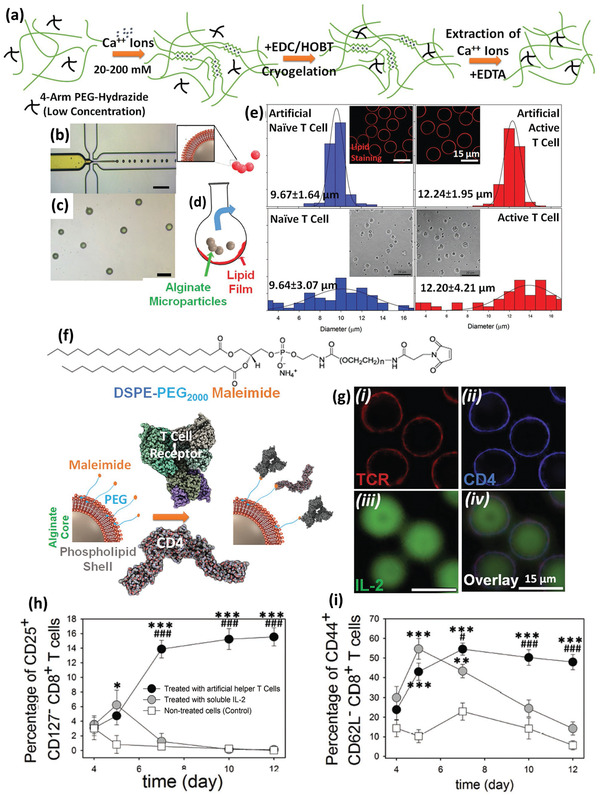
a) Schematic illustration of the synthesis of alginate‐based microparticles. b) Microfluidic‐based production of alginate microparticles; scale bar: 100 µm. c) Optical image of the alginate microparticles. d) Hydration of preformed POPC lipid film with alginate particles could create lipid‐coated alginate microparticles to mimic cells. e) Comparing the size of microparticles at different flow rates (upper panels) with natural naïve and active T cells (lower panels). Gaussian distributions are plotted to elucidate the average sizes and standard deviations (*n* > 50). Insets: Fluorescent confocal images of lipid‐coated alginate microparticle, in which DiD red fluorescence dye was used for phospholipid coating. f) Maleimide‐thiol chemistry was used to functionalize DSPE‐PEG2000‐modified phospholipid shell on the surface of the particles with TCR and CD4. g) Fluorescent confocal images of microparticles, showing i) TCR, ii) CD4, iii) encapsulated IL‐2, and iv) the overlay image; scale bar is 15 µm. h) Percentage of cells that fall in the category of effector cells (CD25^+^, CD127^−^), obtained by flow cytometric analysis when the cells were cocultured with microparticles at different time points. i) Percentage of effector cells (CD44^+^ CD62L^−^), obtained by flow cytometric analysis when the cells were cocultured with microparticles at different time points. Adapted with permission.^[^ ^255^ ^]^ Copyright 2018, Wiley‐VCH.

In addition to mimicking the antigen‐presenting properties of the immune cells, several other characteristics of these cells, including their mechanical properties, navigation potential, stealth, cytokine secretion, and antitumor activity have been imitated in artificial immune cells. For example, a group of researchers has introduced a hydrogel‐based engineered platform, emulating TCs regarding many aspects from mechanical properties to their migration and cytokine secretion. However, the study was mainly focused on the mechanical stiffness of these platforms as model DDSs and the effect of the flexibility on the overall behavior and efficiency of these systems. To this end, a microfluidic droplet generator approach was used to produce alginate microparticles (**Figure** **31**a–e). The alginate solution was in the central flow and mineral oil plus Span 8% was used as sheath flow to focus the polymer flow. In the constant fraction of 4‐arm PEG hydrazide as a crosslinker in the main channel, the concentration of the alginate and calcium chloride was optimized to obtain particles with variable, but physiologically relevant elasticities similar to naïve and active TCs. By altering the ratio of sheath to central flow, the final particles were produced with a diameter of ≈9 or ≈12 µm to resemble the size of naïve and active TCs, respectively. Mechanical stiffness of the particles was adjusted by chemical crosslinking after microfluidic particle synthesis and the calcium ions were extracted from the particle structure to increase its flexibility. Then, the alginate particles were cloaked in a lipid bilayer envelope made from 1‐palmitoyl‐2‐oleoyl‐*sn*‐glycero‐3‐phosphocholine (POPC) via the film hydration technique to mimic the lipid bilayer cell membrane. Next, maleimide‐functionalized DSPE‐PEG was anchored on the lipid bilayer (Figure 31f) to facilitate conjugation of CD4 Ab and TCR protein with a concentration similar to that of CD4 and TCR on the TCs. The softness of the optimum particles was confirmed by passing through capillaries with diameters half of the particles’ size and recovered their shape and size when exiting the capillaries. Pharmacokinetic studies revealed that the softer particles had significantly greater elimination half‐life compared to the stiffer ones (4 vs 1 h). In the next step, 100 nm SPIONs were embedded within the particles and magnetic guidance was used to imitate the chemotaxis of the TCs. In a transwell migration study using membranes with the pore sizes of 5 and 8 µm, both soft and stiff particles revealed increased migration in the presence of a magnetic field, while the stiffer particles struggled to pass through 5 µm pores. To evaluate drug release from the developed particles, dextran with various molecular weights and IL‐2 were distinctly loaded within the particles (Figure 31g). A size‐dependent sustained release pattern was revealed for dextran. On the other hand, it was revealed that in the presence of a magnetic field, the release of IL‐2 from the SPION‐embedded particles would increase, implying the elastic deformation of the particles’ microstructure in the presence of the magnetic field. Finally, the IL‐2‐loaded CD4 TC‐mimicking particles were used to activate CD8 TCs in co‐incubation studies. Particle‐treated TCs had significantly higher survival compared to those treated with free IL‐2 or negative controls. Besides, cytotoxic TCs treated with the artificial helper, TCs expressed a higher level of effector cells’ markers. For example, the cells that were treated with IL‐2‐releasing particles could encourage the formation of effector cells (CD25 high, CD127 low). In addition, since a low level of CD62L (L‐selectin) expression is another indicator of effector cells, its expression was tracked and an ascending number of effector cytotoxic T lymphocytes (CD44high, CD62L) was observed (Figure 31h,i). Finally, it was observed that TCs being pulsed with the artificial helper TCs had significantly increased antitumor activity compared to the control groups.^[^ ^255^ ^]^

The same research group has also developed artificial helper TCs liberating cytokines in a controlled fashion. Heparin and alginate were first attached covalently because IL‐2 that was further loaded into the particles had a greater tendency to be retained in the presence of heparin. They then used the previously mentioned microfluidic device to produce alginate–heparin particles with various diameters, while the concentrations of polymer and calcium ions were modified to obtain particles with diverse porosity. Next, different amounts of IL‐2 were co‐incubated with the alginate or alginate–heparin particles and it was confirmed that alginate–heparin particles can encapsulate more IL‐2. Additionally, another microfluidic setting was developed to coat the particles with chitosan at diverse thicknesses to tune the release rate of IL‐2. Harnessing this strategy, the IL‐2 release pattern could be tuned by mixing the coated and uncoated particles. To test the effect of the developed artificial helper TCs on the activation of cytotoxic TCs, the cells were co‐incubated with coated and uncoated particles and it was observed that with the sustained IL‐2 release the particles provide, the viability of the cells would be comparable to when fresh free IL‐2 was added to culturing media continuously. Besides, controlling the IL‐2 concentration by modifying the ratio the cell number to the coated, uncoated, or the mixture of coated and uncoated particles revealed that exposure to a higher level of IL‐2 would guide the TCs toward an effector phenotype, while lower IL‐2 concentrations led to a memory‐like phenotype. In addition, with more IL‐2 available in the culture medium, more granzyme and perforin was generated by the TCs. The developed particles further revealed a great potential to encapsulate positively charged proteins, such as stromal cell‐derived factor 1 alpha (SDF1*α*), TGF‐*β*, and IFN‐*γ*, but not the negatively charged proteins like TNF‐*α*. Transwell migration studies demonstrated that SDF1*α*‐loaded particles could induce TC migration. Besides, when TGF‐*β*‐encapsulated particles and IL‐2‐containing particles were used together, they could transform naïve CD4^+^ TCs to the regulatory phenotype. Altogether, the results showed that the developed artificial immune cells could affect the phenotype and function of the TCs.^[^ ^256^ ^]^

Blocking the PD1 checkpoint inhibitor by anti‐PD1 Abs has been an effective strategy to treat various malignancies, although there are still concerns about the off‐target effects of using high‐affinity Abs and the development of autoimmune diseases. Mukundan et al. have recently introduced a novel TC‐mimicking microparticle to combat PD1 signaling.^[^ ^257^ ^]^ To this end, streptavidin‐coated polystyrene microparticles with the size of 5–7 µm were conjugated with biotinylated human or mouse PD1 as a competitive inhibitory strategy with the PD1 on the surface of the TCs. In vivo studies showed that when a single dose of the particles was injected directly in the tumor (but not intravenously) of the melanoma‐bearing animals, a 4.6‐fold decrease in the tumor volume was observed in the group receiving the PD1‐decorated particles.

In general, being inspired by the leukocytes, artificial immune cells have developed to mimic several characteristics and functions of their natural counterparts. On‐demand shape‐morphing, triggered‐motility, long circulation, and programmed interaction of leukocytes with various cells, including natural immune, endothelial, and pathologic cells, have been the main source of inspiration to develop therapeutics based on artificial immune cells. These systems can work as effective, specific, and safe APCs to activate/inhibit the host's immune system for the treatment of cancers, autoimmune diseases, or engrafted tissue rejection in the context of personalized medicine.^[^ ^231^, ^258^ ^]^ In addition, cell membrane can be used to develop TC‐mimicking artificial cells for the development of theranostic carriers with laser irradiation‐mediated endosomal escape property and NIR‐dependent drug release.^[^ ^259^ ^]^

## 7 Conclusion and Future Perspectives

Emerging realms in the fields of science and technology have revolutionized our understanding of health and disease. Investigations in biomaterial science, immunology, biology, genomics, proteomics, and metabolomics have given birth to the concept of synthetic biology. Currently, the “one‐size‐fits‐all” therapeutic approach is one of the dominant practicing paradigm. This may become achievable in the near future considering the diverse and stealth feature of the cell‐mediated therapies now available to answer the growing demand for more effective and safer diagnostics and therapeutics. Although still in their infancy, it is believed that combining current trends in biological engineering, cell therapy, nanotechnology, and material chemistry can result in therapeutics based on the immune cells. These can possess extremely diverse platforms with great therapeutic potentials for superior personalized diagnostics and therapeutics.^[^ ^260^ ^]^ In this comprehensive review, we have focused on the many ways immune cells have inspired developing novel therapeutic platforms and the latest advances in the field are presented. The unique properties of leukocytes, including their ability to recognize various cells and biomolecules, their potential to navigate and patrol through the body, their outstanding antigen presenting ability and potential to send stimulatory/inhibitory signals to their adjacent and distant cells, and also being active against pathogens and tumor cells, are discussed. From a material‐concerning perspective, these leukocyte‐inspired therapeutics fall into five main categories: therapeutics based on alive immune cells, surface engineered immune cells, those taking advantage of the leukocyte's membrane, systems based on the ECVs being secreted by the leukocytes, and artificial immune cells. These systems could be utilized as therapeutics by themselves, as they contain a variety of biomolecules (e.g., DNA and proteins), or act as a vehicle for targeted delivery of diagnostics and therapeutics to the disease site. In addition, it has been highlighted that the immune cells can be manipulated by various genetic engineering methods, as well as using a wide range of micro/NPs and small molecules. Exploiting such technologies empowers the developed systems to respond to their environment automatically and on‐demand to control the biological functions in hard‐to‐treat diseases. For example, some of the immune cells, like proinflammatory M1 macrophages, show tumor targeting abilities. Therefore, engineered alive M1 macrophages, M1 macrophage‐derived exosomes (MDEs), and M1 macrophage‐membrane‐coated NPs can all be used to target solid tumors, and even cancer metastasis. Nevertheless, shortcomings and challenges of the leukocyte‐based therapies must be considered at all levels, including research and development, manufacturing, clinical application, and even regulatory. First, most of the mechanisms of immune cell recruitment and phenotype alteration in the tumor microenvironment or inflamed tissues have not been fully understood due to the high complexity of the damaged tissues and the dynamic nature of the immune system. Therefore, it is essential to monitor the effect of immune‐cell derived microrobots and biomimetic nanoparticles in real‐time. Molecular imaging with immune cell targeting probes, which bind to surface proteins, would be helpful to identify the distribution and polarization of immune cells in damaged tissues via high spatiotemporal resolution. Second, due to the complicated microenvironment of hard‐to‐treat tissues in cancer, autoimmune diseases, neurodegenerative disorders, cardiovascular complications, and infectious diseases, drug delivery by immune cell‐derived carriers might not be fully effective in all cases. To solve this problem, a good strategy will be the combination of drug delivery with radiotherapy, photothermal therapy, photodynamic therapy, cell therapy, and/or immunotherapy (e.g., anti‐PDL1 therapy and CAR T‐cell therapy). Such multitherapy approaches will be beneficial since they avoid serious side effects. Third, the targeting ability of current immune cell‐derived carriers is still limited, which in the future, advanced gene‐editing technologies like CRISPR‐Cas9 or conjugation of targeting ligands can render stronger targeting efficacy to the cellular microrobots.

To translate research outcomes to clinical application for leukocyte‐based therapeutics, some challenges should still be addressed. For example, while engineered immune cells and cellular microrobots hold great promise for clinical translation, differences between ex vivo experimental conditions and the patients's immune system must be taken into consideration to not overestimate in vivo efficacy of a developed formulation. In addition, large‐scale production of universal cellular carriers is difficult since they should be suitable for the majority of people. To achieve this aim, immune cell extraction should be performed in humans, and genes related to immunological rejection must be excluded by cellular engineering to avoid unnecessary side effects. It should be considered that in many cases and from an economical point of view, the more personalized a treatment is, the higher the cost it takes to be manufactured. Therefore, developing novel technologies for handling biomaterials and alive cells with high yields can significantly reduce the costs of such therapeutics. Therefore, large‐scale production technologies need to be developed, new quality control setups should be proposed, and redesigning of the rules and approval requirements should be taken into account by regulatory agencies. Such standard protocols and processing approaches will reduce batch‐to‐batch variability.

When all the above‐mentioned challenges are addressed, immune‐cell derived microrobots and biomimetic nanocarriers will be strong “weapons” to fight against challenging hard‐to‐treat diseases. To support this claim, it would be worthy to point out that two CAR‐TCs have received FDA‐approval for the treatment of leukemia and this implies the potential of such leukocyte‐based treatments to be translated into clinical setting, despite their current astronomical costs.^[^ ^261^ ^]^

Overall, considering the important role of leukocyte‐based therapeutics in ameliorating currently untreatable disorders, as well as growing investment in the research and development of this field, it seems that immune cell‐based therapies will shape the future of personalized medicine. However to apply this research, a top priority of future studies will be to ensure the safety of immune cell‐based therapies.

## Conflict of Interest

The authors declare no conflict of interest.
